# How long does biomedical research take? Studying the time taken between biomedical and health research and its translation into products, policy, and practice

**DOI:** 10.1186/1478-4505-13-1

**Published:** 2015-01-01

**Authors:** Stephen R Hanney, Sophie Castle-Clarke, Jonathan Grant, Susan Guthrie, Chris Henshall, Jorge Mestre-Ferrandiz, Michele Pistollato, Alexandra Pollitt, Jon Sussex, Steven Wooding

**Affiliations:** Health Economics Research Group, Brunel University London, Kingston Lane, Uxbridge, UB8 3PH UK; RAND Europe, Westbrook Centre, Milton Road, Cambridge, CB4 1YG UK; King’s Policy Institute, King’s College London, Virginia Woolf Building, 22 Kingsway, London, WC2B 6NR UK; Office of Health Economics, 105 Victoria Street, London, SW1E 6QT UK

**Keywords:** Basic research, Clinical guidelines, Discovery research, Economic impact of research, Human research, Pharmaceutical industry, Process marker model, Regulatory approval, Time lags, Timelines

## Abstract

**Background:**

The time taken, or ‘time lags’, between biomedical/health research and its translation into health improvements is receiving growing attention. Reducing time lags should increase rates of return to such research. However, ways to measure time lags are under-developed, with little attention on where time lags arise within overall timelines. The process marker model has been proposed as a better way forward than the current focus on an increasingly complex series of translation ‘gaps’. Starting from that model, we aimed to develop better methods to measure and understand time lags and develop ways to identify policy options and produce recommendations for future studies.

**Methods:**

Following reviews of the literature on time lags and of relevant policy documents, we developed a new approach to conduct case studies of time lags. We built on the process marker model, including developing a matrix with a series of overlapping tracks to allow us to present and measure elements within any overall time lag. We identified a reduced number of key markers or calibration points and tested our new approach in seven case studies of research leading to interventions in cardiovascular disease and mental health. Finally, we analysed the data to address our study’s key aims.

**Results:**

The literature review illustrated the lack of agreement on starting points for measuring time lags. We mapped points from policy documents onto our matrix and thus highlighted key areas of concern, for example around delays before new therapies become widely available. Our seven completed case studies demonstrate we have made considerable progress in developing methods to measure and understand time lags. The matrix of overlapping tracks of activity in the research and implementation processes facilitated analysis of time lags along each track, and at the cross-over points where the next track started. We identified some factors that speed up translation through the actions of companies, researchers, funders, policymakers, and regulators. Recommendations for further work are built on progress made, limitations identified and revised terminology.

**Conclusions:**

Our advances identify complexities, provide a firm basis for further methodological work along and between tracks, and begin to indicate potential ways of reducing lags.

**Electronic supplementary material:**

The online version of this article (doi:10.1186/1478-4505-13-1) contains supplementary material, which is available to authorized users.

## Background

The time taken, or ‘time lags’, between biomedical and health research and its translation into interventions that lead to health and wider benefits is a topic of growing interest and investment by those concerned to maximise the returns from such research [1–3]. Once innovations have been produced, then aspects of their diffusion have long been studied [4–10] using a wide range of disciplinary approaches [11].

The literature exploring the full time lags between early research and the eventual translation into health gains is less extensive than that focussing specifically on the diffusion of innovations. Key questions remain to be addressed. Balas and Boren [12], Grant et al. [13], and Wratschko [14] all estimated a time lag between research and clinical practice of 17 years, but did so while measuring different, if overlapping, parts of the process. Such convergence around an ‘average’ time of 17 years hides complexities that are relevant to policy and practice and would benefit from greater understanding. Morris et al. [15] summarised the existing literature and found that the variation in choice of measures (often proxies for the time between research and its translation) meant that studies were rarely measuring the same thing; this variation made aggregation, comparisons, and generalisations difficult. Furthermore, little attention appears to have been given to understanding where within the overall timeline the time lags are most likely to occur, and the variations that arise.

Existing models of research translation typically refer to the concept of one or more translation ‘gaps’ along the overall pathway [2], but how these gaps are defined differs significantly between approaches and can lead to results not being comparable between studies. An alternative approach has been proposed in the “process marker model” developed by Trochim et al. [16]. Here, specific research translation milestones or events are considered to be process markers, and are clearly defined to enable comparability. Durations can then be assessed between these markers.

Developing improved ways of measuring time lags is important because, as Buxton et al. [3] quantified in a previous study, shortening timescales from research to benefits increases the rates of return achieved from the resources invested in research, other things being equal. There is the risk, however, of inappropriate attempts to reduce timescales unless there is a proper understanding of the true nature and variation of time lags in the translation of health research. For example, there is a need to acknowledge that it is important to test the safety, efficacy, effectiveness, and value of health care innovations, and this testing takes time.

The development of medicines is a major area where various phases of research and development are necessary, and the time taken to develop new medicines is an issue of considerable concern, not least because of its impact on costs [17]. The literature on development times for medicines suggests that durations between Phase I and Phase III clinical trials have changed little over time, but there are significant differences between different therapy areas [18–21]. Some suggested ways to reduce the time taken to translate research raise questions about whether the changes merely result in the time being reduced for some interventions at the expense of lengthening it for others, as a result of prioritising the translation of one intervention over another.

There is, therefore, considerable potential value in further analysis of time lags in order to generate increased understanding of the range of factors at work. In the study reported here, we set out to analyse the best ways of conducting further analysis of time lags between research and its translation into improved health care, and decided to develop and use a case study approach, building on the insights from Trochim et al. [16]. The specific objectives included i) developing methods to facilitate improved measurement, and enhanced understanding, of the diverse nature and causes of time lags across different medical fields and types of interventions; ii) developing ways to identify factors that speed up translation of research and options for addressing key policy concerns with the long-term objective of achieving the more rapid health and economic gains that could result from faster translation of research into practice; iii) provide a sounder basis for the assessment of the rate of return on the investment of biomedical and health research; and iv) making recommendations for further possible studies.

We adopted a range of methods. First, we conducted two brief reviews. These reviews covered i) the literature on time lags, and ii) relevant policy documents and reports. Second, we developed a new approach to conducting case studies of time lags that started from the process marker model but built on it in significant ways – since this is a methodological study we describe the new approach in some detail. Third, we applied and tested our new approach in seven case studies of interventions in cardiovascular disease (CVD) and mental health. Fourth, we developed a range of approaches to analyse the data gathered.

The Results and discussion section presents the findings from our reviews of both the literature and the policy documents, before describing the progress made in the seven case studies conducted to test the applicability of the methods developed. As part of this, we describe how far the cases can be reported using the matrix we developed to aid comparability of research findings. We then analyse how far the findings enable us to address the specific objectives of the study. Finally, we make recommendations for future analysis of the time taken to translate biomedical and health research into practice.

## Methods

In this methodological study there was considerable iteration between the methods at various stages and emerging findings. Inevitably, the whole process was more complex than is presented here, but we set out key features of the approaches developed and applied.

### Reviews of the literature on estimations of time lags and the potential policy responses to the problem of excessive time lags

The objective of the literature review was to inform the overall study as to what had previously been claimed about the length and nature of time lags. The review aimed to do this by updating the review by Morris et al. [15] of the literature describing and quantifying time lags in the health research translation process, and expanding it to cover areas relevant for the private sector. The (non-systematic) search strategy was adapted from O’Neill [22] and conducted using Google Scholar, Web of Science, PubMed, and EBSCO, based on key words. Potentially relevant publications were identified through a two-step bootstrapping approach. In the first step, we adopted the same key words used in Morris et al. [15] to define “time lags”, and the words suggested by the team’s experience to define “research” and “private sector”. In the second step, the key words were adjusted to identify the more relevant hits based on titles.

The primary purpose of the review of policy documents was to identify perceived reasons for lags and some measures that have been taken to reduce lags where they were deemed excessive. From the outset we realised that while relevant research could be international, when it came to regulations and policies we would have to have a primarily UK focus so as to keep the project within budget and timescale. Drawing on the team’s existing knowledge of the field, key UK policy documents from 2002 were identified (plus major US documents they cited). Each document was searched for the terms: lag, delay, accelerate, speed, and time. Discussions in each document were then summarised, with selected quotes, under the headings: ‘reasons for time lags’ and ‘policy measures to address’. Subsequently, the key lags discussed in each document were mapped onto the various tracks (or stages/phases) of the translation process that were developed in the matrix described below.

### Development of concepts and tools for conducting case studies and presenting findings on time lags

There are currently few methods that can be applied directly when undertaking case studies on time lags. We therefore drew on the experience of team members in conducting case study research of the impact from health research [23–25], and in analysing medicines development pathways [17].

#### Analysis of process marker model and identifying areas for amendment

Based on the above experience, we analysed the advantages and disadvantages of using the process marker model as the framework for conducting detailed case studies. We concluded that the process marker model, as it stood, would not meet the needs of our research. We present the key aspects of our analysis of the process marker model here, before describing the methods we went on to develop. In the Results and discussion section we further analyse the contribution of the various methods considered.

Trochim et al. [16] identified various ways in which their process marker model is helpful. Namely, it provides a more consistent way of analysing the research and translation process than the multiple models and translation gaps (“T gaps”) proposed by others; it provides a way of avoiding unnecessary debate about the start point of the overall process because its main focus is on the processes between the first and last markers chosen to be measured; and it provides opportunities for detailed study of small segments of the overall timeline that can then be combined if required. Furthermore, a key element of the Trochim model is the series of ‘*operationally definable*’ markers which are the milestones or events in the ‘*research-practice translation continuum*’, each of which ‘*can be operationalized as a specific date*’ ([16], pp. 159–160). Finally, the model suggests three levels of process with the top level including the full range of the continuum from the basic research system on the left hand side, to the right hand side that depicts ‘*translation to practice and policy, and ultimately use in populations and the health of the public*’ ([16], p. 159). The markers at this top level include pilot proposal submitted, pilot study publication, first study in animals, first study with humans, Phase II clinical trial started, patent applied for, Food and Drug Administration approval, inclusion in research synthesis, dissemination research is included in health policy, and health impacts measured. Then, more markers are suggested for the two more detailed levels below the top level.

However, in planning to apply the process marker model to our case studies, including a number of non-pharmaceutical interventions, we found that a full analysis of a timeline using the range of process markers illustrated in Trochim et al. [16] would require the collection of unfeasibly large amounts of data which, even if available, would take a lot of time and resources. Further, as presented, the model appears to assume there will be one key study at each stage of the process; we found that this is a more reasonable assumption for interventions, such as a specific medicine, than it is for others, such as psycho-social type interventions, for some of which there was a family of studies spanning a wide range of times. Additionally, the model does not explicitly recognise the international nature of much of the relevant research and the challenges of tracking and capturing it. While Trochim et al. [16] recognise that the research and translation process is complex and there may be feedback loops, the linear character of the model as presented implies an inexorable move from research to translation to impacts and does not easily recognise or capture the fact that translations at some key points requires work to cross over from one track of work and people to another.

In response to these concerns about the process marker model, we undertook further methodological development, as described below.

#### Devising a matrix with multiple tracks to present the case study material

**Figure 1:**
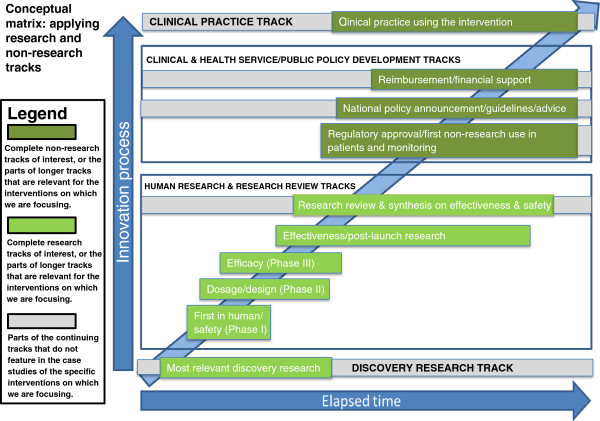
**Conceptual matrix for measuring and understanding time lags.**

We aimed to develop an approach that would allow the presentation of data about multiple tracks (or stages or phases) in the translation of research into healthcare improvements in a way that was not simply linear. These tracks/stages/phases have some similarities with some of the markers in the top level of Trochim et al.’s model [16], but aim to recognise the importance of incorporating scope for overlaps. We considered each of the major steps in the process of translation from early research through to adoption in the healthcare system, and attempted to build them into a matrix that could be applied to the various fields of research and types of intervention. The matrix (Figure 1) was refined in response to the emerging findings from the case studies, and further iterations between team members. It consists of four main groups of tracks, two of which contain the research (discovery research and human research/research review) and two of which cover the clinical practice and public policy developments. The two middle groups each consist of a number of separate tracks.

As shown in the Figure, some tracks (for example discovery research, research review and synthesis, policy development, and clinical practice) have an ongoing “life” of their own with activity in the track often pre-dating and continuing after key events that cause work on a specific intervention to commence or cross from one track to another. For example, discovery research not only pre-dates the development of an intervention for human use, but continues after an initial intervention has been developed and entered into testing, and may continue to influence the development of that intervention. Similarly, policy on clinical practice may pre-date the arrival of a specific intervention, will develop or adapt in the light of increasing evidence on the effectiveness of that intervention, and will continue to develop even after evidence on the intervention is completed, as newer interventions or approaches come on stream. In practice, as illustrated on Figure 1, some intervention-specific tracks such as “effectiveness/post-launch research” might well continue after the “later” tracks in the matrix have started so it is likely there will be various overlaps in the tracks in the matrix from any specific intervention.

A model of this kind facilitates analysis of time lags, and policy responses to them, by allowing consideration of two linked issues. These are the time and actions required to move research and its translation i) along any particular track, and ii) from one track to another. It can also facilitate demonstration of examples where the translation process might stall and need to move back down to a lower track for the work required to overcome the barriers encountered.

#### Identifying markers/calibration points

We started by exploring a wide range of possible markers (that is: milestones or events) from both Trochim et al. [16], who had stated that their quite extensive list of markers was only illustrative, and a range of other sources. We identified a long list of nearly 200 potential markers from Trochim et al. [16] and a range of other papers [15, 17, 26–29], and reviewed it, removing (near) duplicates and adding to the list as the team saw fit. In the first iteration we reduced the list to around 130. While it was clear that this list would need to be further reduced, the applicability of the general approach was tested by attempting to apply some of the key markers to data that had been gathered by some of the team members in a previous payback case study. This was an analysis of the impact from the early research conducted by Mont Liggins on the use of corticosteroids for the prevention of respiratory distress syndrome when pre-term delivery was expected [27].

This initial test of the applicability of the general approach was reasonably positive, and encouraged us to develop the approach further. It was clear that, with the resources available for data collection, the number of markers would have to be slimmed down. In finalising the shortened list, we balanced three main criteria in selecting what we called calibration points. As far as possible they should be: unambiguous in terms of having a clear date; key points for measuring time lags and understanding their nature (and, if possible, for helping identify their causes); and obtainable from information that is generally available. Most of the markers in the long list could, as noted above, be “operationalized as a specific date”. Therefore, they often already had the quality of meeting our definition of being unambiguous. In selecting calibration points, a balance of the other criteria was also important, with the ability to identify a start point for each track increasingly recognised as being particularly important. Wherever possible, we looked to identify the calibration points from among the markers at the highest of Trochim’s three levels [16], and for the implementation tracks we also drew on the expertise of team members to confirm the most appropriate calibration points. We present our final list of 11 calibration points below, where they are arranged in a way that reflects the tracks from the matrix in Figure 1. It is important to note that some of the calibration points will re-occur in several different research tracks, and some calibration points might occur more than once in the same track. Also, we separately list two different types of review paper.

#### Final list of 11 calibration points selected for use in the case studies (linked to tracks from the matrix)

*Research tracks (i.e., non-human; human or sub-divisions; research review and synthesis)*

i. Start point of key research study: date proposal submitted to external funder, or often internal if pharmaceutical studies, etc.
ii. Start of data collection: date of first patient recruited, or equivalent in basic research (this could be an alternative start point of key research study if proposal is not available).
iii. Date of main publication of study findings: could be more than one for same body of research where more than one has been significant in translation or in the timeline; also could include an equivalent presentation of findings from basic research conducted in companies where no formal publications occur.
iv. Patents: date of filing and approval (first, and UK if different).
v. Date of key review paper: key review of basic or early research seen as a crucial step in translation.
vi. Date of internationally recognised systematic review of randomised controlled trials (RCTs) or equivalent (need to include the first, and the first in the UK if different and relevant for implementation, and also others if they are particularly relevant in implementation).

*Non-research or implementation tracks*

vii. Date of regulatory approval for UK, from the relevant body.
viii. Date of first use in UK patients in a routine/non-research context: which can be launch in UK/first sale where the technology is sold in the UK.
ix. Date of announcement about national policy to introduce intervention, or guideline/advice issued: need to include the first guideline relevant in the UK, and also others – UK or international – if they are particularly relevant in implementation, including key updates.
x. Date of announcement about UK policy to reimburse or provide financial support: reimbursement decisions can be identified where publication of a NICE technology appraisal.
xi. Intervention becomes standard practice: will vary by intervention.

Our approach facilitated the collection of international data, especially for the research tracks. Data for the implementation/health service tracks focused primarily on markers and calibration points relevant to the UK. We discuss the implications of this later.

### Undertaking the case studies

#### Selection of case studies

We planned to select six case studies, three from each of CVD and mental health. These were the two medical fields on which we focused in previous studies, firstly to assess the value of UK medical research [3], and secondly, to analyse the range of impacts from medical research [30]. We selected our case studies to include key examples of UK-funded research. In each field we aimed to select a range of interventions, so that overall we included examples of pharmaceuticals, screening, public health, psychosocial behavioural interventions, and service organisation/complex interventions. In addition to these criteria, we also wanted to make as rapid progress as possible in this exploratory study and, therefore, identified case studies where we had reason to think we could make progress. We identified two pharmaceutical examples where there had been important UK research, and initial correspondence with contacts in the industry indicated the feasibility of these studies. In CVD, we selected abdominal aortic aneurysm (AAA) screening as a stream of research with a major element of Medical Research Council (MRC) funding plus involvement of team members, and smoking reduction as an area that overlapped with team members’ earlier work on the value of medical research [3]. In mental health, we drew on examples from the Mental Health Retrosight project being conducted by RAND Europe members of the team to assess the impact of examples of mental health research [31]. It became clear that the mental health case study on cognitive behavioural therapy (CBT), which was considering two applications (in depression and in schizophrenia), was more appropriately presented as two separate case studies, thus giving a total of four mental health case studies. Our seven selected case studies are shown in Table 1.

**Table 1:** **Seven selected case studies**

Case study	Cardiovascular disease	Mental health	Type of intervention
One	Calcium channel blocker – amlodipine		Pharmaceutical
Two		Atypical antipsychotic – olanzapine	Pharmaceutical
Three	Screening for abdominal aortic aneurysms		Screening
Four	Smoking reduction		Other public health
Five		Cognitive behavioural therapy for depression	Psychosocial
Six		Cognitive behavioural therapy for schizophrenia	Psychosocial
Seven		Early intervention for schizophrenia (or “early intervention”)	Service configuration

#### Data collection

We tested three different approaches for collecting case study data. The first approach broadly replicated that adopted previously in payback studies and involved archival and documentary review and interviews [23–25]. Second, for the pharmaceutical studies, we collected information from several sources, including contacts in the pharmaceutical industry, publications, and reports of court cases about patent disputes (this last, a key source that emerged during the conduct of the case studies). Third, a bibliometric approach was used, where appropriate, to identify publications from key research studies and then to examine key systematic reviews and guidelines from the National Institute for Health and Care Excellence (NICE). This approach was applied in case studies derived from the Mental Health Retrosight project [31], where it was possible to build on case studies in which at least some of the key research studies had already been identified.

### Analysis

We had assumed it would be difficult to draw robust policy conclusions from analysis of seven case studies, but thought nonetheless that it was important to see if the methods being developed had the potential to inform policies, and what further methodological steps might be necessary to allow studies to be undertaken to support valid conclusions about policy. To do this we undertook several steps. First, team members considered the set of case studies and attempted to draw out lessons about the nature and causes of any time lags identified, and about the methods used to assess those time lags. SH then synthesised the comments from team members. These approaches were supplemented by a quantitative analysis conducted by SG of the time elapsed in each case study. For this she used the various tracks identified in the matrix (Figure 1) and attempted in each case study to identify the time spent on each track before activity in the next track started.

Data from the review of literature and policy documents, the case studies, the synthesis, and the quantitative analysis were brought together for consideration at team meetings and at a seminar held at the Office of Health Economics at which an audience of stakeholders from academia, industry, and policy advisers was invited to comment on the emerging findings.

The necessary ethical approval for the study, in which a series of interviews were planned, was obtained from Brunel University’s Ethics Committee.

## Results and discussion

The key findings from the reviews of the literature and the policy documents, and from the case studies, are set out below in turn, with further details available in the relevant Additional files. We then report and discuss the findings related to the analysis of the objectives set out in the Background, including making recommendations for future possible work. As this is a methodological study, a range of limitations are discussed at various points as they arise in the analysis.

### Reviews of the literature on estimations of time lags and potential policy responses to the problem of excessive time lags

The findings from the literature review are similar to those in Morris et al. [15], in that the papers we identified do not measure time lags in a comparable way. Importantly, all of the studies we identified focus on pharmaceutical R&D, making it easier to compare the methodologies used by the authors to estimate the time lags. However, this also implies that any conclusion derived from this literature review might be specific to case studies of medicines and might not apply to the development even of other commercially produced treatments, let alone other areas of biomedical and health research and development. The full review is presented as Additional file 1.

Despite the specific focus on medicines, it is still the case that there was no general agreement on which start point to consider. One of the reasons why some authors prefer to consider preclinical and clinical start points, ignoring a significant part of the R&D needed to bring a drug to the market, is that there are several potential ways to trace the birth of a product idea [32], and so the definition can be arbitrary and require additional specification. Chandy et al. [32] found that the mean time between patent filing and product launch varies considerably according to the therapeutic area. The mean time ranges from 8.5 years for anti-infectives to 15 for immunological medicines. This result may be due to scientific barriers to technical development in a particular therapeutic area and also to specific regulatory policies to favour the research in areas of great unmet need. This suggests that even studies using the same time points to estimate the development lags of drugs may produce very different results depending on the set of medicines analysed.

We found from our review that there is more homogeneity in choice of end points, which usually refer to the easier to record licensing process. This more common end point, however, occurs somewhat earlier in the whole translation process than the end point indicated in Trochim et al.’s model, or included in our matrix. Mestre-Ferrandiz et al. [17] compared the intervals between the preclinical and clinical milestones used by the Centre for Medicines Research International with the more standard Phase I to III trials. The literature review thus provides additional analysis that highlights the variety of approaches used in previous assessments of time lags, and reinforces that it is still a relatively underdeveloped field.

**Figure 2:**
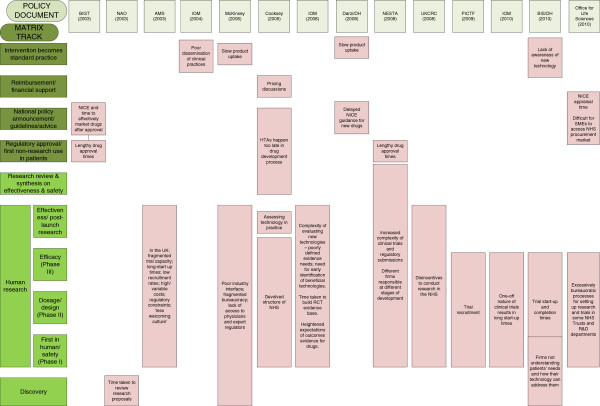
**Using the matrix to summarise time lags identified in policy documents.**

In the review of policy documents, we identified a range of reports as being relevant since they discussed issues related to time lags. Those that highlighted key time lags [1, 33–44] are summarised in Figure 2, in which the lags are organised according to the matrix we developed and described earlier in the project (Figure 1). Additional file 2 includes the full review of these policy documents.

Although these lags covered the full range of “tracks” we described in our matrix, many of the documents focused particularly on the challenges of conducting human research, including delays in starting clinical trials, difficulties in collaborating within the healthcare system, and the complexity of regulation in this area. A second area of focus was the time taken for new medicines and technologies to become widely available, both in terms of the time taken for approval or appraisal by NICE, and slow subsequent uptake in the National Health Service.

A range of measures, both actual and aspirational, aimed at reducing lags was also discussed in these documents. These measures included creating structures to facilitate collaboration, streamlining legislation, and a consideration of the incentives for different stakeholders to participate in biomedical and health R&D. In some cases policy changes have already been made to introduce proposed measures.

### The case studies

We wrote up each of the completed seven case studies according to an overall common framework starting with a brief narrative account including i) a definition of the intervention and its role, and the background to its development, and ii) key issues related to time lags, including discussion of any long time lags in the case study and, where relevant, examples where activities were undertaken or policies implemented in an attempt to reduce time lags. This is followed by a timeline account that recorded each of the relevant events, for example a trial in one of the research tracks, and the various markers or calibration points that could be applied to it. Next there is a version of the matrix with the various tracks populated with key events and calibration points from the specific timeline, and, finally, a reflection on methodological issues that arose during the conduct of the case study, or in later consideration of the processes.

**Figure 3:**
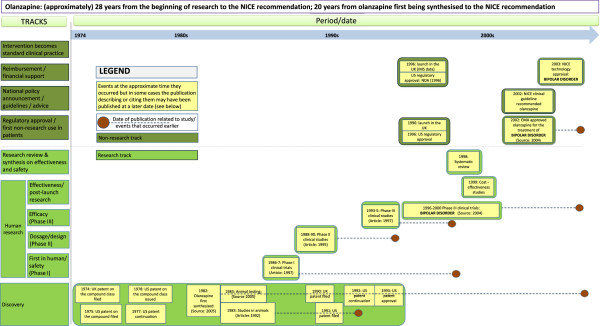
**Example of a case study matrix: olanzapine.**

The full case studies are available in Additional file 3. The range of methods used resulted in the collection of a considerable amount of data. A simplified version of the matrix from the olanzapine case study is shown below in Figure 3, in which the activities related to its use also in the treatment of bipolar disorder are separately identified for three tracks, and while the full case study develops various points in more detail, Table 2 illustrates a number of key points from the case.

**Table 2:** **Key points illustrated by the olanzapine case study**

Track	Point illustrated
Discovery track	Activity in this track continued even after several human studies had been conducted
Research tracks	The publication sources linked to several key events occurred well after the event itself and sometimes after activity had already started in the next track: the publication source for the animal testing was a later court case over a patent dispute; the Phase I trial was described in a later account of the stream of research; and the Phase II trial was described in a paper published sometime after Phase III had started
National and clinical policy guidelines track	While there may appear to be quite a long delay between the launch of the medicine in the UK in 1990 and the NICE recommendation in 2002, it should be remembered that NICE only began publishing clinical guidelines shortly before 2002
Overall	In the case study it has been possible to develop a matrix to illustrate the movement from the early research to the NICE recommendation over a 28-year period and involving activity in most of the tracks

Additional file 3 shows how we were able to use the matrix we developed to present the data from each case study. In some cases not all the tracks proved to be relevant, but the matrix was at least somewhat applicable to all seven case studies.

While there were resource problems with collecting the data on even our more limited range of calibration points, the pharmaceutical cases in particular identified novel ways of data collection through the use of published information about court cases on patent disputes. It should not be expected that this source of information will be available in all the cases, but this does illustrate the important methodological finding that the framework we developed allows data to be gathered in innovative ways where opportunities arise. Furthermore, in the pharmaceutical cases, institutional arrangements provided some clear milestones for which dates could be identified, i.e., the patenting and regulatory procedures, and using our approach we were able to take full advantage of this to demonstrate the nature of the time lags.

Finally, the experience of conducting the case studies helped us identify a range of considerations, such as, while the matrix was very useful, it does not allow full presentation of some key points that have been identified, and therefore further narrative highlighting of key issues was required. Further, to identify where policy action will be most fruitful requires a micro-level analysis of just why a particular lag occurred and whether that was desirable in the specific context of the specific technology. As noted, one of the additional aims of the project was to test the process marker model proposed by Trochim et al. [16]; it became clear to us fairly early in the project that the number of markers in the model would pose unreasonable challenges in terms of data collection. It also became clear that each “marker” could occur more than once in a particular story of development and that the overlap between different stages could be important in understanding the process of development. This led to the development of our own matrix/track structure that we used both for data collection in the case studies and, as set out below, for data analysis.

### Progress in developing methods to improve measurement and enhance understanding of the diverse nature and causes of time lags

Analysis of the case studies suggests considerable progress has been made in developing methods to improve the measurement and understanding of time lags. The pattern of time lags associated with each intervention could be identified, organised, and displayed in the narrative account, timeline, and matrix for each intervention. The matrix is an advance on the single linear model, and the need to populate the various tracks within the matrix focused the data collection. It was possible to explore time lags along the tracks, in addition to lags arising at the cross-over points between tracks.

The usual limitations facing many research studies of achieving a balance between breadth and depth were exemplified in this study because the resources required to conduct seven case studies were considerable, and yet, to varying degrees, they may not be complete. The seven case studies demonstrate the complexity of the issues addressed, and the variety of circumstances in which they arise. While a range of interesting and potentially useful observations can be made, generalisations of any sort are difficult to make. Below, we first discuss the progress made in developing ways to measure time lags, and then progress in enhancing understanding.

#### Measuring time lags

In all case studies there was a considerable period between the start of the research and the contribution to improved health. The time lag is generally longer than the estimates of time lags reported in the Background section. There are two main reasons for this. First, at least some of those studies (for example Grant et al. [13] and Buxton et al. [3]) reported that 17 years was the average time lag for all the studies in a body of research cited in clinical guidelines. We are analysing something different and something where one would expect there might well be longer durations, namely the whole timeline in the translation of a specific intervention from the initial basic, or discovery, research through to the health benefits. Second, when measuring whole timelines there is considerable variety in the points between which time lags are measured in different studies, and many of the previous assessments of time lags used a more restrictive definition for appropriate start and finish points than those on the full spectrum set out in Trochim et al.’s process marker model [16].

In our case studies, we attempted to go back to a starting point that seemed as though it would facilitate a reasonably comprehensive analysis. However, inevitably, the selection of a start point is to some extent arbitrary. The availability of information, and hence the starting point for each study, varied and our studies again highlight that the start point taken for the analysis of time lags has a major impact on the total duration identified.

In an attempt to give some comparability across the case studies, our quantitative analysis of the overall time lag focused on the time lag between “[intervention] discovery” and start of widespread implementation, defining discovery as the moment at which a clear intervention is defined and selected for testing. This is typically the point at which the intervention is linked with the relevant condition. For a medicine it would be the point at which it is first synthesised (provided it is synthesised with the intention to use it for the condition for which it is implemented). In smoking reduction, there are various points that could be chosen and two examples are presented herein, one from the first evidence linking smoking and ill health, and another starting from the first evidence on passive smoking. The timelines and matrices in some case studies (as in the matrix for olanzapine contained in Figure 3) show a period of activity in the discovery track that preceded the point at which the quantitative analysis of the time lag classified the intervention as being discovered in the sense of a clear intervention being defined and selected for testing. The start of widespread implementation is the point at which a concerted effort at national policy roll-out occurs in the UK. While the early part of the matrix covers research internationally, for policy statements and implementation the analysis focuses on the UK for comparability and to limit the extent of information gathering required (though the wider approach could equally well be applied to first policy statements and implementation internationally). Table 3 demonstrates how our approach enables measurement of overall time lags in a reasonably consistent manner across case studies in different fields and looking at different types of intervention. It is important to note, however, that some of the numbers on Table 3 are estimates because we are not sure about the exact dates of key events. Furthermore, in practice, the case studies did not generally get as far as assessing health gain and this was not included in the matrix or as a calibration point.

**Table 3:** **Quantitative summary of time lags – years from “discovery” to UK implementation**

Case study topics	Overall time lag in years from “discovery” to implementation (ie not always counting some initial discovery time)	Field: cardiovascular disease (CVD) or mental health (MH)	Intervention type	Lag from “discovery” to start of first phase I trial or human research	Lag from phase I to start of first phase II trial	Lag from phase II to start of first phase III trial	Lag from start of phase III or human research to first research review and synthesis	Lag from research review and synthesis to first policy statement	Lag from policy statement to implementation
**1: Amlodipine**	23 years	CVD	Pharmaceutical	3	1	2	10	7	0
**2: Olanzapine**	20 years	MH	Pharmaceutical	4	2	5	5	4	0
**3: Abdominal aortic aneurysm screening**	26 years	CVD	Screening	0	1	5	14	5	1
**4a: Smoking reduction: Evidence on passive smoking to widespread bans**	39 years	CVD	Public health	14	n/a	n/a	5	19	1
**4b: Smoking reduction: Link between smoking and ill health to ban on advertising (Top TV ban, Bottom widespread ban)**	26 years	CVD	Public health	12	n/a	n/a	6	8	0
	54 years							30	6
**5: Cognitive behavioural therapy and depression**	49 years	MH	Psychosocial	15	n/a	n/a	12	12	10
**6: Cognitive behavioural therapy and schizophrenia**	48 years	MH	Psychosocial	17	n/a	n/a	23	0	8
**7: Early intervention for schizophrenia**	18 years	MH	Service configuration/Health service delivery	6	n/a	n/a	Reviews after implementation	7	5

Our approach not only attempts to measure overall time lags, but also many of the individual elements within the overall lag. Such measurements help to understand the nature and causes of the overall time lags, and work out ways to reduce them. Using a series of key points as the calibration points, mostly the start of activity on a series of tracks, Table 3 allows us to measure individual elements within overall time lags. It is possible to use this approach to make comparative measurements of time lags across the different stages of the research and implementation process, drawing on the matrices and the timelines in the case studies. In some cases a stage is skipped – for example in “early intervention”, where a policy statement was made before the first research review and synthesis was published.

The most significant advance using our new approach is that while the measurement does go along a track, the length of the time of the element is not determined by any fixed point along that track but instead is decided by when activity in the next track starts. For example, in the AAA screening case study the community cohort study was regarded as the equivalent of a Phase II trial [45]. The study started in 1984 and did not report until 1991, however, the first RCT that could be viewed as a Phase III trial began in 1989. Therefore, the time lag element for that phase was counted as being 5 years rather than 7.

There are implications of the fact that the end point for the part of each track we measured as contributing to the overall time lag was not a defined marker, or calibration point (for example, a publication), in relation to that particular track, but instead was the start of the next track. First, it means that given as we move along a track there is no fixed end point for the measurement of what is counted as being the time lag element of any track, then the end point dictated by the start of the next track might occur well before, or well after, the relevant publication for the original track. Second, it means that the headings used for the items in the columns on Table 3 showing the quantitative analysis of elements within the overall time lag present a somewhat different picture from that set out in the figure showing the process marker model in Trochim et al. [16], and described in the Methods section above.

In order fully to explore this approach we collected considerable data in the timeline for each case study, some of which is not shown. In some cases it was useful to be able to continue showing developments along a track even after the next track had started. For example, it is possible that activity in the later track might stall and further activity might be necessary in the previous track. Also, as shown in the amlodipine example, developments might occur in a track such as the post-launch effectiveness studies that lead to a higher uptake and move the intervention towards becoming standard practice (as a first line treatment) well after the date at which it first appeared in the guidelines or policy track (as a third line treatment). However, the questions about the necessity of analysing actions along the continuing track once actions have started in the next track do raise interesting questions that need further exploration.

Furthermore, within our matrix, we would be able to include, if thought desirable in future studies, the markers for items in the lower, more detailed, levels of the process marker model such as ethics review. It would be quite likely that the whole of assessment of the time lags involved in an application for ethical approval in any of our research tracks, for example Phase II, would be completed before activity started in the next track.

We think that combing a matrix and calibration points is an important methodological advance, but it cannot be applied to the same extent across all the types of research. While all the case studies had the same basic framework and some parallels could usefully be drawn between them, the quantitative analysis highlights the limitations on how far a uniform approach can be applied. These limitations are described in the next section because they can help contribute towards increased understanding of the nature of time lags. However, these observations also point to the importance of considering the reasons for conducting an analysis of time lags. Where the aim is to attempt to measure overall lengths of time lags for a whole portfolio of research (for example at a national level) then it might be important to have consistent start points. Where, however, the focus is on identifying specific policy actions to reduce time lags on specific issues then a focus on a specific part of the overall timeline, for example the ethics approval process, could be useful and it might not be necessary to identify the start or the end of an overall timeline for all the examples included.

#### Building better understanding of time lags and the diverse elements that can be involved

In terms of working across fields and interventions, our observations suggested that differences in patterns of time lags, and the case studies necessary to study them, might relate to different types of research in the different fields. The more complex therapies, such as “early intervention”, had many, often quite small, RCTs in contrast to other areas; this tended to be more a feature of mental health research than CVD research. Because of these differences, in some of these cases the full quantitative analysis across a series of tracks could not be applied (Table 3). Furthermore, there was evidence that for more complex interventions, the time to develop the expertise for implementation is important. The breadth of the framework we used and the flexibility we adopted in its application allowed understanding of these issues to emerge.

While it was difficult to be precise about all aspects of time lags, we were able to make observations about how far some time lags might, or might not, have been reduced without detriment to the goals of the health and innovation system. The desirable parts of the overall time lags include the many steps required in the translation of research to ensure safety, efficacy, and cost-effectiveness. This does not only apply to pharmaceutical products. For example, in the case of AAA screening some large aneurysms might be detected that would not have ruptured and, therefore, some men will be subjected to serious surgical procedure with associated risks to prevent ruptures that would not have occurred. Therefore, it was important to take the time necessary to conduct a large-scale rigorous trial to determine whether there would be an overall health gain from the introduction of AAA screening.

We also identified some undesirable delays within the case studies, and while generalizable patterns were difficult to establish, there seem to be more such delays in the discovery track. Examples include delays in the discovery track of the amlodipine timeline and in both CBT timelines.

### Identifying factors that speed up the translation process and options for addressing key policy concerns

As noted, it is dangerous to make generalisations from just seven cases, but some interesting and potentially useful observations about factors that might speed up the translation process, and areas where it is hoped action could be taken, do begin to emerge.

In the literature review it was noted that Mestre-Ferrandiz et al. [17] cited a study by Adams and Brantner [19], who observed that the development of ‘*drugs for HIV/AIDS have had the shortest Phase III and overall durations*’. In this case it is clear how the regulatory policy may affect the time lag ‘*as sponsors have been allowed to file NDAs* [new drug applications to the US Food and Drug Administration] *for almost all AIDS drugs without completing large-scale human clinical trials*’. Here, the process seems to have been speeded up by the adoption by the regulator of a different benefit-risk profile in response to the particular circumstances posed by HIV/AIDS and the demands of patients.

A range of suggestions from the documentary review are highlighted in Figure 2, where the matrix developed to assist the conduct of the case studies proved to be a valuable tool to present key elements from the review’s findings. These are set out more fully in Additional file 2. A limitation of the study is that the discovery track covers a wide range of activities and that disaggregation into separate tracks might allow more meaningful analysis. It should be noted, however, that during the study we were unable to come up with a clean and consistent way to carry out such disaggregation.

We identified various examples of factors that speeded up the translation process from our seven individual case studies. For amlodipine there were two important steps with the ASCOT trial [46] that speeded up translation. First, once the trial was stopped early because of the significantly higher mortality for patients in the other arm of the trial, the researchers completed the analysis and secured publication rapidly. [It should be noted that our analysis of this example was only possible because of the collection of data, such as the end-date for the trial, which was included in the list of markers from Trochim et al. [16], but was not included in our slimmed down list of calibration points, thus highlighting the need for flexibility]. Second, NICE announced it was going to speed up the revision of its guideline in the light of the anticipated results from the ASCOT trial, and applied the policy that allowed it to reduce the time between guideline revisions in the light of strong new evidence [47]. The latter example involved the policy system prioritising one therapy in particular circumstances. However, such initiatives could presumably only be used more widely if more resources were made available for bodies such as NICE. In the CBT cases, there were delays in implementation in the National Health Service because of the lack of trained therapists. Attempts to speed the process were made through investments in training more CBT practitioners through the Improving Access to Psychological Therapies initiative. Thus, additional resources were important once again.

Different patterns of the time elapsed in different case studies seem to reflect the different drivers of the process. With the pharmaceutical examples, especially once the process got as far as the human research tracks, there was considerable commercial pressure to move quickly. For example, when the company knew that Phase III trials for olanzapine were successful, it developed and implemented a global strategy for regulatory submission and approval aimed to speed up the launch in the market. There were probably fewer such direct drivers in the non-pharmaceutical cases, although the extensive work of one research team in the AAA case helped push the research along and the research team also played a part in driving some elements of the policy and implementation process as rapidly as possible. Some of the activities of the MRC as funder of the research also contributed to the creation of a study with sufficient power to make a policy impact that facilitated comprehensive translation into practice. Perhaps with “early intervention” also there were champions to drive it forward.

The case studies also help us to highlight factors that need to be taken into account when considering possible policy options to reduce time lags. Previous changes in policy and in the climate of research-funding are likely to have had some impact on the nature of our findings. The present is not the same as the past. For example, NICE was created in April 1999 and did not start producing guidelines and technology appraisals until 1999/2000, meaning that some apparent delays in timelines in research findings informing policies might have been alleviated had NICE been in existence earlier. Further, our focus on a range of different tracks helps emphasise that the causes of time lags, and the appropriate policy responses, might well differ in different parts of the overall timeline. Some of our analysis may demonstrate that “queues” play an important role in some time lags, i.e., time lags may result at least in part from one or a series of queues for resources, e.g., to conduct research or to obtain regulatory approval. Furthermore, while with hindsight there may appear to have been undesirable delays in the translation of what proved to be an effective intervention, at the time it was not necessarily clear that the particular line of discovery research, or particular intervention, should be given priority over others in the queue. Finally, while the diversity in the cases meant that various approaches were explored, it further limited the ability to make comparisons between different case studies in a way that might be used to inform policy. The heterogeneity across the seven case studies suggests a larger number of such cases might need to be developed to make robust policy observations, and that if only a few further studies could be conducted, then the most realistic approach would be to compare similar interventions, for example case studies of pharmaceuticals.

### Recommendations for further work

We first make some general recommendations about clarifying terminology, refining the matrix and considering the implications of the potential increase in availability of data. Then we discuss three ways in which research in this area could be further developed and applied to improve understanding of the time taken to move from invention to health and other benefits. Future studies to take the stream of work further will require careful planning in terms of objectives of the studies and the resources available to conduct them.

#### Clarifying terminology

Many components of overall time lags are necessary or desirable steps to ensure safety, efficacy, and effectiveness. Hence, we concluded that it will be preferable in future studies to use the generic term “time elapsed” to describe the overall time, and reserve the phrase “time lags” to describe the undesirable delays that might arise and that might be reduced if appropriate policies can be identified and introduced. We also debated the best term to use to describe the stages or phases of the matrix, and decided “tracks” had the advantage over other potential terms, such as streams, because it did not imply automatic movement in one-direction only. We recommend that this term be retained in future studies.

#### Further work to develop the matrix

A key area in which the matrix could be further refined is the track for discovery research. Analysis of this track would be easier if there was the development of a more disaggregated approach. The motivation for the research could be a key factor determining how the resulting disaggregated tracks are defined.

#### Exploiting the increasing availability of data

One common problem facing all of the proposals set out below, though in differing ways, would be how to find the appropriate balance between conducting a sufficient number of case studies to be able to make some generalisations, and drilling down in sufficient detail in each one to gather meaningful data. There are, however, various recently established sources of routine data that it might be possible to exploit in future. There will be an increasing availability of relevant data from sources. These include *Researchfish*, which is being used in the UK by the MRC and other research funders [48] (and the earlier e-Val and NIHR Awards Assessment Tool data collection), and also the increasing demands from journals for key dates in research studies to be included in publications. In principle, such sources could be drawn upon in any of the proposals for further studies set out below, but it would be important to explore how far stakeholders, for example the pharmaceutical industry, might already have access to some relevant data, and whether they would be willing to make the data available for the proposed analyses.

#### Areas for further possible work

Below, we set out three possible areas for future work that were informed by our analysis and consultation with stakeholders. These studies could probably be conducted in any country and could adopt a similar approach of using the international evidence for the research tracks and domestic material for the implementation/policy tracks.

##### Further work to understand the overall pattern of elapsed time and analyse areas of greatest undesirable delays or lags

At the seminar we ran there was clear support for an extension of the current case studies to a much broader set, provided a consortium of funders could be mobilised. Such case studies would apply the matrix and as full a set of calibration points as possible, and would also provide an opportunity further to validate the multi-track matrix.

##### Studies using data mining and bibliometric approaches

This proposal relates to a long-term ambition of this type of work to be able to assess the elapsed time for a large number of interventions in order to understand the characteristics associated with “fast” or “slow” translation. To assess this number of interventions would require an automated – and hence lower cost – approach. Currently, there are interesting advances in data-mining techniques employing bibliometric data that may be worth further exploration. For example, topic models take a statistical approach to looking at large corpuses of textual information. Suites of algorithms have been developed for discovering the main scientific themes in the journal *Science* between 1880 and 2002 [49]. If such approaches could be adapted to capture time information then it may be possible to automatically extract some calibration points from the literature using the advanced data mining techniques. If overall variations were identified through such studies this might lead to some policy responses to boost the factors associated with faster translation and to tackle obstacles associated with slow translation.

##### A focus on specific small sections of the matrix

Somewhat in line with the thinking and practice of Trochim et al. [16], there might be scope for studies that accept the overall matrix concept but focus on a series of detailed studies of a particular small part of the overall matrix. The aim here would be to collate data on a reasonably large number of cases but on a highly specific issue so as to identify the patterns of elapsed time involved in a specific segment of the timeline. This could be repeated in a number of small segments where there might be undesirable delays that could be addressed by specific policies or actions, e.g., to speed up the ethics review process, and shave time off the processes. If the time could be reduced, even by small amounts, in each of these steps, the overall time saved could be important. This type of approach would be of value to all stakeholders, but perhaps especially to the pharmaceutical industry, and perhaps the industry could therefore collectively be encouraged to collate the data from a number of their previous medicine development timelines. The proposal suggested here might provide a framework in which the existing data could be exploited more fully, and gaps in the data could be identified and efforts concentrated on collecting the additional data that would be of most value.

## Conclusions

We have made methodological advances in conceptualising how best to present analyses of elapsed time. The development and use of the multi-track matrix, as opposed to the single-track linear model, allows the data to be organised and presented in a way that supports analysis and understanding of time lags, provided it is used in conjunction with a narrative account interpreting key aspects and highlighting issues that are difficult to display. In particular, it has been important to demonstrate ways of measuring elements of the overall elapsed time by considering the time spent in each track before the cross-over to the start of activity in the next track. These advances provide a firm basis for further methodological work.

Overall, our approach has enabled us to demonstrate that the nature of elapsed time is often complex. While the total elapsed time is lengthy in all cases, and the start point and end point taken have a major impact on the total time identified, in some cases specific elements have been shown on the matrix, and reported in the quantitative analysis, as having been conducted rapidly. For example, the research phases in the amlodipine case were pushed through quickly by the company, and Phase III of the AAA screening case study started before Phase II had been completed or the publication related to it had been published.

It has been possible to identify a range of factors that have speeded up the translation in particular cases. While it is difficult to make generalisations from seven cases, we supplemented the case study data with some analysis from the literature and documentary reviews. We were also able to identify factors that need to be taken into consideration when identifying how to use studies to inform policy discussions on these topics.

Demonstrating the complexities has been useful, but it has also been resource-intensive. Such resource-intensity constrains the analysis that can be undertaken in the future, but it also means that any future studies would be most efficient if they build on previous work. The differences between types of intervention (sometimes exacerbated by differences between research traditions and regulatory requirements in different medical fields) mean that while it will, in our view, be useful to apply the matrix to all future studies, it is important to retain flexibility in how it is applied.

Finally, we have emphasised the need to distinguish between elapsed time and undesirable delays: certain periods of time are necessary or desirable in the translation of research to ensure the safety, efficacy, and cost-effectiveness of treatments. Therefore, we propose the use of more appropriate language such as the term “elapsed time” to describe any period other than those which seem to have involved undesirable delays. We have also developed the term “track of activity” to apply in the matrix. We recommend that these terms be used in future studies, including ones in the various possible areas in which we identified scope for taking the work forward.

## Electronic supplementary material

Additional file 1: **Literature review on time lags in areas relevant to the private sector.** This file contains a full account of the brief literature review of areas relevant to the private sector that had not been included in the earlier review by Morris et al. [15]. (PDF 639 KB)

Additional file 2: **Review of policy documents to identify the time lags highlighted.** This file contains the full review of UK (and other key) policy documents that was conducted to identify perceived reasons for time lags and the policy measures proposed to address them. (PDF 389 KB)

Additional file 3: **Seven case studies of time lags between conducting medical research and its translation.** This file contains all seven case studies in full; each consisting of a brief narrative account, a timeline, a version of the matrix, and a reflection on methodological issues that arose. (PDF 3 MB)

## Abbreviations

AAA: Abdominal aortic aneurysm
CBT: Cognitive behavioural therapy
CVD: Cardiovascular disease
MRC: Medical Research Council
NICE: National Institute for Clinical Excellence (and later variations)
RCT: Randomised controlled trial.
