# Supplementary material for: How long does biomedical research take? Studying the time taken between biomedical and health research and its translation into products, policy, and practice
Source: Health Res Policy Syst. 2015 Jan 1;13:1. doi: 10.1186/1478-4505-13-1 (PMC4297458; doi:10.1186/1478-4505-13-1)
Supplement: Supplementary file 1 — Additional file 1: Literature review on time lags in areas relevant to the private sector. This file contains a full account of the brief literature review of areas relevant to the private sector that had not been included in the earlier review by Morris et al. [15]. (PDF 639 KB) [file 12961_2014_368_MOESM1_ESM.pdf]

Additional File 1 in: Hanney SR, Castle-Clarke S, Grant J, Guthrie S, Henshall C, Mestre-Ferrandiz J, Pistollato M, Pollitt A, Sussex J, Wooding S: **How long does biomedical research take? Studying the time taken between biomedical and health research and its translation into products, policy, and practice.** *Health Res Policy Syst* 2015;**13**:1.

Literature review on time lags in areas relevant to the private sector

Morris et al. (2011) review the literature describing and quantifying time lags in the health research translation process. The authors find that the current state of knowledge of time lags is of limited use because the existing studies are usually not comparable. Their recommendations are that studies on time lags should be based on the same model and that it is necessary to formalise a process to gather the data used to measure lags in translational research.

The objective of this literature review is to expand Morris et al. (2011) to cover areas relevant for the private sector in the time lag estimation. The ultimate purpose of the review is to help inform the case studies analysed in the project "Time lags in medical research: Advancing a case study approach for a better understanding" funded by the MRC Methodology Research Programme and conducted by researchers at the Health Economics Research Group, RAND Europe and the Office of Health Economics. Our findings are similar to those in Morris et al. in that the nine papers we identified do not measure time lags in a comparable way.

In the next section we describe in detail the search strategy used to identify the relevant articles to consider in this study. In section 2 we present our findings. Section 3 focuses on the start and end points used to calculate the time lag, as this represents a main issue in the project. Section 4 concludes comparing our findings to those in Morris et al. and discussing possible implications for the "Time lags in medical research" project.

1. Search strategy

The search strategy was adapted from O'Neill (2010) and conducted using Google Scholar, Web of Science, PubMed and EBSCO based on key words. The search terms were selected to focus on three topics: time lags; research; and private sector.

Potentially relevant publications were identified through a two-step bootstrapping approach. In the first step, we adopted the same key words used in Morris et al. (2011) to define "time lags", and the words suggested by experience to define "research" and "private sector". The following terms and combination of logic operators (in upper case) were used:

Key words	Motivation of the choice
**'valley of death' OR 'bench to bedside' OR 'translational research' OR 'commercialisation' OR 'time lag' OR 'time-lag' OR 'delay' OR 'time factors' OR 'publication bias'**	same key words as in Morris et al. (2011) to define time lags
**AND**	
**'research' OR 'development' OR 'R&D'**	to focus on research
**AND**	
**'medical device' OR 'health intervention' OR 'pharmaceutical' OR 'drug' OR 'diagnostic' OR 'medical technology'**	to focus on the private sector technologies

There were no restrictions on the publication year or the searched field (e.g. only terms contained in title/abstract). Google Scholar produced 1,190,000+ hits, Web of Science 6,986, PubMed 37,734 and EBSCO 1,314.

In the second step, the key words were adjusted to identify the more relevant hits based on title using the following criteria:

i. papers which do not appear to refer to time lags in medical research – disregarded (unless they were relevant for point iii. below)
ii. papers which only focus on the public sector and pre-date Morris et al. (2011) – disregarded
iii. papers which are cited by Mestre-Ferrandiz et al. (2012) (a major source document) – kept
iv. papers which do not relate specifically to the aims of this search, but which may relate to other aspects of the project (e.g. policies to address time lags in medical research) – kept
v. papers which may update the conceptualisation of translational research in Morris et al. (2011) – only if published from 2011 onwards – kept.

In particular, the following key words were dropped: 'valley of death', 'commercialisation ', 'delay', 'publication bias', 'R&D' and 'diagnostic'.

The remaining first 50 hits per database were considered based on whether the abstract (where available) clearly indicated the full paper contained information related to the object of the literature search. In total, 30 articles were identified as potentially relevant; however, five of these were excluded as already included in Morris et al. (2011).[1] The remaining 25 papers were examined entirely and nine articles were as a result included in this study.

2. Findings

We show a summary of the relevant statistics from the nine time lags empirical studies in Table 1. All the studies focus on drug R&D, making it easier to compare the methodologies used by the authors to estimate the lags. One article (Mansfield, 1998) considers lags in R&D of drugs and other medical products together, implying that the author's estimates may not be directly comparable with those of the other studies. Estimates in Cockburn and Henderson (1997) and in Toole (2012) are for both public and commercial research: lags are measured from the start of basic public research to the Food and Drug Administration (FDA) application or the market introduction of the drug. Therefore, although these two studies provide useful information for our purposes, they cannot be used to calculate the average lags within private research alone.

[1] The papers already considered by Morris et al. are: Contopoulos-Ioannidis et al. (2008), DiMasi et al. (1991), DiMasi et al. (2003), Mansfield (1991), Sternitzke (2010).

Table 1: Summary of studies of time lags in health research

Author(s)	*Context*	*Start of time lag*	*End of time lag*	*Time lag (years)*				*Dates*	*Country*	*Notes*
				Lower Range	*Median*	*Mean*	*Higher Range*			
Achilladelis and Antonakis (2001)	drugs	R&D beginning	commercialisation			8-10		1950-1989	USA	
Chandy et al. (2006)	drugs	filing of the patent	first launch in the world			9.47		1980-2000	USA	disaggregated statistics per therapeutic category are also provided
Cockburn and Henderson (1997)	drugs	date of key enabling discovery	date of market introduction	7	19	24.4	67	1965-1992	USA	
DiMasi and Grabowski (2007)	biopharmaceuticals	date of Phase I begin	date of regulatory decision			8.1		1990-2003	USA	disaggregated data per each development phase are also provided: Phase I, 19.5 months; Phase II, 29.3 months; Phase III, 32.9 months, Regulatory, 16 months
Grewal et al. (2008)	drugs	early preclinical tests with animals	commercialisation			10-12		candidates under development on December 31, 2002	worldwide	
Huang et al. (2010)	drugs	initial discovery	FDA approval			10-15		1987-2010	USA	
Mansfield (1998)	drugs and medical products	recent academic research finding	first commercial introduction			8.8<br>8.5		1975-1985<br>1986-1994	USA	
Rake (2012)	drugs	date of preclinical investigation	date of marketing approval			12.5		1974-2008	USA	disaggregated data are also provided: preclinical testing, 5 years; clinical testing, 6 years; marketing approval, 1.5 years
Toole (2012)	drugs	investment in public basic research	FDA application	17			24	1980-1997	USA	

The remaining six studies present similar but not perfectly comparable points in the R&D process. These will be discussed in detail in the next section. Assuming the mean time lag in these six articles had been computed using sufficiently homogeneous methodologies, the resulting average time lag for private research is 10.5 years.[2] This result is consistent with the values found in other works focusing on pharmaceutical R&D (see Mestre-Ferrandiz et al. 2012). The average time lag in Cockburn and Henderson (1997) and Toole (2012), who estimate the lag between the start of public basic research and commercialisation of a drug, is 22.5 years. This figure may not be particularly significant, as it is based on two studies only, but it is compatible with the findings of other studies analysing the time needed for research evidence to reach clinical practice (e.g. HERG, OHE, RAND Europe, 2008).

Mansfield (1998) compares the time lag for drugs and medical products commercialised between two different time periods: 1975 -1985 and 1986-1994. The author finds that the lag between the most recent academic research finding and the first commercial introduction is about four months shorter in the latter period. Although it seems reasonable that that drug development could have shortened because of technological advances, it is not clear if the findings in Mansfield (1998) are due to quicker translation of academic into private research or to shorter private development only.

Generally, the time lag estimates for the other six papers included in this review are consistent with those in comparable studies identified in Morris et al. (2011) (the comparable studies, which focus on drugs and use regulatory approval/launch as endpoint, are reported in Table 2). These studies estimate the time lag in private research of drugs and, similarly to the literature identified here, the high variability of the results depends on the milestones adopted (the next section discusses this in more detail).

Table 2: comparable studies in Morris et al. (2011)

Author(s)	Start of time lag	End of time lag	Time lag (years)		
			Lower range	Mean	Higher range
**Cockburn and Henderson (1996)**	Date of enabling scientific research	Date to market	11	28	67
**DiMasi (1991)**	Clinical testing	Submission to FDA		6.3	
		Marketing approval		8.2	
**DiMasi (2003)**	Clinical testing	Submission to FDA		6	
		Marketing approval		7.5	
**Sternitzke (2010)**	Chemical synthesis	FDA approval		11.5	
**Wratschko (2009)**	Drug discovery	Commercialisation	10	12	17

[2] As in Morris et al. (2011), additional 'averaging' would be necessary to provide a single value for the mean time lag when the original article provided a range.

3. Milestones

Different studies use different start and end points to calculate the time lag. The choice of these mainly depends on the author's perspective and on the availability of information in the dataset used.

The points considered for the start of the time lag are: R&D beginning, initial discovery, filing of the patent, basic patent, preclinical tests, start of Phase I. Importantly, there can be a considerable time span between different start points, implying that the time lag estimation for pharmaceutical industry research can produce very different results according to the start point considered. For instance, Paul et al. (2010), cited in Mestre-Ferrandiz et al. (2012), find that the discovery stage (from initial discovery to preclinical testing) takes on average 4.5 years. Similarly, the time lag between preclinical studies beginning and start of clinical studies can take 3-4 years (Chandy et al., 2006). The time required to conduct preclinical studies would explain why DiMasi and Grabowski (2007), who only look at clinical development, estimate a time lag of 8.1 years while in Grewal et al. (2008) and Rake (2012) the time lag is approximately 12 years, who consider both preclinical and clinical development. One of the reasons why some authors prefer to consider preclinical and clinical start points, ignoring a significant part of the R&D needed to bring a drug to the market, is that there are several potential ways to trace the birth of a product idea (Chandy et al., 2006), so the definition of start point can be arbitrary and requires additional specification. Another reason why preclinical development is ignored is that it is often difficult to find drug-specific information in the preclinical stages (Mestre-Ferrandiz et al., 2012). Table 3 reports on the studies where the authors provide a description of the start point chosen.

Table 3: Time lag start points considered in different studies

Study	Start point	Description	Data source
**Chandy et al. (2006)**	Filing of the patent	Worldwide priority filing date associated with the primary patent	Data from Pharmaprojects, the Delphion database, and the FDA Orange Book
**Cockburn and Henderson (1997)**	Date of key enabling discovery	Drugs discovered through screening: date of first indication of activity in a screen. "Mechanism" based drugs: date of first clear description of the mechanism.<br><br>Third class: broadly indicative date	Case studies
**Mansfield (1998)**	Recent academic research finding	Academic research occurring within 15 years of the commercialization	Questionnaires and phone calls
**Toole (2012)**	Investment in public basic research	Fiscal year of award of extramural biomedical research grant and contract by the NIH and other	Extract from the NIH IMPAC database covering the years 1955–1994<br><br>NIH CRISP (Computer Retrieval of

	governmental agencies	Information on Scientific Projects) database covering the years 1972–1996

It emerges that start points may need to specify a geographical context, for instance the region where the patent was applicable (Chandy et al., 2006), or how to observe public investments (Toole, 2012). Moreover, trying to define the date of the key enabling discovery requires different specification according to the drug generation, and in third generation drugs only a broadly indicative date can be considered, as research is based on older classes of drugs (Cockburn and Henderson, 1997).

Generally, the definition of preclinical and clinical milestones is consistent with the definitions used by the Centre for Medicines Research International (CMRI), as reported in Mestre-Ferrandiz et al. (2012) and illustrated in Table 4. See Mestre-Ferrandiz et al. (2012) for a detailed discussion on how CMRI intervals compare with the more standard Phase I-III trials.

Table 4: Milestones definition adopted by the CMRI

Milestone	Definition
**First toxicity dose**	First dose given in the first animal toxicity study required to support administration to a human
**First human dose**	Dose administered for the first time to a human in a country
**First patient dose**	Active substance for the relevant project administered to patients for a specific indication with the intention of treating for that indication
**First pivotal dose**	First dose given to the first patient in the first pivotal safety and efficacy trial
**First submission**	First-ever regulatory dossier submitted to apply for a licence to market the compound for the project
**First launch**	The product is marketed for the first time

Source: Mestre-Ferrandiz et al. (2012).

In particular, the definition of “first toxicity dose” is consistent with the start points used in Grewal et al. (2008) and Rake (2012). The definition of “first human dose” is consistent with the start points used in Achilladelis and Antonakis (2001), DiMasi (2001, 2003), and DiMasi and Grabowski (2007).

There is more homogeneity in the choice of the end points, which usually refer to the licensing process, which are easier to observe.

Notably, almost all the studies are focused on drugs in the US market. This does not mean that the full drug development programme was conducted in the US but that relevant points in the measurement of the lag (regulatory application, marketing approval, launch) are referred to the US. This reflects the US market being the most important for drug commercialisation. When the end point explicitly denotes the marketing application (e.g. FDA application) or approval (e.g. date of marketing decision, FDA approval) a detailed description is not provided, as it is assumed to be immediately clear. However, in some cases (DiMasi and Grabowski, 2007; Rake, 2012) the authors

do not specify which marketing authority awards the licence, and assume implicitly that the readers will understand that the focus is on the US market. In other cases, summarised in Table 5 below, the authors make use of more ambiguous terms to define the end point but their definitions appear broadly consistent with that used by the CMRI.

Table 5: Time lag end points in different studies

Study	End point	Description	Data source
**Chandy et al. (2006)**	First launch in the world	Date of drug approval (*pages 25 and 28*)	data from Pharmaprojects, the Delphion database, and the FDA Orange Book
**Cockburn and Henderson (1997)**	Date of market introduction	Not available, but apparently coinciding with regulatory approval (*page 44*)	Case studies
**Grewal et al. (2008)**	Commercialisation	Commercialisation approval (*page 7*)	Pharmaprojects database

Chandy et al. (2006) refer to drug launch throughout the main part of their paper and only in the appendix do they describe the end point as the date of drug approval. Cockburn and Henderson (1997) consider the date of market introduction, but do not clarify whether this refers to the date when it is possible to market the drug (regulatory approval) or to the date when the drug is actually made available in the market. Grewal et al. (2008) use the vague term 'commercialisation' and in one point of the paper they specify that this coincides with commercial approval (i.e. regulatory approval).

As shown in Figure 1, the choice of the end point is important to provide a consistent measure of the time lag, as sometimes 1-2 years can pass between submission to the regulatory body and being awarded marketing approval. In addition, if the time of the actual launch were considered (i.e. when the drug is first made available), this would imply an additional lag ranging between a few months and more than one year depending on the market of reference. This is because in some countries there is an additional process to determine the pricing and reimbursement of new medicines, which takes place immediately after regulatory approval but which is necessary for the drug to be available for use in the market. In Europe, for instance, a study conducted by the European Federation of Pharmaceutical Industries and Associations (EFPIA) measured the number of days elapsing from the date of EU marketing authorisation to the day of completion of post-marketing authorisation administrative processes (including pricing and reimbursement processes). The study found that the average time between marketing authorisation and patient access in 11 European countries varies from 88 to 392 days (not considering Germany and the UK), as shown in Figure 2 (EFPIA, 2010).

Figure 1: Time elapsed between first world application in any market and launch (selected countries)

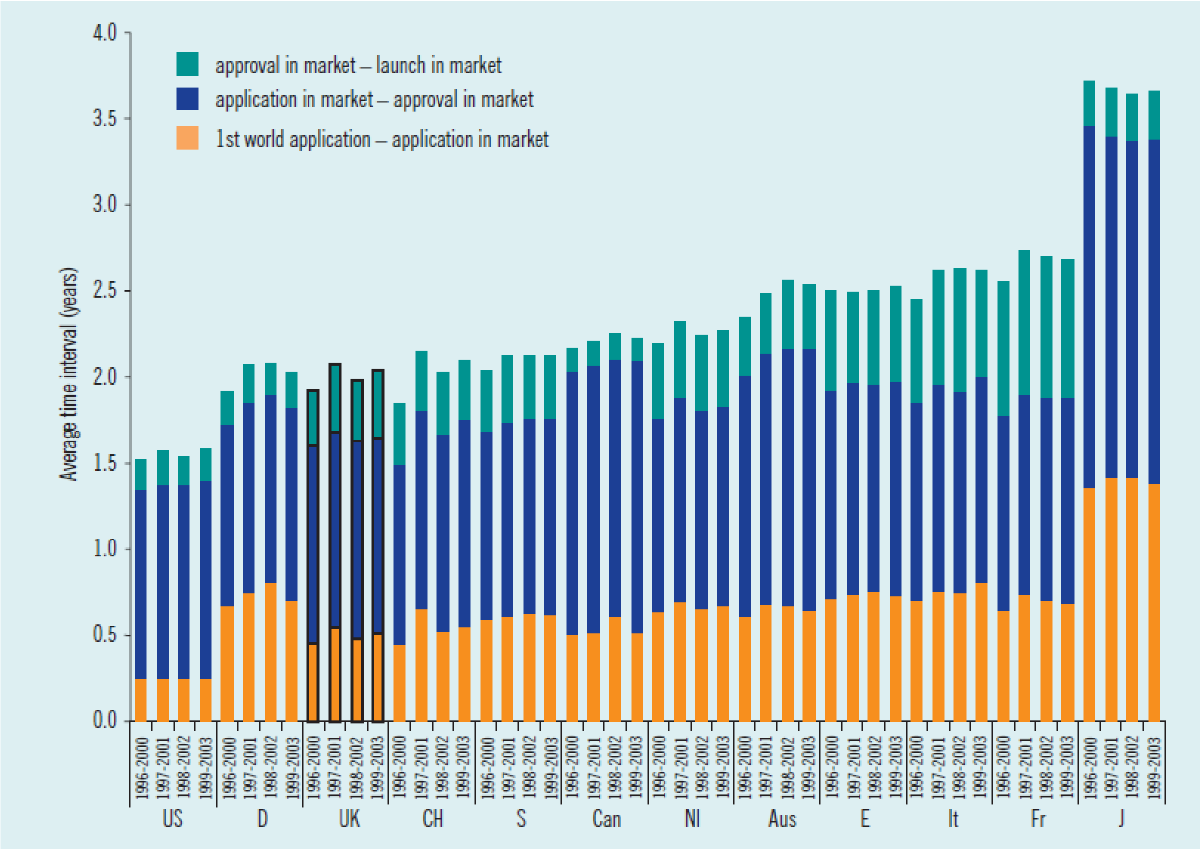

Source: PICTF (2004).

Figure 2: EFPIA patients' W.A.I.T. indicator

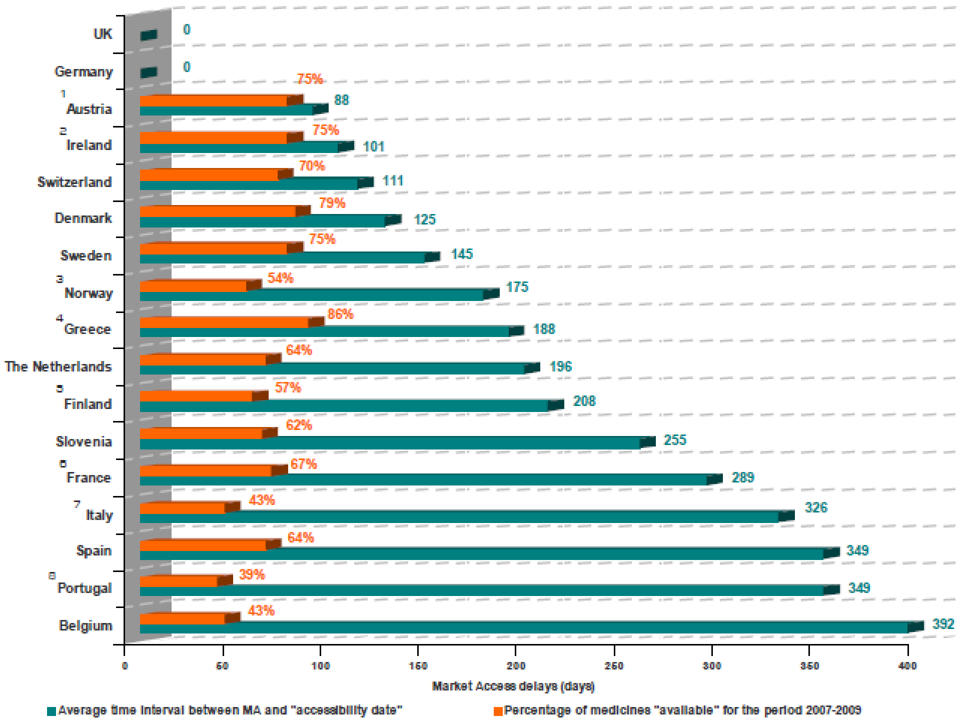

Source:EFPIA (2010).

4. Discussion

Morris et al. (2011) find that the average time lag in medical research is 17 years. However, the authors observe several issues about the quality of the existing studies:

- data are generally sparse and estimates vary;
- measurement and reporting is often poor;
- some studies aggregate data from earlier studies without critical reflection or recognition of this.

In our review we have encountered similar issues but these were not discussed in detail as they are beyond the objective of our analysis.

The more important issue described in Morris et al. is the inconsistency in the definition of start and end points to estimate the lag, making meaningful comparisons across different studies difficult. We observed the same problem in the studies we analysed about the private sector R&D process. Although all the articles study time lags in drug development, there is no general agreement on which start point in the process to consider. In general, it seems that authors choose the start points that are easier to observe and that are collected in publically available databases. By contrast, the choice of the end point seems to be uncontroversial and all but one study consider marketing approval as the end point. Nevertheless, the adoption of very different start points implies that time lag estimates vary considerably across studies. In Table 6, we report the estimated time lag (expressed in years) for the nine studies we identified here and the five comparable studies in Morris et al. (2011). In particular, as shown in Table 6, the highest variability occurs when start points related to academic/public research are considered, as these may be defined in several, different ways.

Table 6: Time lags in drug R&D considering different start milestones

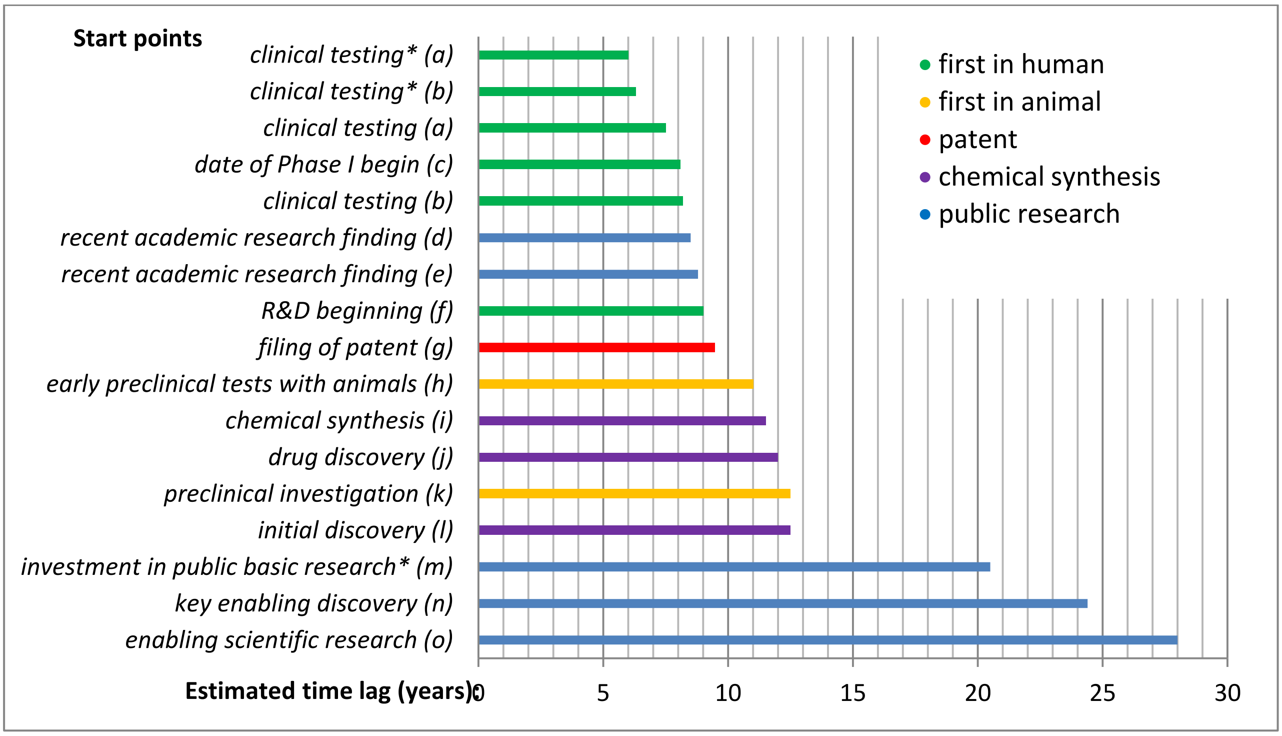

**End of the time lag represented by regulatory submission (rather than approval).*
Studies: (a) DiMasi (2003); (b) DiMasi (2001); (c) DiMasi and Grabowski (2007); (d) Mansfield (1998), years 1986-1994; (e) Mansfield (1998), years 1975-1985; (f) Achilladelis and Antonakis (2001); (g) Chandy et al. (2006); (h) Grewal et al. (2008); (i) Sternitzke (2010); (j) Wratschko (2009); (k) Rake (2012); (l) Huang et al. (2010); (m) Toole (2012); (n) Cockburn and Henderson (1997); (o) Cockburn and Henderson (1996).

The variability of the results in time lag studies may not depend on methodological issues alone but may also be related to other factors, i.e. the type of medical research and the therapeutic area. Since our search produced nine articles mainly focusing on private drug development, it is easier for us to compare different studies and to identify some of the drivers of the "intrinsic" variability in time lags. DiMasi and Grabowski (2007) find that *"total clinical plus approval time is 8% longer for biopharmaceuticals [vs. chemical drugs], with nearly all the difference accounted for by phase I"*. This suggests that time lags for biological and chemical drugs might be estimated separately and policies to reduce the development time might be designed ad hoc. Chandy et al. (2006) find that the mean time between patent filing and launch varies considerably according to the therapeutic area treated by a drug. The mean time ranges from 8.54 years for anti-infective medicines to 15.25 for immunological drugs. This result may be due to scientific barriers to technical development in a particular therapeutic area and also to specific regulatory policies to favour the research in areas of great unmet need. Mestre-Ferrandinz et al. (2012), in their review, also show different durations across different therapeutic areas. For instance, in the US, the FDA has implemented three methods to speed the development and availability of drugs that treat some serious diseases, especially when the drugs are the first available treatment or have advantages over existing treatments: fast track, accelerated approval, and priority review.[3] Table 7 provides further details about these approaches.

Table 7: FDA approaches to accelerated drug development and approval

Approach	Objective(s)	Benefits from the designation to the approach
**Fast track**	To facilitate the development, and expedite the review of drugs to treat serious diseases and fill an unmet medical need	• More frequent meetings with FDA to discuss the drug's development plan and ensure collection of appropriate data needed to support drug approval<br>• More frequent written correspondence from FDA about such things as the design of the proposed clinical trials<br>• Eligibility for Accelerated Approval, i.e., approval on an effect on a surrogate, or substitute endpoint reasonably likely to predict clinical benefit<br>• Rolling Review, which means that a drug company can submit completed sections of its New Drug Application (NDA) for review by FDA, rather than waiting until every section of the application is completed before the entire application can be reviewed. NDA review usually does not begin until the drug company has submitted the entire application to the FDA
**Accelerated approval**	Earlier approval of drugs to treat serious diseases	Approval of a drug based on surrogated endpoints (i.e. laboratory measurements used as an indirect or substitute measurement of clinically meaningful outcomes, such as survival or symptom improvement, which could take many years to be observed)
**Priority**	Create a two-tiered	The time it takes FDA to review a new drug application is reduced to

[3] Information available of the FDA website: http://www.fda.gov/forconsumers/byaudience/forpatientadvocates/speedingaccesstoimportantnewtherapies/ucm128291.htm [accessed on 14th April 2013].

**review**	system of review times to prioritise the approval of drugs that offer major advances in treatment, or provide a treatment where no adequate therapy exists	approximately 6 months (vs. 12+ months needed for standard review).

Source: FDA website.

In addition to these approaches, the FDA dedicates further specific resources to cancer and HIV drugs. For instance, an expanded access mechanism is designed to make promising products, which have not yet cleared the FDA approval process, available as early in the drug evaluation process as possible to patients without alternative therapeutic options. This has contributed to accelerating clinical development: *"drugs for HIV/AIDS have had the shortest Phase III and overall durations"* (Mestre-Ferrandiz et al., 2012) because *"sponsors have been allowed to file NDAs for almost all AIDS drugs without completing large-scale human clinical trials"* (Adams and Brantner, 2006). The fact that regulatory agencies prioritise some therapeutic areas implies that different studies using the same time points to estimate the development lag of drugs may produce very different results depending on the set of medicines analysed.

To conclude, we summarise some of the points that may help inform the case studies to be studied in the "Time lags in medical research project":

- the drug regulatory environment is considerably different across countries and this can imply different estimates for the time lag between different countries (although for our purposes, the European regulatory environment can be deemed as relatively homogenous given the existence of the EU centralised approach via the EMA);
- the choice of the initial point is very heterogeneous and unfortunately many studies do not explain the choice of a particular starting point. In general, start points related to events widely tracked in publically available databases tend to be preferred;
- almost all studies adopt the same end point (marketing authorisation). The reason is probably because data about drug licensing are easier to retrieve although this approach ignores a time lag that exists between the authorisation and the actual launch and/or uptake – the so-called 'pricing and reimbursement' delay (which also differs across countries);
- when public research is considered as a start point, the estimates of the time lag appear to have greater variance. This can be related to the fact that the definition of the initial point for public research is possibly more difficult to define than the definition of a start point for private research, especially if public research focuses on pre-clinical stages (and sometimes such public research might not be drug-specific);
- all the studies focus on one therapeutic indication only (the first one the drug is approved for) and do not consider if the same drug is also marketed later for further indications.

However, it is worth pointing out that these considerations might be specific to the case studies analysing drugs only and might not apply to medical research in general.

References

Achilladelis, B., and Antonakis, N. (2001). The dynamics of technological innovation: the case of the pharmaceutical industry. *Research Policy*, 30(4), 535-588.

Adams, C. P., and Brantner, V. V. (2006). Estimating the cost of new drug development: is it really $802 million? *Health Affairs*, 25(2), 420-428.

Chandy, R., Hopstaken, B., Narasimhan, O., and Prabhu, J. (2006). From invention to innovation: Conversion ability in product development. *Journal of Marketing Research*, 494-508.

Cockburn, I., and Henderson, R. (1996). *Public–private interaction in pharmaceutical research.* Proc. Natl. Acad. Sci. 93:12725-12730.

Cockburn, I., and Henderson, R. (1999). *Public-private interaction and the productivity of pharmaceutical research (No. w6018)*. National Bureau of Economic Research.

Contopoulos-Ioannidis, D. G., Alexiou, G. A., Gouvias, T. C., and Ioannidis, J. P. (2008). Life cycle of translational research for medical interventions. *Science*, 321(5894), 1298-1299.

DiMasi, J. A., and Grabowski, H. G. (2007). The cost of biopharmaceutical R&D: Is biotech different? *Managerial and Decision Economics*, 28(4-5), 469-479.

DiMasi, J. A., Hansen, R. W., and Grabowski, H. G. (2003). The price of innovation: new estimates of drug development costs. *Journal of health economics*, 22(2), 151-186.

DiMasi, J. A., Hansen, R. W., Grabowski, H. G., and Lasagna, L. (1991). Cost of innovation in the pharmaceutical industry. *Journal of health economics*, 10(2), 107-142.

European Federation of Pharmaceutical Industries and Associations (EFPIA) (2010). Patients W.A.I.T. Indicator. Retrived 19 April 2013 from: http://www.efpia.eu/patients-wait-indicator-report-2010

Grewal, R., Chakravarty, A., Ding, M., and Liechty, J. (2008). Counting Chickens before the Eggs Hatch: On the Valuation of New Drug (Product) Development Portfolios in the Pharmaceutical Sector.

Health Economics Research Group, Office of Health Economics, RAND Europe (2008). *Medical Research: What's it Worth? Estimating the Economic Benefits from Medical Research in the UK*. London: UK Evaluation Forum.

Huang, M. C., Fang, S. C., and Chang, S. C. (2011). Tracking R&D behavior: bibliometric analysis of drug patents in the Orange Book. *Scientometrics*, 88(3), 805-818.

Mansfield, E. (1991). Academic research and industrial innovation. *Research policy*, 20(1), 1-12.

Mansfield, E. (1997). Academic research and industrial innovation: An update of empirical findings. *Research Policy*, 26(7), 773-776.

Mestre-Ferrandiz J., Sussex J., and Towse A. (2012). *The R&D cost of a new medicine*. London: Office of Health Economics.

Morris, Z. S., Wooding, S., Grant, J. (2011). The answer is 17 years, what is the question: understanding time lags in translational research. *Journal of the Royal Society Medicine*, 105, 510-520.

O'Neill, P. (2010). *Shedding the Pounds: Obesity Management, NICE Guidance and Bariatric Surgery in England.* London: Office of Health Economics.

Paul, S. M., Mytelka, D. S., Dunwiddie, C. T., Persinger, C. C., Munos, B. H., Lindborg, S. R., and Schacht, A. L. (2010). How to improve R&D productivity: the pharmaceutical industry's grand challenge. *Nature Reviews Drug Discovery*, 9(3), 203-214.

Pharmaceutical Industry Competitiveness Task Force (PICTF, 2004). *Competitiveness and Performanace Indicators 2004*. Retrived 18 February 2013, from: http://www.dh.gov.uk/en/Publicationsandstatistics/Publications/PublicationsPolicyAndGuidance/DH_090487

Rake, B. (2012). Determinants of Pharmaceutical Innovation: The Role of Technological Opportunities Revisited. *Jena Economic Research Papers*, 2012, 018.

Sternitzke, C. (2010). Knowledge sources, patent protection, and commercialization of pharmaceutical innovations. *Research Policy*, 39(6), 810-821.

Toole, A. A. (2012). The impact of public basic research on industrial innovation: Evidence from the pharmaceutical industry. *Research Policy*, 41(1), 1-12.

Wratschko, K. (2009). *Empirical Setting: The pharmaceutical industry. Strategic Orientation and Alliance Portfolio Configuration*. New York, NY: Springer.
