# Supplementary material for: How long does biomedical research take? Studying the time taken between biomedical and health research and its translation into products, policy, and practice
Source: Health Res Policy Syst. 2015 Jan 1;13:1. doi: 10.1186/1478-4505-13-1 (PMC4297458; doi:10.1186/1478-4505-13-1)
Supplement: Supplementary file 2 — Additional file 2: Review of policy documents to identify the time lags highlighted. This file contains the full review of UK (and other key) policy documents that was conducted to identify perceived reasons for time lags and the policy measures proposed to address them. (PDF 389 KB) [file 12961_2014_368_MOESM2_ESM.pdf]

Additional file 2: Review of policy documents to identify the time lags highlighted. *Health Res Policy Syst 2015;* **13:**1

Author	Title	Year	Time lag	Reasons for time lags	Measures to address	Quotes from document	Notes
BIGT	Bioscience Innovation and Growth Team (BIGT) report	2003		Lengthy drug approval times	New draft laws revising the legal framework governing approval of medicines in Europe (revisions to Directive 2001/83/EC (codification) and Regulation 2309/93/EEC (the EMEA centralised procedure)) are currently (as of publication date) awaiting a common position from the Council before a second reading in the European Parliament. These proposals include three areas of particular interest to the BIGT:<br>• An accelerated assessment procedure for approval of drugs "which are of major interest from the point of view of public health and in particular from the viewpoint of therapeutic innovation";<br>• Conditional marketing authorisation for breakthrough medicines, subject to agreed clinical or pre-clinical testing; and<br>• Harmonisation of compassionate use procedures.	Lengthy drug approval times, which consume vital years of patent protection and delay arrival of drugs on the market. As Table 2.1 indicates, drug approval times remain long for the EMEA centralised procedure, the process through which biotech drugs are approved – and longer than approval through the FDA. In the US, moreover, FDA Commissioner Mark McClellan announced at the BIO2003 Convention in June 2003 that the FDA was due to update its regulations with the aim of reducing drug approval times by 10% or more. No such commitment has been made by European regulators, despite the fact that they are starting from a higher base in terms of drug approval times. (p.52)	
				How quickly drugs can be effectively marketed following approval. NICE often seen as a hurdle, particularly for niche drugs for small numbers of patients	Recommends that industry should work with NICE, MHRA and EMEA to improve transparency in order that:<br>• NICE is aware of clinical trials data at the earliest appropriate moment for each individual company. NICE and industry should engage in mutual education – about, for example, which drugs NICE will look at, NICE criteria for niche medicines/therapies, and the best framework for evaluating total cost of care.<br>• MHRA, EMEA and industry engage early in the development process to discuss which patient outcomes will be important for subsequent acceptance (along the lines of FDA-industry interaction).<br>In addition:<br>• NICE should take full account of the wider economics of health and social care when making decisions about the cost-effectiveness of therapies.<br>• MHRA and EMEA should seek to at least match the FDA's target of reducing drug approval times by 10%.<br>• The FDA, EMEA and MHRA should also be encouraged to work closely together to ensure shared process and protocols.		
NAO	Getting the Evidence: Using research in policymaking	2003				The procurement of research by departments presents research managers with a number of challenges. First, there will be uncertainty about the value and the outcome of research. As a result there is always an inherent risk in procuring research. Second, **there is often a long time interval between funding the research and its impact on policy becoming evident**. The combination of these two challenges makes it even more important that those responsible for research and development in departments can demonstrate the need for and utility of high quality, relevant, research in support of departmental missions, aims and objectives.	
				Time taken to review proposals		Researchers had relatively high levels of dissatisfaction about the time taken for the department to make a decision on proposals, although there was significant interdepartmental variation, with 53 per cent of respondents being very or quite dissatisfied for the Department for Environment, Food and Rural Affairs, compared to 11 per cent for the Office of the Deputy Prime Minister.	
AMS	Strengthening Clinical Research (Bell Report)	2003		Fragmented trial capacity, long-start up times, low recruitment rates, high/variable costs, regulatory constraints, 'less welcoming culture'		Companies have expressed concerns over the last decade about the decline in the UK as an attractive location for clinical trials, specifying fragmented research trial capacity, long start-up times, low patient recruitment rates, high and variable costs as well as regulatory constraints and a less welcoming culture than other countries (Poste, 2001)	
NIH	NIH Roadmap	2004			Re-engineering the Clinical Research Enterprise. This initiative is intended to develop networks of academic centres which are linked to health care providers with large patient groups, and which will be involved in quickly developing, testing and delivering new interventions.		(Highlighted in Cooksey, 2006)

Additional file 2: Review of policy documents to identify the time lags highlighted. *Health Res Policy Syst 2015;* **13:**1

Author	Title	Year	Time lag	Reasons for time lags	Measures to address	Quotes from document	Notes
HM Treasury	Science and Innovation framework 2004-2014	2004	6 yrs to publication, 7 yrs to citation			Regional Development Agencies should have targets that promote business-university collaboration. Their core outcome target for innovation should reflect the long time lag between R&D and economic impact.	
						The Framework Programme is the EU's third biggest funding mechanism: the Sixth European Framework Programme, running from 2002-06, has a budget of €19 billion. However, the Programme currently lacks an effective system of performance measurement. Progress is being made to improve the monitoring and assessment of the programme's delivery processes, but there is a bigger challenge of tracking the longer- term impact of the programme as a whole. **It can take many years before projects have quantifiable outputs, and often the contribution of a project to a new product or service is lost due to the complex evolution of a project and the timescales involved. It is important, nonetheless, to increase efforts to perform strategic evaluations and assess the long-term impact of the programme on European science and technology.**	
						A recent study by the Science and Technology Policy Unit (SPRU), also commissioned by the OST, sheds more light on the way that public expenditure on science leads to outputs 20 . The combined impact of UK and other countries' spending on Higher Education R&D means that a 1 per cent increase in each of these sources of funding leads to a 1 per cent increase in publications and a 1.1 per cent increase in citations. **These effects do not happen immediately. It takes six years for the full effect to impact on papers and seven years for citations.**	See SPRU 2004 report below
					PSA aimed at improving UK's innovation performance internationally (nothing specifically on time lags though)	Managing science and innovation expenditure is, however, challenging due to the inherent uncertainty about the creative and experimental nature of the work, **and the long timescales over which performance becomes evident.** To meet this challenge DTI has designed a robust management system around the Public Service Agreement it has agreed with HM Treasury for the present 2004 Spending Review, namely to: Improve the relative international performance of the UK research base and increase the overall innovation performance of the UK economy making continued progress to 2008, including through effective knowledge transfer amongst universities, research institutions and business. (for detail of PSA see p163 of strategy doc - nothing specific on reducing time lags)	
SPRU (Crespi & Geuna)	The Productivity of Science	2004	6 yrs to publication, 7 yrs to citation			Analyses of scientific productivity or performance that do not include a time lag between investment and outputs will almost certainly be misleading. It takes six years for publications and seven years for citations before the full effect of an increase in Higher Education research and development spending is achieved. For the first two years there is no significant return from investment. In the case of publications, by the end of four years about 50% will have appeared, while for citations it is five years before this 50% threshold is reached.	

Author	Title	Year	Time lag	Reasons for time lags	Measures to address	Quotes from document	Notes
						Significantly different lag structures were identified for publications and citations showing that the science system does not respond uniformly to changes in the sources of funds. Medical Sciences showed a long lag before full returns from R&D spending were achieved while the results in Social Sciences can be seen within the first few years. The impact of an increased Science Budget is sequential starting with Social Sciences then being apparent in Engineering and Natural Sciences and finally appearing in Medical Sciences. Interestingly, when we considered the graduate student research output, Social Sciences is the only scientific field that requires a long period before full returns are achieved. It takes more than 5 year to obtain 90% of the impact for Social Sciences, while for the other three fields roughly 90% of the returns are achieved within 3 years.	
McKinsey	Clinical research in the UK	2005				Overall, the UK is not distinctive on any of the parameters that matter to industry. Indeed, while it is neither better nor worse than other comparator countries on the dimensions of strategic relevance and quality, it has longer trial start-up times, more recruitment delays, poorer reliability and higher costs.	
				Slow product uptake	Help industry accelerate product approval or uptake by co-presenting outcomes or health economics data to NICE and providers (e.g., NHS Foundation Trusts). This could take the form of an NHS stamp of approval for selected studies similar to the stamp being considered by the US National Institutes for Health in submissions to the FDA.	Strategic relevance. Here the UK is on a par with its competitors. The UK is the third largest pharmaceuticals market in Europe and the fifth largest in the world. **Market access is comparatively efficient, without the pre-launch pricing and reimbursement negotiation delay common in many other European markets. However uptake of new products is slow**.	
				Poor industry interface	An effective interface for industry could make R&D approval, site set-up, access to patients, and follow-up (e.g., Case Report Form completion and transmission) much more reliable, efficient and timely. This could turn what many stakeholders see as one of the greatest problems with the UK clinical research environment into a major strength. For example, a company conducting a clinical study in the UK could have one clear point of contact within the NHS accountable for all aspects of approval, set-up, project management and close-out.<br><br>Comprehensive and flexible healthcare IT system. Again, building on the asset of a single national healthcare provider, the UK could create the world's largest integrated patient record IT system.<br><br>Create a body to resolve disputes between industry and the NHS. For example, an NHS body could address issues such as excessive site set-up delays, missed recruitment targets, and poor Case Report Form quality.	Time. The UK is below average. Some hard evidence and anecdotes from interviews suggest that the UK is slower than other countries. Interviews further elucidate that poor industry interfaces increase trial and investigation set-up times and create unnecessary complexity.	
				Fragmented bureacracy	Concrete initiatives in operation to help bypass bureaucracy and improve trial execution. This should include: a single NHS-wide R&D sign-off; metrics on NHS Trusts' R&D performance (e.g., volume of clinical research activity, length of start-up time, 'overhead' cost), and a streamlined interface between industry and the NHS including the creation of a body to resolve disputes.	Fragmented bureaucracy and non-binding procedures make trial/investigation set-up complicated and cumbersome. There are often additional barriers to arranging trials in primary care and in secondary care. **Trust-level NHS R&D approval is not standardised and frequently creates delays. In practice, the separation of regulatory, ethical and R&D approval in the NHS results in duplication and frequent breaches of target approval time-lines.** In addition the Scottish and Welsh procedures are evolving differently from those in England.	

Author	Title	Year	Time lag	Reasons for time lags	Measures to address	Quotes from document	Notes
				Lack of access to physicians and expert regulators	Improving patients', physicians' and broader healthcare professionals' perceptions of research is key. Getting this right will help make these groups more willing to participate, leading to larger recruitment populations, faster start-up times, better performance against enrolment targets and lower drop-out rates. The NHS has the credibility to promote the benefits of clinical trials to all of these groups in ways that industry cannot.<br><br>Motivated and educated physicians and patients. Physicians and patients willing to participate in clinical research would give industry larger recruitment populations, faster start-up times and better performance on enrolment.	Biotech start-ups are very sensitive to trial time and cost. Their limited funding demands fast results to secure further R&D investment. For devices, the development process tends to be more iterative, often needing many sequential but relatively small clinical investigations with higher levels of investigator interaction. For this reason, accesses to high-quality physicians and superior guidance from expert regulators have tended to be the most important factors. Even so, larger device companies are now following the trend to do increasing amounts of research in emerging locations (e.g., India, China) for reasons of cost and time.	Highlights differences between sectors in importance of speed
					The transparency created by clear metrics is likely also to improve timeliness and reliability.		
Cooksey	A review of UK health research funding	2006		Pricing/reimbursement system	Immediate launch and reimbursement of a medicine following receipt of a marketing authorisation	It is also important to remember that the statistics on uptake following marketing authorisation may be distorted by the different pricing/reimbursement systems in different countries. **The UK is one of the few countries that will allow immediate launch and reimbursement of a medicine following receipt of a marketing authorisation, allowing less time for pre-launch preparations (e.g. marketing), whereas the launch of new medicines in other countries can be delayed by pricing discussions, allowing time for pre-launch preparations and therefore creating the conditions for faster uptake after launch.**	
				Adopting technology (uptake tends to accelerate rapidly once technology accepted). However, it does take a certain length of time to assess technologies in practice.	Particularly rapid uptake when covered by a National Service Framework. NHS should be looking to achieve fast uptake of cost-effective new technologies, not new technologies per se. The people, capacity and ability to carry out health research effectively are crucial to making this happen. National Innovation Centre, taking overall lead for IP management for technology innovations from the NHS, intended to speed up uptake.	The Review has seen early work prepared for the Ministerial Industry Strategy Group (MISG) for the pharmaceutical industry which suggests that rates of uptake of new medicines in the UK relative to other European countries vary hugely depending on the medicine, and **does not support the notion of the UK as a relatively 'slow uptaker' per se. It may be, however, that the UK is a relatively slow uptaker for more expensive medicines** — we would need to see more data to disprove or support this hypothesis.	
				Devolved structure of NHS presents problems for those wanting to work with the NHS, particularly running clinical trials or introducing new technologies			
				Complex regulation around healthcare product development<br><br>Health Technology Assessments happen too late in the drug development process	New drug development pathway, involving:<br>more rapid discrimination between potential new therapies at earlier stages of drug<br>development;<br>- earlier 'conditional licensing' of new drugs;<br>- involving NICE earlier in the process of development to accelerate assessment of<br>clinical and cost-effectiveness;<br>- faster uptake of cost-effective drugs;<br>- clearer processes for ensuring NICE initial assessments and recommendations for<br>further research are followed-up more systematically;<br>- the use of the NHS National Programme for IT (NPfIT) to ensure more rapid<br>assessment of any emerging side-effects and efficacy over longer periods;<br>- streamlining of processes involved in setting up and costing clinical trials; and<br>- the use of NPFIT to identify appropriate patients for clinical trials.	**the government must consider ways of bringing drugs that address UK health priorities to market faster, but without compromising patient safety**. It is increasingly clear that the current way of developing drugs in the private sector is unsustainable in the long-term. The Review found that regulations around the healthcare product development process have become ever more complex, and that **Health Technology Assessment arguably happens too late in the drug development process**. (p.6)	

Author	Title	Year	Time lag	Reasons for time lags	Measures to address	Quotes from document	Notes
ESF	EMRC White Paper: Present Status and Future Strategy for Medical Research in Europe	2006				There is a time-lag between research and tangible outcomes, and it is difficult to trace the role of individual research contributions.	
Sainsbury	The Race to the Top: A Review of Government's Science and Innovation Policies	2007	Ave 6-8 yrs for different technologies			The innovation cycle itself, requiring extended periods of product research and development, with frequent (and often relatively large) capital input, can create problems for financing. For example, small biotechnology firms, with typical product development cycles of 10–15 years, are particularly vulnerable.	
DH	Best research for best health	2007				The aims of the Clinical Research Network (CRN) for England are to… Improve the quality, speed and coordination of clinical research by removing the barriers to research in the NHS	
British Academy	Punching our weight: the humanities and social sciences in public policy making (Wilson Report)	2008				EvidenceNetwork offered social scientists the following advice on the ways in which they might be able to enhance the prospect of their research informing public policy...take the long view and stick at it - sometimes research impact just takes time.	
IOM	Evidence-Based Medicine and the Changing Nature of Healthcare: Meeting Summary	2008		Process guidelines		Moreover, process guidelines can slow innovation, because agreeing on guidelines is inevitably slow and invariably political. Medicine is constantly being refined, and guidelines can lag best practice or, conversely, lead to undue attention to processes that have yet to be definitively proven with a sufficient body of evidence.	
				Complexity of evaluating new technologies		The clear benefits of medical technologies have underscored the importance of supporting innovation in healthcare product development, and **perhaps most pressing in this respect is the development of new approaches to accelerate the evaluation of new technologies.** The complexity introduced by many new technologies has had two major consequences: (1) waste and inefficiency in the processes of evidence generation, due to poorly defined evidence needs with respect to coverage and reimbursement decisions; and (2) **delayed action on the potential of transformative technologies that might enable disruptive and positive changes in clinical care and administrative processes. Key challenges for health care are the development of approaches for the early identification of beneficial technologies,** as well as for generating the evidence needed to evaluate these emerging technologies.	
			"a decade or more"			The dissemination and incorporation of new knowledge into practice must be accelerated so that it does not require a decade or more for the average provider to adopt new knowledge and skills.	
				Time taken to build evidence base	Include evidence beyond RCTs	One of the major strategies proposed to hasten development of the required evidence base includes migration away from traditional reliance on RCTs and inclusion of a variety of other approaches and data sources. These include cluster analyses, registries with or without “coverage with evidence development” financing, and other innovative approaches. While such efforts may indeed supplement RCTs and accelerate the development of the needed evidence, there are concerns among many that such approaches do not have the reliability of the “gold standard” RCT, and that the time saved may not be worth the price paid in quality and reliability of the evidence.	
						Despite a widespread impression that the pace of new technology introduction is escalating (Kessler et al., 2004), the most common measure of it—Food and Drug Administration (FDA) approvals of new products—finds the pace steady and relatively even (FDA, 2006)	

Author	Title	Year	Time lag	Reasons for time lags	Measures to address	Quotes from document	Notes
				Increase in outcomes evidence required for drugs	The ability to stratify drug development using biomarkers and the tailoring approach offers potential for reducing costs and approval times.	Steven M. Paul, president of Lilly Research Laboratories, discussed how the dual challenges of rising costs and realizing the potential of biomedical research have been reflected in the recent experience of biopharmaceutical companies. Stakeholders are demanding more information on the effectiveness of therapies, as well as more predictable and demonstrable health outcomes. Despite these heightened expectations, there is an efficacy and safety gap for today's drugs. Paul noted that only about 50 percent of patients respond to any given therapy, and many of these do not respond in the same way. **This increased focus on outcomes has put a burden on biopharmaceutical research development in terms of both longer development timelines and overall costs.**	
NESTA	Total Innovation. Why harnessing the hidden innovation in high-technology sectors is crucial to retaining the UK's innovation edge	2008		Pharma regulatory approval		In pharmaceuticals, the speed and efficiency with which drugs pass through regulatory stages has a major impact on the returns to pharmaceutical firms' investments. Every day that a product is delayed reaching the market can cost $1-3 million in lost revenue. But, understandably, regulators tend to focus on establishing the safety of new products rather than encouraging investment in new product development.	
				Different firms responsible at different stages of development	Collaboration so that stages can take place in parallel	In aerospace, major manufacturers such as Airbus and Boeing are increasingly designing new aircraft in collaboration with suppliers, rather than designing them in-house and passing blueprints for parts or sections to suppliers. For example, 6,000 engineers around the world are effectively jointly designing and engineering the new Boeing 787 aircraft. This has cut the typical four or five year time from concept to production by a year, and reduced development costs by 20 per cent.	
				Increased complexity of clinical trials and regulatory submissions	Establishment of CROs:<br>New clinical research management organisations (CROs) now run many of the clinical trials required for regulatory approval. They account for more than 40 per cent of annual research spending by firms, compared to just 4 per cent in the early-1990s.16 Many CROs are based in Eastern Europe, India and China. In recent years, their portfolio of services has widened to include more advanced technologies such as genomics and high-throughput (automated) screening, using proprietary techniques for which they often own the intellectual property rights. CROs can also offer post-commercialisation services such as sales and marketing.	The role of CROs has grown rapidly as a result of the increasing pressure on pharmaceutical firms to bring drugs to market more quickly and the increased complexity of clinical trials and regulatory submissions. The ability of CROs to cut clinical trial times by as much as 30 per cent has become critical to large firms, especially given their concerns over R&D productivity and generic competition. CROs are more able than most firms to conduct studies on a multinational and multi-centre basis.	
Darzi/DH	High quality care for all: NHS Next Stage Review final report	2008		Slow uptake		In this country, we have a proud record of invention, but we lag behind in systematic uptake even of our own inventions	
				Delayed NICE guidance on new drugs		It has sometimes taken too long for NICE appraisal guidance to be made available on newly licensed drugs. Guidance has often been published two years or more after a new drug's launch, though NICE has now put in place a faster appraisal process for key new drugs which enables it to issue authoritative guidance on them within a few months of their UK launch.	
UKCRC	Progress Report 2006-2008	2008		Disincentives delay research in the NHS	Measures to address include:<br>- development of model agreements between research funders and NHS organisations, to reduce bureaucracy and facilitate faster initiation of research projects<br>- roll-out of Research Passport for honorary contracts across the NHS<br>- streamlining the research ethics system		
Pharmaceutical Industry Competitiveness Task Force	Ministerial Industry Strategy Group Pharmaceutical Industry: Competitiveness and Performance Indicators 2009	2009		Trial recruitment		There is room for improvement in the speed with which trials are conducted and get started. A little under half of the UK arms of clinical trials are managing to recruit the intended number of patients within the planned timescale (indicator 7), and the median time lag between first submission of the protocol for a proposed clinical trial and the first patient being seen in that trial is 150 days in the UK	

Author	Title	Year	Time lag	Reasons for time lags	Measures to address	Quotes from document	Notes
Wellcome Trust	Response to REF consultation on impact	2009	17			The Trust's view is that the challenges associated with time lags and attribution will be very difficult to address... They are particularly challenging issues for biomedical research, where the time frame to commercial uptake of medical products and technologies is typically very long. The independent report Medical Research: What's it worth?, part-funded by the Wellcome Trust, found an average time lag of 17 years between research and impact.	
IOM	Transforming Clinical Research in the United States: Challenges and Opportunities: Workshop Summary	2010				Because clinical research relies on substantial human effort that incurs large labor costs, the timeline for a clinical trial affects overall cost. DiMasi and colleagues estimated that in 2000, the average cost to develop a new drug was $802 million, and time costs associated with the length of research and development accounted for half of this cost (DiMasi et al., 2003).<br><br>For the pharmaceutical industry, protracted timelines increase cost and reduce revenue as medications typically have a finite life before losing patent protection and creating an opportunity for generic competitors. Moreover, when a trial addresses a question important for medical practice, increasing the time it takes to obtain an answer can reduce the impact of the results. Musa Mayer, breast cancer survivor, advocate, and author (AdvancedBC.org), commented that if clinical trials are subject to significant delays, the standard of care can move on in the absence of phase III data. Thus, obstacles and delays in clinical trials move health care further away from evidence-based practice. Moreover, if the time lag is significant, the results of a lengthy, expensive trial may already have been rendered irrelevant by changes in clinical practice when they finally become available.	
				One-off nature of trial organisation increases timeline for clinical trials	Renzo Canetta, Vice President of Oncology Global Clinical Research, Bristol-Myers Squibb, provided an example of the internal administrative burdens faced by industry. Historically, Bristol-Myers Squibb has required 8 months, or 34 internal review cycles, to produce and activate a new study protocol. Recent efforts to improve the review cycle have been aimed at reducing this internal process to 150 days (5 months). Some individual institutions have exhibited greater flexibility and have been able to further streamline the protocol approval process. The University of Arkansas has a 70-day timeline for activating a new trial, while M.D. Anderson Cancer Center has a project under way (Project Zero Delay) to turn protocols around in 46 days, according to Canetta.	The one-off nature of trial organization, mentioned by a number of workshop participants as a major barrier to the efficient conduct of trials, is one factor leading to prolonged trial startup times. Years can elapse from the time researchers begin talking about a study idea to the point at which they assemble the appropriate investigators, develop collaborations, establish study sites, and initiate the trial.	Also has ideas on standardising case report forms and education patients about clinical trials to help recruitment
						By pooling the resources of multiple entities, CRNs can realize efficiencies in implementing and conducting clinical trials. They create a supportive infrastructure for investigators and can facilitate the rapid conduct of trials to answer important research questions. For instance, CRNs organized around a particular disease often have access to patients with that disease who can serve as study participants. The in-house scientific leadership of CRNs can also streamline the protocol development process and create uniformity in clinical trials across the network or disease area. **When clinical trials from a particular network generate consistent results, this can also accelerate the drug development pipeline for the disease studied.**	
					Global clinical trials to accelerate recruitment, local product approval (p52)		
BIS/DH	Life Sciences in the UK – Economic analysis and evidence for 'Life Sciences 2010: Delivering the Blueprint'	2010	10-15 yrs for drug development; 3-18 months for medical devices; 3-5 yrs for in vitro diagnostics<br><br>Around 2 yrs on average from application to			The drug discovery process usually takes around 10–15 years, although in some cases it can be significantly longer.	
				Lack of awareness of new technology		In many cases **it is possible that medical practitioners and commissioners of healthcare will not become immediately aware of new technologies causing a lag in their utilisation** and consequent welfare losses.<br>Government intervention can mitigate this problem of information transmission, minimising the lag and reducing the welfare losses due to failure to use available cost-effective medicines and technologies by imposing minimum standards and regulatory requirements.	

Author	Title	Year	Time lag	Reasons for time lags	Measures to address	Quotes from document	Notes
			launch in UK drug market.	Companies not understanding patients' needs and how their technology can address	The NHS can reduce these losses by providing clearer signals to firms about what is needed and working with them to determine how new technologies can meet these needs. This might be achieved through greater engagement and better communication of the clinical and, therefore, commercial value of different treatments	Eventually the market will reach a new point where all the beneficial products and services are provided but any delay in reaching this outcome will result in a welfare loss. Delays can be caused by a lack of understanding by firms of the needs of patients and how the technology at their disposal can be used to help them.	
					CRNs	In 2008/9, the NIHR invested £135 million in Biomedical Research Centres and Units across England. **On top of this, it spent £158 million in supporting Clinical Research Networks that accelerate the development of new ways to prevent, diagnose and treat important conditions**, and £116 million on commissioning research and studies designed to help identify best clinical practice.	
					Partnerships between academia and industry	The DSTT is a partnership between the pharmaceutical companies and thirteen research teams based at the University of Dundee, eight of which are within the MRC Protein Phosphorylation Unit. This brings the funding raised by DSTT to a total of £23 million since 1998, and assists in translating recent research findings and ideas into potential new treatments for cancer, hypertension and Parkinson’s disease. The aim of the DSTT is to accelerate the development of improved drugs to treat global diseases such as cancer, diabetes and rheumatoid arthritis and which exert their effects through two types of enzymes; ‘kinases’ and ‘phosphatases’. The market for drugs that act on kinases is estimated to be worth £7 billion a year and projected to reach £33 billion by 2010’	
					NIHR (i4i) Funding Programme brings together the work of several smaller programmes in a new responsive investment stream to help accelerate the development of new healthcare technologies and device	Funding for the programme has been gradually increased from £4 million in 2006/07 to £13 million in 2009/10. Activities included under the umbrella of i4i include the following: i4i Future Product Development funding streams, which includes a funding stream that requires co-funding of proposals by industry; Pilot Healthcare Technology Co-operatives, involving NHS, academia and industry working closely together to develop clinically needed, cost-effective health technology products in neglected areas with high disease burden; and The Small Business Research Initiative (SBRI): part of the revised Cross-Government SBRI, the i4i programme leads on the development of a pilot programme in the area of Health Care Associated Infections. A number of phase 1 SBRI projects have been selected for funding and further areas are under consideration.	
				Trial start-up and completion times		According to Kinapse (2008) an international comparison of time taken to begin trials is difficult, and there is no evidence that the UK performs any worse than other countries. However, the industry has reported in recent work on clinical trials that there is a need to improve performance on the time taken to set-up clinical trials in the UK. There is historical evidence to suggest that the UK has not performed well in terms of the reliability of its execution of clinical trials, with only 29% completed within planned timelines, compared to 77% internationally. However, enhanced performance has been reported via National Institute for Health Research Clinical Research Network (NIHR CRN). In the last quarter of 2009, 80% of contract commercial trials were delivered on time and target. Some 4% of sites were non-recruiting.	
						According to NESTA (2008) 'the role of contract research organisations has grown rapidly as a result of increasing pressure on pharmaceutical firms to bring drugs to market more quickly and the increased complexity of clinical trials and regulatory submissions. **The ability of CROs to cut clinical trial times by as much as 30%** has become critical to large firms, especially given their concerns over R&D productivity and generic competition. CROs also tend to be more able to conduct studies on a multinational and multi-centre basis.	
Office for Life Sciences	Life Sciences 2010: Delivering the Blueprint	2010		NICE appraisal time	National Institute for Health and Clinical Excellence’s (NICE) engagement in the Research Capability Programme will enable NICE to use data that is routinely collected by the NHS to speed up its appraisal process		

Additional file 2: Review of policy documents to identify the time lags highlighted. *Health Res Policy Syst 2015;* **13:**1

Author	Title	Year	Time lag	Reasons for time lags	Measures to address	Quotes from document	Notes
				Difficult for SMEs to access NHS procurement market	Small Business Research Initiative (SBRI)	For the first time, in September 2009, the National Innovation Centre (NIC) proactively sought solutions from SMEs to needs that had been directly identified by clinicians. The NIC ran online competitions to address three Ambulance Service and three paediatric clinical needs identified in Spring 2009. **NIC offers a range of pre-commercial approaches for its competitions, one of which is SBRI, to speed up the procurement of innovation in the NHS.** Twenty companies entered the competitions; these were predominantly SMEs but also included some individual innovators. Nine contracts were awarded to both SMEs and individual innovators. Each development is closely project-managed by NIC, with updates provided and further collaborations facilitated via NIC's website. **The aim is that this assertive innovation development process should take a product from clinical need to market readiness within 18 months.** NIC is planning to launch at least wo more rounds of competitions in 2010/11. SMEs that already have technology innovations can carry out their own assessment of their products on NIC's technology-ready Scorecard. The NIC Scorecard is reviewed by NHS Supply Chain for placement of products on its NHS catalogue, offering the possibility that the technology will be sold across the NHS.	
				Excessively bureaucratic processes for setting up research and trials in some NHS Trusts and R&D department	Set up of NIHR Research Support Services to support CRNs		
				Excessively bureaucratic processes for setting up research and trials in some NHS Trusts and R&D department	The North West Exemplar Programme was initiated by the NIHR/NHS Biopharmaceutical Industry R&D Leadership Forum as a response to emerging evidence that the UK is losing relative market share of the global biopharmaceutical R&D market,	The Programme is designed to provide hard evidence and case studies of improved performance, particularly around recruitment reliability and trial cycle time. The knowledge and experience gained from this Programme will spread good practice more widely across the NHS, NIHR and industry .<br><br>The time between the first submission for R&D approval and issue of the NHS permission letter has been a median time of 51 days. This has been positively viewed by industry sponsors Novartis, GlaxoSmithKline, Abbott, Boehringer Ingelheim, Schering-Plough and Janssen- Cilag, because it has exceeded their original target of 70 days. For the first time in five years, the UK has achieved recruitment of the first patient in a global trial. This success is coupled with exceptional timelines, with initial submission for UK regulatory approval for first-patient-first-visit completed within nine weeks.	
			>10 yrs			Government will invest £100 million in a £200 million, at first closing, technology fund of funds to be managed by the EIF which will cover Life Sciences, as well as digital/ ICT and advanced manufacturing. Further private investment will be secured before the Fund closes for investors in 2011. One of the largest pools of venture capital for investment in technology funds in Europe has now been raised, and the ambition remains to create a £1 billion, 15-year Fund. **The extension to the life of the Fund, from 10 to 15 years, is a direct result of feedback from the Fund of Fund Managers and the Life Sciences industry recognising the long timescales involved in new product development.**	
DH	Innovation, Health and Wealth: Accelerating adoption and diffusion in the NHS ('The Nicholson Review')	2011					Sets out various structures, processes, etc for accelerating adoption in the NHS.
ESF	EMRC White Paper: A Stronger Biomedical Research for a Better European Future	2011	17			The study crucially also showed that the time lag between research funding and health return is approximately 17 years. A similar study performed in the US found that every US$1 spent by the National Institutes of Health (NIH) typically generates US$2.21 in additional economic output within 12 months	

Additional file 2: Review of policy documents to identify the time lags highlighted. *Health Res Policy Syst 2015;* **13:**1

Author	Title	Year	Time lag	Reasons for time lags	Measures to address	Quotes from document	Notes
Russell Group	The social impact of research conducted in Russell Group universities	2012	17			The pathway from research to impact is complex and more often realised through indirect routes than a linear path. There may be many players involved along the way, **the time lag before a benefit is realised can be many years, and there is often more than one stage and level of impact**.<br><br>The importance of taking a long-term view is critical to supporting the nation's research infrastructure. **Significant time lags between research and its impact are the norm. It is the nature of research that it is iterative, that it does not stand still, and will continue to push boundaries during its lifespan. A study of research into cardiovascular disease found that it takes on average 17 years for basic research to be translated into treatment benefits.**	
Innovative Medicines Initiative	IMI Factsheet	Undated	10-13 for drug development			The drug development process is very long, complex and costly:<br>Only one drug candidate in 10,000 will reach the market/patient.<br>It takes up to 10 -13 years to develop a drug and bring it to the market.<br>It requires an investment of around €1 billion on average for each new drug.	(accessed 2013)
