# Supplementary material for: How long does biomedical research take? Studying the time taken between biomedical and health research and its translation into products, policy, and practice
Source: Health Res Policy Syst. 2015 Jan 1;13:1. doi: 10.1186/1478-4505-13-1 (PMC4297458; doi:10.1186/1478-4505-13-1)
Supplement: Supplementary file 3 — Additional file 3: Seven case studies of time lags between conducting medical research and its translation. This file contains all seven case studies in full; each consisting of a brief narrative account, a timeline, a version of the matrix, and a reflection on methodological issues that arose. (PDF 3 MB) [file 12961_2014_368_MOESM3_ESM.pdf]

Additional File Three in:

Hanney SR, Castle-Clarke S, Grant J, Guthrie S, Henshall C, Mestre-Ferrandiz J, Pistollato M, Pollitt A, Sussex J, Wooding S: **How long does biomedical research take? Studying the time taken between biomedical and health research and its translation into products, policy, and practice.** *Health Res Policy Syst* 2015.

Seven Case Studies of time lags between conducting medical research and its translation.

Page 3: Case Study 1: Amlodipine

Page 21: Case Study 2: Olanzapine

Page 37: Case Study 3: Screening programme for Abdominal Aortic Aneurysms (AAA)

Page 51: Case Study 4: Smoking reduction

Page 79: Case Study 5: CBT and depression

Page 95: Case Study 6: CBT and schizophrenia

Page 119: Case Study 7: Early intervention in schizophrenia

Case Studies Time lags between conducting medical research and its translation. Case Study 1: Amlodipine

NARRATIVE ACCOUNT OF BACKGROUND/ DEFINITIONS AND KEY ASPECTS OF THE INTERVENTION'S DEVELOPMENT

Background/definition

Amlodipine is an antihypertensive drug, originally sold by Pfizer under the brand name Norvasc® in the USA and Istin® in the UK but today available generically. It is a long acting calcium channel blocker, CCB, (a "3rd generation" CCB) belonging to a class of compounds called dihyrdopyridines (DHP). It is approved for treating hypertension and chronic stable and vasospastic angina. It acts by relaxing blood vessels and improving blood flow.

Pre-commercial research:

The early research on the DHP class of compounds (in the late 1950s) proceeded on a parallel track with the discovery of the calcium channels (the target of DHPs). The scientists working on DHP were not aware of the mechanism of action of the compound class (i.e. they did not know how the compound worked), but they did know that it worked so they conducted physiological testing to select the best compounds to be used in humans. This is also underlined in the narrative of the discoveries of the calcium channel target and the DHP, which do not cite each other.

According to Tsien and Barrett in "A brief history of calcium channel discovery" (2000):

> "Where did the notion of a calcium channel first start? Clearly, it was in the Biophysics Department at University College London, founded by A.V. Hill and later led by Bernard Katz. Most investigators would credit Katz, Paul Fatt and Bernard Ginsborg for pioneering the calcium channel field with their work in large muscle cells of crab and crayfish. These three were charter members of a small group of electrophysiologists who clarified many aspects of nerve propagation and synaptic transmission. But despite the pedigree of these investigators, calcium channels were not a major source of attention in the early 1950s, despite their now clearly critical role in even the simplest neuromuscular circuit. Indeed, the discovery of Ca^{2+} spikes aroused no great excitement in the 1950s, nor even many years later, when the accumulated triumphs of electrophysiology were reviewed."

Godfraind in "Milestones in drug therapy: calcium channel blockers" (2004) makes no mention of any of the above names. But he reproduces a "chronology of calcium research" from Ebashi (1993) (containing a high proportion of Japanese-sounding names) which includes the event "The concept of Ca^{2+} antagonist emerges" dated at 1969 and credited to Fleckenstein and Godfraind (!).

In the late 1960s, Reuter reconciled the two strands of research and demonstrated the importance of calcium channels as target of the calcium blockers and reported their presence in heart cells (Reuter, 1967, 1973).

Commercial research & development

Following the introduction of 1st generation DHPs, extensive work on second generation drugs ensued. This led to the introduction of a number of agents including amlodipine, benidipine, cilnidipine, felodipine, isradipine, lacidipine and lercanidipine etc. (Epstein, 2002; McDonough, 2004). Among the various calcium channel blockers (CCBs), amlodipine is of particular interest because it is generally well tolerated and often used in combination with diuretics, angiotensin-

converting enzyme inhibitors, angiotensin II receptor antagonists and statins (Schulman et al., 2005; Jukema and van der-Hoorn, 2004; Basile, 2004).

Non-clinical research

The amlodipine programme was in part stimulated by Pfizer Inc. licensing nifedipine for the treatment of angina in the US from Bayer, the inventors and produce licence holders in Europe. The product was called Adalat in Europe and Procardia in the US. David McGibney thinks this was in the late 1970s (DM, personal communication to OHE).[1] Pfizer efforts received a boost when they licensed a controlled release technology from Alza. This allowed three times daily Procardia to be further developed into a once a day drug for the treatment of angina and hypertension (Procardia XL).

The commercial success of Procardia XL provided a big stimulus to the discovery of a dihydopyridene calcium antagonist with intrinsic long duration of action and amlodipine was the result. Amlodipine was ‘discovered’ as a result of experiments in dogs, which metabolised a dyhdropyridine (DHP) product into a long-acting substance - Amlodipine. Pfizer’s preclinical programme for Amlodipine commenced in 1979.

A Google search identified Dr Simon Campbell CBE FRS FMedSci, who retired as Pfizer’s head of research in 1998, as a key player: “Innovative research by Dr Campbell and his team at Pfizer Sandwich in Kent led to the discovery of amlodipine…” (taken from the Birmingham University Outstanding Alumni website). David McGibney confirmed the key role of Dr Simon Campbell (DM, personal communication to the OHE).

On or about August 11, 1982, the project of formulating a commercial drug product was assigned to Dr James Wells, a manager in Pfizer's Pharmaceutical Research and Development Department, who was assisted by Mr Edward Davison, a member of the same group.

Clinical research

David McGibney gave the first dose of Amlodipine maleate to humans on or about January 6th 1983 in Prof Brian Prichard’s laboratory in University College hospital. Dr McGibney was both the representative of the sponsor (Pfizer) and also the attending physician and the single ascending dose study was completed 3 months later. A multiple dose volunteer study followed and short term studies in angina and hypertension started in Leeds, Belgium and Germany in the second half of 1983. Dr McGibney’s involvement with amlodipine maleate continued as head of the European clinical cardiovascular group in Sandwich until it was filed and approved

By April 24, 1984, Dr Wells identified a formulation for amlodipine maleate that produced “excellent capsules”. In attempting to produce a direct compression tablet product of an amlodipine maleate formulation, however, Dr Wells encountered two problems: (1) chemical instability of the amlodipine maleate, and (2) stickiness of the tablet blend of amlodipine maleate. Chemical stability refers to the resistance of a drug compound to chemical breakdown, while stickiness refers to the adherence of the drug substance, in formulation, to manufacturing equipment, such as the punch

[1] David McGibney, a physician, joined Pfizer (at Sandwich) in August 1982. He joined the project team for amlodipine (Pfizer project name: UK48340) then. Project Lead Biologist was Roger Burges (now deceased); Lead Chemist was Simon Campbell (retired).

faces of a tablet-making press. To solve the problems of the tablet form of amlodipine maleate, Dr Wells suggested that other amlodipine salts be made and tested.

By April 30, 1985, both amlodipine maleate and amlodipine besylate were undergoing human testing in clinical trials. Pfizer scientists predicted that the capsule form of amlodipine maleate would have a shelf life of three years, but that "poor stability of amlodipine maleate tablet formulations" precluded commercialization. On the other hand, the scientists noted that amlodipine besylate tablet formulations exhibited "clear superiority" in their processing characteristics, particularly non-stickiness, and in stability.[2] Capsule formulations of amlodipine besylate had not yet been produced, but work on this project was "expected to be straightforward."

Patent history:

- March 11, 1982, Pfizer filed a patent application in the UK for amlodipine.
- March 8, 1983, European Patent applied for, awarded October 15, 1986. Title: "Dihydropyridine anti-ischaemic and antihypertensive agents, processes for their production and pharmaceutical compositions containing them". Inventors: Campbell SF, Cross PE, Stubbs JK.
- February 25, 1986, Pfizer Inc. awarded US Patent No. 4,572,909 (the '909 patent), which is the US counterpart of the 1982 UK patent, and which discloses ten pharmaceutically-acceptable acid addition salts of amlodipine and describes the preferred salt as being maleate. Amlodipine is a member of a class of compounds known as dihyrdopyridines (DHPs). Like many active drugs, amlodipine is made into a pharmaceutically-acceptable acid addition salt to improve its bioavailability.
- April 4, 1986, Pfizer filed a patent application for amlodipine besylate in the UK, which was eventually issued as UK Patent No. 160833.
- March 31, 1987, European Patent application for amlodipine besylate, eventually granted January 24, 1990. Title: "Salts of amlodipine". Inventors: Davison E, Wells JI.
- March 25, 1987, Pfizer Inc. filed a US patent application claiming amlodipine besylate. It was initially rejected but ultimately awarded in …
- November 7, 1989, Pfizer Inc. awarded US Patent No. 4,879,303 (the '303 patent), which relates specifically to amlodipine besylate (sold under the trademark Norvasc®). The active ingredient in Norvasc® is 2-[(2-aminoethoxy)methyl]-4-(2-chlorophenyl)-3-ethoxycarbonyl-5-methoxycarbonyl-6-methyl-1,4-dihydropyridine, commonly referred to as amlodipine. Amlodipine besylate is an acid addition salt of amlodipine formed by reacting amlodipine with benzene sulphonic acid.

Launch and market history:

Amlodipine was launched by Pfizer in 1990 in the UK and November 1992 in the US, as NORVASC®.

The rate of uptake is shown in the graph below, measured in prescription items in primary care in England. Post-launch there was steady growth, with an acceleration after 2004, when Norvasc® lost market exclusivity (went generic) in the UK.

[2] Dr McGibney confirmed that the switch from maleate to amlodipine besylate occurred later for ease-of-manufacture reasons (the maleate powder was sticky).

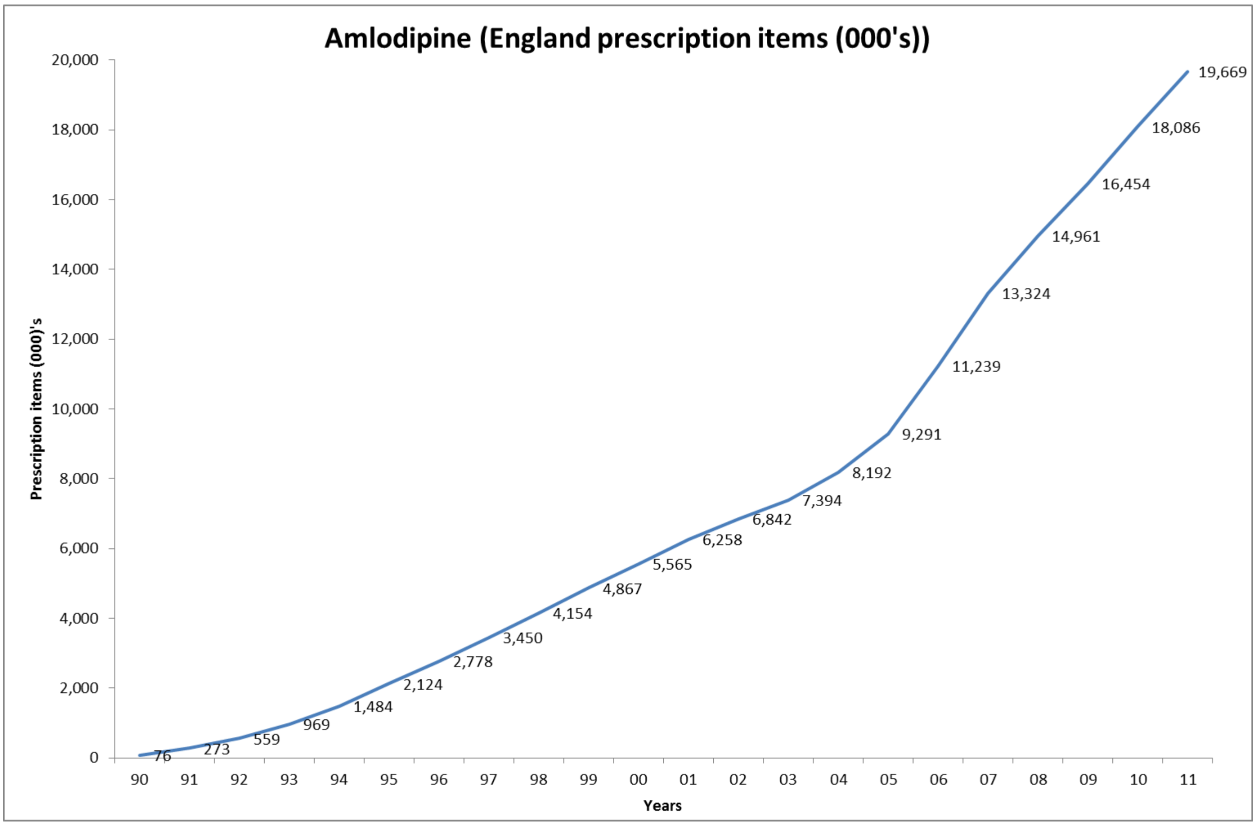

Source: author elaboration from Prescription Cost Analysis – England data.

National policy announcement / guidelines / advice

Key guideline produced for NICE: National Collaborating Centre for Chronic Conditions. Hypertension: management in primary care: pharmacological update. London: Royal College of Physicians, 2006.

Previously, NICE considered amlodipine (and/or calcium channel blockers) in the following guidelines:

- 2001: Prophylaxis for patients who have experienced a myocardial infarction: drug treatment, cardiac rehabilitation and dietary manipulation - guideline.

  "Calcium channel blockers, nitrates, and potassium channel activators have no effect on premature mortality making their role the management of symptoms and risk factors (principally hypertension) (A). They should therefore only be used in those patients who are intolerant of beta-blockers and ACE inhibitors (D). Given their effect on non-fatal myocardial infarction, verapamil or diltiazem should then be considered initially (B). Subsequent necessary treatment with other calcium channel blockers [including amlodipine?]*, nitrates or potassium channel activators is then appropriate (D)."* (NICE, 2001)

- 2003: Chronic heart failure - Management of chronic heart failure in adults in primary and secondary care.

  "Amlodipine should be considered for the treatment of co-morbid hypertension and/or angina in patients with heart failure, but verapamil, diltiazem or short-acting dihydropyridine agents should be avoided." (NICE, 2003)

- 2004: Essential hypertension: managing adult patients in primary care.

  "Drug therapy should normally begin with a low dose thiazide-type diuretic. If necessary, second line add a beta-blocker unless a patient is at raised risk of new-onset diabetes, in which case add an ACE-inhibitor. Third line, add a dihydropyridine calcium-channel blocker." (NICE, 2004)

Analysis of 2006 guideline indicates a range of points that might be relevant for the cases study, and in some cases more generally for our time lags project:

1. 12 out of 20 studies included Calcium Channel Blockers (CCB) in one of the arms, but only 3 out of the 12 used Amlodipine. In this hypertension guideline, the main recommendations about use of CCBs did not differentiate between different CCBs because the evidence was pooled, nevertheless the detailed analysis could possibly indicate at least to some degree the contributions made by the different trials and the rapid update of the previous guideline from just 2 years before was driven by the ASCOT trial of Amlodipine (see below), and the health economics analysis makes a distinction between some of the CCBs based on cost. [The question about which specific drug used in trials cited on guidelines is a specific issue in this case study and perhaps a general one for the time lags project].
2. The main recommendations of the 2006 guideline included: the first one: 'In hypertensive patients 55 or over, or black patients of any age, the first choice for initial therapy should be calcium-channel blocker or a thiazide-type diuretic.' (p.17)
3. This was a considerable change from previous NICE Clinical Guideline (018) published in August 2004 that said: 'Drug therapy should normally begin with a low dose thiazide-type diuretic……….Third line, add a dihydropyridine calcium-channel blocker [which includes Amlodipine].' (p.14)
4. The reason for such a rapid update of part of the guideline was because of the evidence from new trials (see opening sentence of 2006 guideline) especially the potentially significant new evidence that was anticipated to be coming from the ASCOT trial of Amlodipine as described in the following which might provide a good example of action NICE took to reduce time lags by acting on anticipated evidence: http://www.onmedica.com/newsarticle.aspx?id=627a0c87-e72a-451a-8660-de23e6dcd541
5. Of the 3 trials including Amlodipine in 2006 guideline only one (ALLHAT) had been included in the 2004 guideline; and the 2 additions that included Amlodipine (VALUE and ASCOT) constituted half of the total of 4 additional trials included in the 2006 Update. (One other was a different CCB, and 4 studies from the 2004 guideline were dropped – p.5).
6. ALLHAT trial was a complex North American trial involving several different comparisons each reported in several papers but all mainly funded by NIH but drug companies including Pfizer provided the drugs. One 2002 paper described comparisons of ACE inhibitor, CCBs and diuretic in high-risk patients and concludes: 'Thiazide-type diuretics are superior in

preventing 1 or more major forms of CVD and are less expensive. They should be preferred for first-step antihypertensive therapy.' (ALLHAT, 2002)

7. VALUE trial was international including UK but led from USA designed to 'test the hypothesis that for the same blood-pressure control valsartan [a Novartis ARB] would reduce cardiac morbidity and mortality more than amlodipine in hypertensive patients at high cardiovascular risk.' (p.2022). Published in 2004 and funded by Novartis it found the main outcome of cardiac disease did not differ but reported: 'Blood pressure was reduced by both treatments, but the effects of the amlodipine-based regimen were more pronounced,' (Julius S et al., 2004)
8. ASCOT trial was an Anglo-Scandinavian trial led from Sweden comparing amlodipine with atenolol, a BB. Funded mainly by Pfizer, New York. Published in 2005. (Dahlof B et al., 2005, p.895). As stated in the paper, patient recruitment started in February 1998 and finished May 2000. Follow up was to be for 5 years. 'In Oct 2004, the DSMB recommended the trial be stopped on the grounds that compared with those allocated the amlodipine-based regimen those allocated the atenolol-based regimen had significantly higher mortality … between December 2004, and June, 2005, the trial doctors recalled all patients for a final end-of-study visit.' The paper was published Sept 2005.
9. In the economic analysis for the 2006 guideline the cost data for the CCB used in the model was Amlodipine (p.70) because in all cases the prices of the most commonly used drug was included in the model. The health economics slightly favoured CCBs, but 'the more expensive brands are not likely to be cost effective for use in the NHS.' (p78)
10. The average time lag between publication of these 3 papers (2002, 2004, 2005) and guideline (2006) is much shorter than 'knowledge cycle time' average for guideline as a whole in *Medical Research: What's it Worth* (Buxton et al., 2008) of 9.5 years. But in that report it was also claimed there was an average 3 year gap between funding and publication, but in these 3 papers the gap between recruitment of first patient and publication is much longer (8 years, 7 years, 7 years respectively, notwithstanding the early end of the ASCOT trial and publication within four months of the end of data collection).

Then there are issues about the further update of the guideline in 2011 and more positive recommendations for CCBs because of the considerable reductions in price (which made it more cost effective for the NHS than doing nothing).

TIMELINE

Period/date	Study(ies)/Event	Tracks	Calibration	Comments
Early 1950s	Voltage-Gated Calcium Channels (the target of the blockers) were discovered accidentally by Paul Fatt and Bernard Katz in neuromuscular transmissions in crab legs	**Discovery**	**Publication:** Fatt and Katz (1953)	These studies are cited in the book: Godfraind, T. (2004). *Calcium channel blockers*. Birkhäuser.<br>Apparently, all these studies have been important contributions to understand the mechanism of action of the calcium channel blockers.
Late 1950s – early 1960s	First investigations into the pharmacology of calcium antagonists (unaware of their targets)	**Discovery**	**Publications:** Evans et al. (1958); Durbin and Jenkinson (1961)	
1960s	Fleckenstein and Godfraind demonstrated on arterial tissue the concept (and the mechanism of action) of calcium antagonists	**Discovery**	**Publications:** Fleckenstein et al. (1969); Godfraind and Kaba (1969)	
Late 1960s – early 1970s	The importance of Voltage-Gated Calcium Channels as target of the calcium blockers and their presence in heart cells is reported	**Discovery**	**Publications:** Reuter (1967, 1973)	
Late 1960s - Late 1980s	R&D of the first generation of Dihydropyridine (DHP) calcium channel blocker nifedipine	**Discovery**	**Patent approval:** US Patent Number 3,485,847 issued on 23 December 1969<br>[**Regulatory approval of the first generation calcium channel blocker:** Procardia XL (Pfizer), NDA application number 19-684 on 6 September 1989]	During the project workshop in January 2013 we agreed to consider the approval date of the first generation calcium channel blocker (amlodipine is a third generation calcium channel blocker). Here I am considering the first generation calcium channel blocker patent approval in the Discovery track.

1978 1980 - 19791981	Pfizer's Discovery Chemistry group, located in Sandwich (England) invented amlodipine maleate (compound No. UK-48,340-11) and discovered its anti-hypertensive and anti-ischemic pharmacological properties	**Discovery**		This information is provided in the US Court Case FFCL223, par. 53, which does not specify the date of discovery.<br>David McGibney, involved in the project since August 1982, stated he believes that the discovery took place in 1979-1980, possibly 1978 1981 (David McGibney, personal communication).
11 March 1982	Pfizer filed a patent application in the UK for amlodipine maleate	**Discovery**	**Patent filing:** GB8207180 (11th March 1982)	I did not find the UK patent, however the date of the UK patent filing is confirmed by the US patent, which I have.<br>I assumed that patents are calibration points for the discovery track (they can attest when a new compound was created). In general, a patent is filed before starting clinical tests (Human research track). However, there are cases where new patents follow the first one and this implies that the Discovery track could overlap with other tracks because I arbitrarily defined patents as a calibration point for the Discovery track.
14 July 1982	The Discovery Chemistry group recommended that an effort be made to develop amlodipine into a commercial product			This information is provided in the US Court Case FFCL223, par. 57.
11 August 1982	The project of formulating a commercial dosage form of amlodipine maleate was assigned to Dr James Wells and Mr Edward Davison (Pfizer R&D group)			This information is provided in the US Court Case FFCL223, par. 59-64.
Prior to 1983	Amlodipine maleate is tested on guinea-pigs	**Discovery**	**Publications:** Beattie et al. (1984,1985)<br>**Cited on:** Beattie et al. (1986)	David McGibney also mentioned tests in dogs (David McGibney, personal communication)

6 January 1983	First dose to humans in Prof Brian Prichard's laboratory in University College hospital	**Human research: first in human/safety (Phase I)**	**Possible citation on:** Jackson et al. (1985)<br>**Personal communication:** David McGibney	I could not retrieve the paper, so I cannot say for sure that the Jackson et al. paper is a calibration point for these studies
8 March 1983	Amlodipine maleate European Patent applied for	**Discovery**	**Patent filing:** European Patent Application No. 83301227.1 (8th March 1983)	
End of 1983	Pfizer's Analytical Chemistry Department diagnosed an instability problem associated to amlodipine maleate			This information is provided in the US Court Case FFCL223, par. 70.
1984	Chasseaud and Taylor conducted Phase I studies in healthy volunteers to study the pharmacokinetics of amlodipine maleate (single IV/oral and repeated oral)	**Human research: first in human/safety (Phase I)**	**Cited on:** Faulkner et al. (1986)	The paper of Faulkner et al. is not the publication of the studies but a publication citing the studies. In the original set of calibration points, we had a "cited on" calibration point. However, the last version of the minimum set of calibration points did not included this option.
3 February 1984	Amlodipine maleate US patent filed	**Discovery**	**Patent filing:** US Patent application No. 576,982 (3rd February 1984)	
Second half of 1984	Dr Robin Platt concluded that amlodipine besylate salt formulation is significantly more stable than the maleate salt			This information is provided in the US Court Case FFCL223, par. 78.
11 October 1984	Dr Wells recommended the R&D group that the amlodipine besylate salt be substituted for the maleate salt in the commercial amlodipine tablet product			This information is provided in the US Court Case FFCL223, par. 120.

End of 1984	Pfizer was conducting Phase II clinical trials of amlodipine maleate (using capsules and intravenous injections)	**Human research: dosage/design (Phase II)**	**Possible citation on:** Jackson et al. (1985)<br>**Possible citation on:** Webster et al. (1987)	This information is provided in the US Court Case FFCL223, par. 121.<br>I could not retrieve the Jackson et al. paper, so I am not sure if these Phase II clinical trials are cited in that paper.
1984-1986	Phase II double-blind placebo-controlled studies to investigate the efficacy of once daily amlodipine maleate in patients with mild to moderate hypertension.	**Human research: dosage/design (Phase II)**	**Publication:** Webster el al. (1987)	Web search using appropriate filters in: PubMed and Cochrane Library
25 February 1986	Amlodipine maleate US patent awarded	**Discovery**	**Patent approval:** US Patent Number 4,572,909 (25th February 1986)	
4 April 1986	Amlodipine besylate UK patent filed	**Discovery**	**Patent filing:** GB8608335 (issue No. 160833) (4th April 1986)	
5 May 1986	Pfizer filed an amended Investigatory New Drug application with the FDA which reflected a switch in salts from amlodipine maleate to amlodipine besylate	**Regulatory approval / first non-research use in patients**		This information is provided in the US Court Case FFCL223, par. 125.
15 October 1986	Amlodipine maleate European Patent awarded	**Discovery**	**Patent approval:** European Patent Publication No. 0 089 167 (15th October 1986)	
1986 – 1989	Phase III clinical studies	**Human research: efficacy (Phase III)**		I did not find any study for this event.
31 March 1987	Amlodipine besylate European patent filed	**Discovery**	**Patent filing:** European Patent application No. 87302767.6 (31st March 1987)	

13 October 1988	Amlodipine besylate US patent filed	**Discovery**	**Patent filing:** US application No. 256,938 (13th October 1988)	
8 March 1989	Belgium: first regulatory approval of amlodipine besylate as Norvasc	**Regulatory approval**	**Regulatory approval cited on:** EMA (2011)	
7 November 1989	Amlodipine besylate US patent awarded	**Discovery**	**Patent approval:** US Patent Number 4,879,303 (7th November 1989)	
24 January 1990	Amlodipine besylate European Patent awarded	**Discovery**	**Patent approval:** European Patent Publication No. 0 244 944 (24th January 1990)	
1990	Amlodipine besylate launched by Pfizer in the UK as Istin	**First non-research use in patients AND Reimbursement/financial support**	**Publication:** IMS dataset	
31 July 1992	US regulatory approval of Norvasc	**Regulatory approval**	**Regulatory approval:** NDA 19-787	FDA NDA applications and approvals are available in the Orange Book (electronic online edition)
November 1992	Amlodipine besylate launched by Pfizer in the US as Norvasc	**First non-research use in patients**		Subscription to IMS data required
1990 –	A prospective randomized survival evaluation study established the safety of amlodipine for the treatment of angina or hypertension in patients with advanced left ventricular dysfunction	**Effectiveness/ post-launch research**	**Publication:** Packer et al. (1996)	Web search using appropriate filters in: PubMed and Cochrane Library

1990 –	A base-case analysis showed a slightly higher percentage of patients achieve hypertension control with amlodipine but at a substantially higher cost than with a generic diuretic (chlorthalidone)	**Effectiveness/ post-launch research**	**Publication:** Ramsey et al. (1999)	Web search using appropriate filters in: PubMed and Cochrane Library
1990 –	Retrospective literature analysis showed amlodipine had a better duration of action compared to other calcium antagonists	**Research review & synthesis on effectiveness and safety**	**Publication:** Zannad et al. (1996)	Web search using appropriate filters in: PubMed and Cochrane Library
1990 –	Recommendation of amlodipine (and felodipine) over other calcium antagonists in patients with left ventricular dysfunction	**National policy announcement/ guidelines/ advice**	**Publication:** NIH (1997)	Web search using appropriate filters in: PubMed and Cochrane Library
2001	NICE Clinical Guideline recommending two calcium channel blockers (verapamil and diltiazem) as last line treatment in patients with prior myocardial infarction who do not have heart failure. Other calcium channel blockers (including amlodipine?) should be considered only subsequently	**National policy announcement/ guidelines/ advice**	**Publication:** NICE (2001)	
2002	ALLHAT trial compared ACE inhibitor, CCBs and diuretic in high-risk patients and concludes that diuretics are superior in preventing CVD and less expensive of other interventions, so they should be preferred for first-step antihypertensive therapy	**Human research: Effectiveness/ Post-launch research**	**Publication:** ALLHAT (2002)	
July 2003	NICE Clinical Guideline (CG05):	**National policy**	**Publication:** NICE (2003)	

	"Amlodipine should be considered for the treatment of co-morbid hypertension and/or angina in patients with heart failure, but verapamil, diltiazem or short-acting Dihydropyridine agents should be avoided". This recommendation is based on what NICE defines "good evidence"	**announcement/ guidelines/ advice**		
March 2004	Generic amlodipine is launched in the UK		**Publication:** IMS data	We do not have a track to define this event, but it can be important in cost-effectiveness/reimbursement decisions
August 2004	NICE Clinical Guideline (CG018) recommended calcium channel blockers (including amlodipine) as a third line therapy for hypertension	**National policy announcement/ guidelines/ advice**	**Publication:** NICE (2004)	
2004	VALUE trial reported that amlodipine has more pronounced effects than valsartan on reducing blood pressure	**Human research: Effectiveness/ Post-launch research**	**Publication:** Julius et al. (2004)	
2005	ASCOT trial found that amlodipine-based regimen prevents more major cardiovascular events and induces less diabetes than atenolol-based regimen	**Human research: Effectiveness/ Post-launch research**	**Publication:** Dahlof et al. (2005)	
2006	Rapid update NICE guideline recommended a calcium-channel blocker or a thiazide-type diuretic as first-line treatment in hypertensive patients 55 or over, or black patients of any age	**National policy announcement/ guidelines/ advice**	**Publication:** NICE (2006)	

Issues arisen in developing the timeline:

- [applicable to both amlodipine and olanzapine] If we keep both the "first non-research use in patients" and the "reimbursement/financial support" tracks this necessitates duplication of launch calibration point whenever we look at pre-NICE medicines

- [applicable to both amlodipine and olanzapine] For pharmaceuticals, the "Intervention becomes standard of practice" could be questionable, as it is not clear how one can distinguish it from the "National policy announcement/ guidelines / advice" track

- [applicable to both amlodipine and olanzapine] In some cases it was not possible to find the publication of a study/event and the citation in a publication about the study/event was used as calibration point instead. This can possibly introduce a bias in time lag estimation as the citation may occur several years after the event

- [applicable to both amlodipine and olanzapine] Information about initial drug development (phase I and II clinical trials) is usually confidential. And, especially for "old" drugs, there are no online available public registry of trials

- [applicable to both amlodipine and olanzapine] the US patents are relatively easy to retrieve, while there is no online available register for the UK patents

- During a project workshop in January 2013 we agreed to consider also the approval date of the first generation calcium channel blocker (amlodipine is a third generation calcium channel blocker) to show the previous stages of research. Here I am considering the approval of the first generation calcium channel blocker in the Discovery track, however this might be incorrect and it might be the case where we should not consider it in the amlodipine timeline

- When the potential publication of a study/event is not available, it is not possible to confirm that that publication actually refers to the study/event (e.g. first study in humans), so it cannot be stated for sure that the publication is a calibration point for the study/event

Generic amlodipine was launched in the UK in March 2004. We do not have a track to capture this event, however it is an important event to take into account as it could have had a relevant impact on NICE cost-effectiveness and recommendation decisions.

Matrix: **Amlodipine: (approximately) 50 years from the beginning of research to the NICE recommendation**

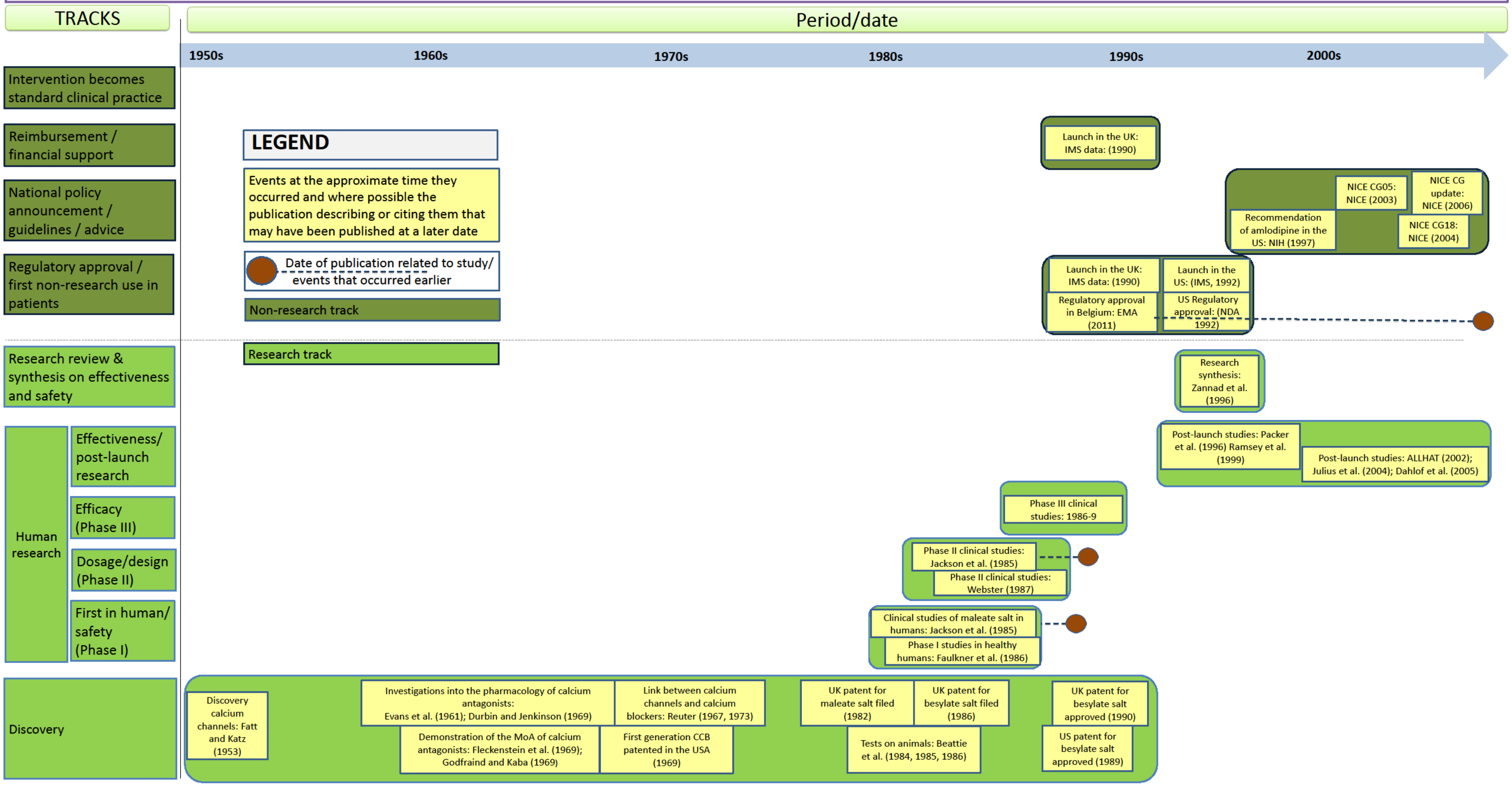

METHODOLOGICAL REFLECTIONS

This case study was conducted researching the online available information (both in published journals and in the grey literature) covering all the possible calibration points throughout the different tracks of the drug history, from early research to NICE clinical guidelines. This approach requires a deep knowledge of a drug research, development, launch and post-launch processes to identify the main events to be recorded; and involves both backward and forward tracing from the different calibration points. Valuable sources of information (which cannot be expected to be available in every drug case study) are the court cases, which reconstruct in detail the events related to the R&D of a drug.

There are advantages and disadvantages related to this approach. The main advantage is that this approach allows covering all the key stages of a drug history as it also considers the grey literature, which is a valuable (and sometimes the only) source of information for a drug commercial history (patents, regulatory application and approval, launch, national policy recommendation, uptake). When applied to more recent technologies, this approach potentially allows gathering more information, as it should be expected that drugs/interventions researched and developed in the Internet era have an extensive amount of records available online. One disadvantage of this approach is that it cannot be automated, as it requires seeking specific information manually. However, there are also economies of learning due to the recurrence of the main sources of information (e.g. the US Patent and Trademark Office or the FDA “Orange Book”).

Some observations can also be made about the information availability and validity. In general, clinical studies at the earlier stages of a drug development (phase I and II) are not usually published in peer-reviewed journals or considered in research synthesis publications (an exception is represented by “more popular” drugs or drugs whose patent has been disputed in a court case). Moreover, how far peer-review publications record the start date of clinical studies varies, and practice is evolving. Furthermore ,the date of publication of the study may differ significantly from the date when the study took place. Furthermore, online datasets of clinical trials (e.g. clinicaltrials.gov) are relatively recent and therefore contain little information relevant to older drugs.

It should also be noticed that many different and staggered patents can be associated with a single drug. In this case, interesting information can be obtained investigating why different patents were granted. For instance, in the amlodipine case the new patent on the besylate salt testifies how the drug development can be complicated (and possibly delayed) because of formulation problems due to the stability of the salt (and not because the compound does not work).

It is also important to remember that NICE was set up in 1999. Therefore any measurement of lapsed time involving the publication date of a NICE guideline as calibration point should be considered as illustrative only if referred to interventions available before 1999.

REFERENCES

The Antihypertensive and Lipid-Lowering Treatment to Prevent Heart Attack Trial (ALLLHAT, 2002). Major outcomes in high-risk hypertensive patients randomized to angiotensin-converting enzyme inhibitor or calcium channel blocker vs diuretic. *JAMA,* 288:2981-2997.

Beattie, D. T., Cunnane, T. C., & Muir, T. C. (1984). Effects of Ca^{2+}-entry blockers on conduction in pre-terminal nerves in the guinea-pig vas deferens. *Proceedings JUPHAR 9th International Congress of Pharmacology, London, 1643P*.

Beattie, D. T., Cunnane, T. C., & Muir, T. C. (1985). Effects of Ca^{2+}-entry blockers on spontaneous and electrically-evoked release of transmitter from guinea-pig vas deferens nerves. *British journal of pharmacology*, *84*, 29P.

Beattie, D.T., Cunnane, T.C., & Muir, T. C. (1986). Effects of calcium channel antagonists on action potential conduction and transmitter release in the guinea-pig vas deferens. *British journal of pharmacology*, *89*(1), 235-244.

Buxton, M.J. et al. (2008) *Medical Research: What's it Worth?* London: Academy of Medical Sciences, Medical Research Council, Wellcome Trust.

Dahlof, B. et al. (2005). Prevention of cardiovascular events with an antihypertensive regimen of amlodipine adding perindopril as required versus atenolol adding bendroflumethiazide as required, in the Anglo-Scandinavian Cardiac Outcomes Trial-Blood Pressure Lowering Arm (ASCOT-BPLA): a multicentre randomised controlled trial. *The Lancet*, 366:895-906.

EMA (2011). *Assessment report pursuant to Article 30 of Directive 2001/83/EC, as amended*. Procedure No: EMEA/H/A-30/1288.

European Patent Office (EPO, 1986). Patent Publication No. 0 089 167.

European Patent Office (EPO, 1990). Patent Publication No. 0 244 944.

Evans, D. H. L., Schild, H. O., & Thesleff, S. (1958). Effects of drugs on depolarized plain muscle. *The Journal of physiology*, *143*(3), 474-485.

Durbin, R. P., & Jenkinson, D. H. (1961). The calcium dependence of tension development in depolarized smooth muscle. *The Journal of physiology*, *157*(1), 90-96.

Fatt, P., & Katz, B. (1953). The electrical properties of crustacean muscle fibres. *The Journal of physiology*, *120*(1-2), 171-204.

Faulkner, J. K., McGibney, D., Chasseaud, L. F., Perry, J. L., & Taylor, I. W. (1986). The pharmacokinetics of amlodipine in healthy volunteers after single intravenous and oral doses and after 14 repeated oral doses given once daily. *British journal of clinical pharmacology*, *22*(1), 21-25.

Fleckenstein, A., Tritthart, H., Flackenstein, B., Herbst, A., & Grün, G. (1969). A new group of competitive divalent Ca-antagonists (iproveratril, D 600, prenylamine) with potent inhibitory effects on electromechanical coupling in mammalian myocardium]. *Pflügers Archiv: European journal of physiology*,*307*(2), R25.

Food and Drug Administration (FDA, 1989). New drug application NDA 19-684.

Food and Drug Administration (FDA, 1992). New drug application NDA 19-787.

Godfraind, T., & Kaba, A. (1969). Inhibition by cinnarizine and chlorpromazine of the contraction induced by calcium and adrenaline in vascular smooth muscle. *British journal of pharmacology*, *35*(2), P354.

Godfraind, T. (2004). *Calcium channel blockers*. Birkhäuser.

Jackson, N. C., Lee, P. S., Reynolds, G., Frais, M., & Taylor, S. H. (1985). Improvement of treadmill time to angina by amlodipine. *Br J Clin Pharmacol*, *20*, 248.

Julius, S. et al. (2004). Outcomes in hypertensive patients at high cardiovascular risk treated with regimens based on valsartan or amlodipine: the VALUE randomised trial. *The Lancet*, 363:2022-31.

National Institute for Clinical Excellence, North of England Evidence-based Guidelines Development Project (NICE, 2001). *Prophylaxis for patients who have experienced a myocardial infarction: drug treatment, cardiac rehabilitation and dietary manipulation - guideline.* London: National Institute for Clinical Excellence (NICE) 2001: 115

NICE: National Collaborating Centre for Chronic Conditions (NICE, 2003). *Chronic heart failure - Management of chronic heart failure in adults in primary and secondary care*. London.

National Institute for Clinical Excellence, North of England Hypertension Guideline Development Group (NICE, 2004). *Essential hypertension: managing adult patients in primary care*. NICE Clinical Guideline 18.

NICE: National Collaborating Centre for Chronic Conditions (NICE, 2006). *Hypertension: management in primary care: pharmacological update*. London: Royal College of Physicians.

National Institutes of Health, NIH (1997). *The Sixth report of the Joint National Committee on prevention, detection, evaluation, and treatment of high blood pressure.* NIH Publication No. 98-4080.

Packer, M., O'Connor, C. M., Ghali, J. K., Pressler, M. L., Carson, P. E., Belkin, R. N., ... & DeMets, D. L. (1996). Effect of amlodipine on morbidity and mortality in severe chronic heart failure. *New England Journal of Medicine*,*335*(15), 1107-1114.

Ramsey, S. D., Neil, N., Sullivan, S. D., & Perfetto, E. (1999). An economic evaluation of the JNC hypertension guidelines using data from a randomized controlled trial. Joint National Committee. *The Journal of the American Board of Family Practice*, *12*(2), 105-114.

Reuter, H. (1967). The dependence of slow inward current in Purkinje fibres on the extracellular calcium-concentration. *The Journal of physiology*, *192*(2), 479-492.

Reuter, H. (1973). Divalent cations as charge carriers in excitable membranes. *Progress in biophysics and molecular biology*, *26*, 1.

Tsien RW, Barrett CF. A Brief History of Calcium Channel Discovery. In: Madame Curie Bioscience Database [Internet]. Austin (TX): Landes Bioscience; 2000-. Available from: http://www.ncbi.nlm.nih.gov/books/NBK6465/

Webster, J., Robb, O. J., Jeffers, T. A., Scott, A. K., Petrie, J. C., & Towler, H. M. (1987). Once daily amlodipine in the treatment of mild to moderate hypertension. *British journal of clinical pharmacology*, *24*(6), 713-719.

UK Intellectual Property Office (UK IPO, 1982). Patent application GB8207180.

UK Intellectual Property Office (UK IPO, 198?). Patent No. 160833.

United States District Court For The Western District of Pennsylvania (FFCL223, 2007). Pfizer Inc. vs. Mylan Laboratories Inc. and Mylan Pharmaceuticals Inc. Findings of Fact and Conclusions of Law No. 223 ordered on 27 February 2007.

US Patent and Trademark Office (US PTO, 1969). US Patent Number 3,485,847.

US Patent and Trademark Office (US PTO, 1986). US Patent Number 4,572,909.

US Patent and Trademark Office (US PTO, 1989). US Patent Number 4,879,303.

Zannad, F., Matzinger, A., & Larché, J. (1996). Through/peak ratios of once daily angiotensin converting enzyme inhibitors and calcium antagonists. *American journal of hypertension*, *9*(7), 633-643.

Time lags between conducting medical research and its translation. Case Study 2: Olanzapine

NARRATIVE ACCOUNT OF BACKGROUND/ DEFINITIONS AND KEY ASPECTS OF THE INTERVENTION'S DEVELOPMENT

Background/definition

Schizophrenia and olanzapine

Schizophrenia is a chronic, debilitating mental illness that appears during late adolescence or early adulthood and essentially lasts the lifetime of the patient. It is the most common major mental disorder in the UK, and directly affects one in 100 people at some point during their lives.

Scientists divide the symptoms of schizophrenia, which include delusions, abnormal thoughts and behaviour and lethargy, into 'positive' and 'negative'. The positive symptoms occur in the acute phase of the condition and include restless, noisy and irrational behaviour, sudden mood changes, inappropriate moods, hallucinations – often hearing voices – delusions and disordered thinking. The negative symptoms include tiredness, social withdrawal, physical slowness, lack of activity and interest in things and self-neglect, and usually occur in the chronic stage of schizophrenia.[3]

Olanzapine is an atypical antipsychotic drug for the treatment of schizophrenia and bipolar disorder. It was originally sold by Eli Lilly and Company under the brand name Zyprexa® but today available generically. Olanzapine relieves the symptoms of schizophrenia (hearing, seeing, or sensing things that are not real, having mistaken beliefs, and feeling unusually suspicious) by correcting the imbalance of chemical substances which act on the nervous system in the brain.

Early Drug Treatment – Clozapine

Clozapine, the first atypical antipsychotic drug treating schizophrenia, was first marketed by Sandoz in Switzerland and Austria in 1972, in West Germany in 1974 and in Finland in 1975.[4] Prior to its launch, the only available drugs were typical antipsychotics, which only dealt with the positive symptoms of schizophrenia and had considerable side effects (acute extrapyramidal symptoms, EPS, were reported in 38.9% of patients).[5] Clozapine was considered a breakthrough innovation because it could treat both positive and negative symptoms of schizophrenia with no EPS.

However, it was soon found that a life-threatening blood disorder (agranulocytosis) was associated with clozapine treatment and the manufacturer voluntarily withdrawn the drug from the market in 1975.[6] This prompted pharmaceutical companies to start looking for a drug like clozapine, but without the same side effects.

---

[3] Information available in the Medical Research Council website (consulted on 16 January 2013): http://www.mrc.ac.uk/Achievementsimpact/Storiesofimpact/Schizophrenia/index.htm

[4] A complete review of the history of clozapine is provided by: Crilly, John. "The history of clozapine and its emergence in the US market a review and analysis." *History of psychiatry* 18, no. 1 (2007): 39-60.

[5] Shen, Winston W. "A history of antipsychotic drug development." *Comprehensive psychiatry* 40, no. 6 (1999): 407-414.

[6] Lehmann, Heinz E., and Thomas A. Ban. "The history of the psychopharmacology of schizophrenia." *Canadian journal of psychiatry* 42, no. 2 (1997): 152-162.

Research and Development of Olanzapine

Preliminary studies

Beginning in the autumn of 1974, Lilly UK started to research an atypical antipsychotic drug at its Erl Wood research facility.[7] The research team was led by research scientist Dr Jiban Chakrabarti, who first suspected that by altering the clozapine structure it would be possible to create a safer medicine. Research scientist Dr David Tupper and research chemist Dr Terry Hotten, who were working with Dr Chakrabarti, produced a novel class of compounds (thienobenzodiazepines), but only a few appeared to have an effect on psychoses.[8]

In 1975, Lilly filed a patent application related to these compounds. The patent was issued by the US Patent and Trademark Office in 1978.[9] This patent led to a dispute in 1991 when the olanzapine US patent claim was filed.

Lilly spent the next four years developing one lead compound (flumezapine) through preclinical testing and initial safety testing in healthy human volunteers.[10] The first trial in actual schizophrenic patients started in the spring of 1982 but the first reports shown high toxicity level and the development of flumezapine was terminated.

After this failure, a team lead by Dr Hotten made and tested several alternatives, including the compound known as olanzapine, which was first synthesised in the UK on 29 April 1982.[11]

Study in cell lines or animals (pre-clinical)

Lilly started testing olanzapine in beagles in 1983. The first study lasted three months, a one-year study followed later.[12] The tests indicated that olanzapine was sufficiently safe to be tested in human trials.

Phase 1 clinical trials

Phase 1 clinical trials in healthy human volunteers were conducted in 1986 and 1987 in Indianapolis, Indiana.[13]

Phase 2 clinical trials

Clinical trials to test efficacy in actual patients took place in the UK and toward the end of 1989 demonstrated favourable results.[14] On 18 January 1990, the Research Management Staff agreed to "product commitment".[15]

---

[7] Information available online (page consulted on 13 January 2013): http://www.ehs.lilly.com/1997/zyprexa/compound.htm

[8] Information available online (page consulted on 13 January 2013): http://www.innovation.org/index.cfm/StoriesofInnovation/InnovatorStories/The_Story_of_Zyprexa?popwindow

[9] United States District Court Southern District of Indiana, Indianapolis Division. Eli Lilly and Company and Lilly Industries ltd. vs. Zenith Goldline Pharmaceuticals, Inc., Dr. Reddy's Laboratories, ltd., and Teva Pharmaceuticals USA, Inc. Findings of Fact and Conclusions of Law No. 443 (hereinafter FFCL443) ordered on 14 April 2005. Par. 26.

[10] FFCL443, par. 36.

[11] FFCL443, par. 43.

[12] FFCL443, par. 45.

[13] FFCL443, par. 46.

[14] FFCL443, par. 47.

Patent application

On 25 April 1990, Lilly filed a patent application in the UK. The US patent application was filed on 23 April 1991.[16] The US patent application was rejected on 23 November 1991 declaring olanzapine unpatentable because it represented an obvious homologous substituent to the compound class patented in 1978 ("obviousness-type double patenting").[17]

Lilly responded to the rejection on 10 December 1992[18] arguing that *olanzapine displayed an unexpected and significant superior toxicological benefit over* the compound patented in 1978.[19] This claim was accepted on 17 December 1992 and the US Patent and Trademark Office issued olanzapine patent on 20 July 1993.[20]

Phase 3 clinical trials

In November 1993, Lilly obtained the results from the first pivotal clinical trial involving Zyprexa™ (LY170053, olanzapine), which demonstrated superior efficacy and safety to the existing therapies.[21] There have been four randomised double-blind clinical trials of olanzapine for treatment of schizophrenia prior to license.[22]

Regulatory approval from FDA (US), EMA (EU), and/or MHRA (UK)

Although olanzapine was superior to the existing primary and secondary treatments for schizophrenia, Lilly acknowledged that several competitors had compounds in advanced stages of clinical trials. For this reason, Lilly developed a registration strategy which enable it to submit dossiers in 21 countries within several days of one another.

Nine months prior to the scheduled submission date (1 October 1995), the planning phase culminated with a global registration strategy.[23] The NDA and the European Marketing Authorization Application were submitted on the same day (22 September 1995) to United States FDA and the EMEA (now EMA) for the EU, respectively. Submissions were made to Australia, Canada, New Zealand, Norway, and Switzerland in the ensuing days.[24]

---

[15] FFCL443, par. 48.
[16] FFCL443, par. 53-54.
[17] FFCL443, par. 62-66.
[18] FFCL443, par. 70.
[19] FFCL443, par. 74.
[20] FFCL443, par. 91.
[21] Worthen, Sue T., Jeffrey S. Kasher, John Saunders, Diana L. Mckenzie, Robert E. Hizer, Susanne Acklin, and Jeffrey T. Ramsey. "The Global Registration of Zyprexa™(Olanzapine)." *Drug information journal* 31, no. 1 (1997): 49-55.
[22] Cummins, Carole, Andrew Stevens, and Steven Kisely. *The use of olanzapine as a first and second choice treatment in schizophrenia*. NHS Executive South & West R&D Directorate, 1998.
[23] Worthen, Sue T., Jeffrey S. Kasher, John Saunders, Diana L. Mckenzie, Robert E. Hizer, Susanne Acklin, and Jeffrey T. Ramsey. "The Global Registration of Zyprexa™(Olanzapine)." *Drug information journal* 31, no. 1 (1997): 49-55.
[24] Kaitin, Kenneth I., and Elaine M. Healy. "The New Drug Approvals of 1996, 1997, and 1998: Drug Development Trends in the User Fee Era." *Drug Information Journal* 34, no. 1 (2000): 1-14.

EMEA approval was granted on 27 September 1996 and FDA approval on 30 September 1996.[25] It was launched first on October 1996 in 14 countries (including the UK) and then progressively in more countries over the next 21 months.[26]

Uptake in the market

The following graph shows uptake of olanzapine in primary care in England, measured in prescription items, following its launch in the UK in October 1996.

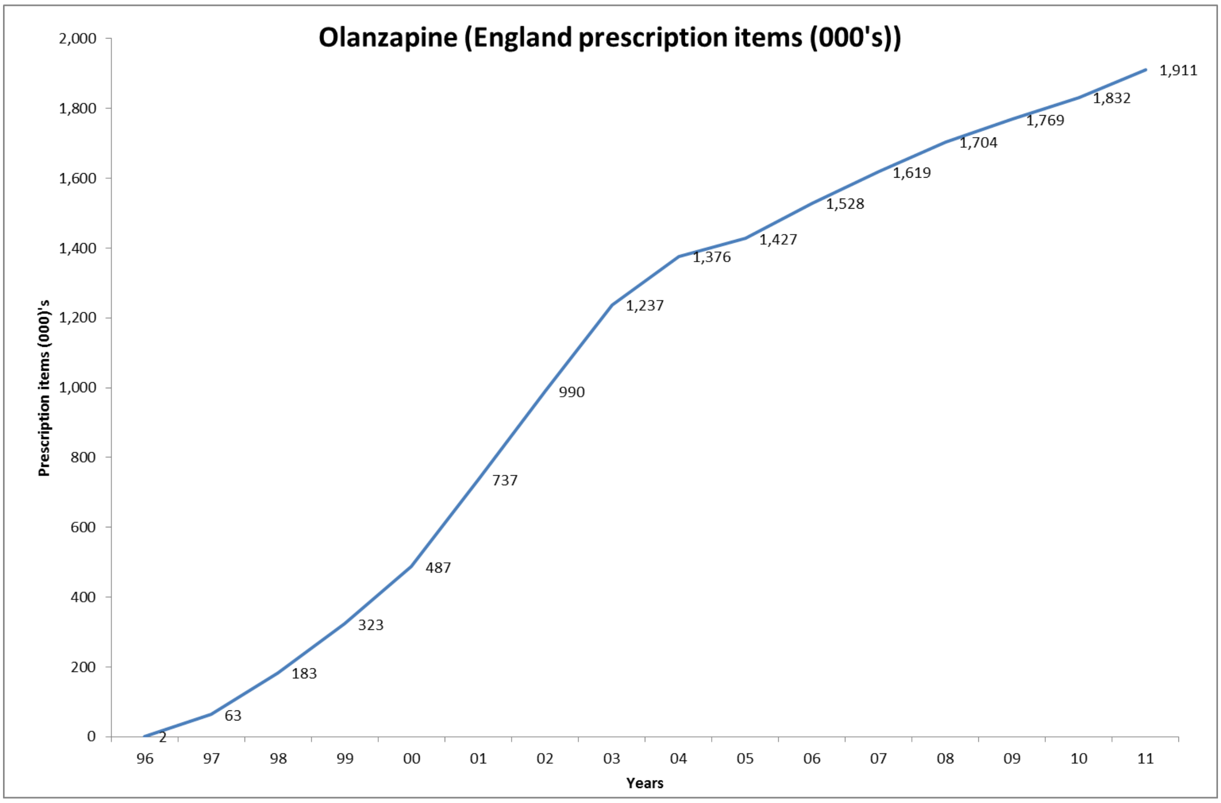

Source: author elaboration from Prescription Cost Analysis – England data.

Research Synthesis & clinical policy and practice to encourage uptake

In the UK, NICE recommended olanzapine (and other four new atypical antipsychotic drugs) as one of the first line options for schizophrenia (6 June 2002).[27] This made the NHS legally obliged to provide funding and resources for the reimbursement of olanzapine when prescribed.

[25] Information available online (page consulted on 13 January 2013): http://www.ehs.lilly.com/1997/zyprexa/launch.htm

[26] Applbaum, Kalman. "Getting to yes: corporate power and the creation of a psychopharmaceutical blockbuster." *Culture, medicine and psychiatry* 33, no. 2 (2009): 185-215.

[27] NICE historical archive.

TIMELINE

NB: blue denotes events related to the bipolar disorder indication

Period/date	Study(ies)/Event	Track	Calibration point	Comments
Fall 1974	Lilly UK started to research an atypical antipsychotic drug at its Erl Wood research facility	**Discovery**		Information available online:<br>Olanzapine Development Timeline<br>Story of Zyprexa
1975- early 1980s	Lilly produced a novel class of compounds called thienobenzodiazepines	**Discovery**		This information is provided in the US Court Case FFCL443, par. 25
26 November 1974	UK patent application filed	**Discovery**	**Patent filing:** GB51240/74	
20 November 1975	Lilly filed a patent application in the US related to the thienobenzodiazepines compounds class	**Discovery**	**Patent filing:** US application Serial No. 633,895 (abandoned)	
1976	First compound (ethyl flumezapine) to progress to toxicological testing	**Discovery**		This information is provided in the US Court Case FFCL443, par. 33
1 June 1977	US patent continuation	**Discovery**	**Patent filing:** US application Serial No. 802,381	
19 September 1978	Patent issued by the US Patent and Trademark Office	**Discovery**	**Patent approval:** US Patent Number: 4,115,574	
1978	Development of ethyl flumezapine discontinued due to high toxicity in dogs	**Discovery**		This information is provided in the US Court Case FFCL443, par. 35
early 1978	Commenced the development of a different compound (flumezapine, compound LY120363) under the guidance of Dr Ian Pullar	**Discovery**		This information is provided in the US Court Case FFCL443, par. 35
spring 1982	Flumezapine first trial in actual schizophrenic patients started and terminated due to high	**Discovery**		This information is provided in the US Court Case

	toxicity			FFCL443, par. 38
29 April 1982	Olanzapine first synthesised in the UK	**Discovery**	**Cited in:** FFCL443 (2005)	This information is provided in the US Court Case FFCL443, par. 43
1983	Lilly started testing olanzapine in beagles (first in a three-month study and later in a one-year study)	**Discovery**	**Cited in:** FFCL443 (2005)	This information is provided in the US Court Case FFCL443, par. 45
	In vivo animal studies suggested that olanzapine might have the profile of an atypical antipsychotic in the treatment of schizophrenia	**Discovery**	**Publications**: Fuller and Snoddy (1992); Moore et al. (1992)<br>**Cited in:** Nyberg et al. (1997)	
24 July 1985	Investigational New Drug (IND) Application in the USA		**Cited in:** FDA approval (1995)	
1986-1987	Phase 1 clinical trials conducted in healthy human volunteers in Indianapolis (Indiana)	**Human research: first in human/safety (Phase I)**	**Possible citation in:** Nyberg et al. (1997)	This information is provided in the US Court Case FFCL443, par. 46
December 1988	The first patient was recruited into a Phase II clinical trial which started in December 1988 and took place in St Mary's Hospital (London).	**Human research: dosage/design (Phase II)**	**Publication:** Baldwin and Montgomery (1995)	Baldwin, personal communication
end of 1989	A clinical trial to test efficacy in actual patients that took place in St Mary's Hospital (London) demonstrated favourable results	**Human research: dosage/design (Phase II)**	**Publication:** Baldwin and Montgomery (1995)	This information is provided in the US Court Case FFCL443, par. 47
18 January 1990	Lilly Research Management Staff agreed to product commitment			This information is provided in the US Court Case FFCL443, par. 48
25 April 1990	Patent filed in the UK	**Discovery**	**Patent filing:** UK Application GB9009229	
23 April 1991	US patent application	**Discovery**	**Patent filing:** Application Serial No. 690,143	

			(abandoned)	
23 November 1991	US patent application rejected because of "obviousness-type double patenting"	**Discovery**		
22 May 1992	US patent continuation filed	**Discovery**	**Patent filing:** Application Serial No. 890,348	
10 December 1992	Lilly responded to the US patent rejection	**Discovery**		
17 December 1992	Lilly claim on patent rejection was accepted	**Discovery**		
20 July 1993	The US Patent and Trademark Office issued olanzapine patent	**Discovery**	**Patent approval:** US Patent Number: 5,229,382	
Prior to 1995	The pharmacodynamic profile of olanzapine has been explored with PET (test in 3 human volunteers – effective dosage)	**Human research: dosage/design (Phase II)**	**Cited in:** Nyberg et al. (1997)	
October 1991 – 3 August 1994	Two placebo-controlled trials (F1D-EW-E003 and F1D-MC-HGAD) have established the efficacy of olanzapine (7.5 to 17.5 mg/day) in the treatment of an acute exacerbation of chronic DSM-IIIR-compatible schizophrenia	**Human research: dosage/design (Phase II)**	**Cited in:** Tolleffson et al. (1995)<br>**Possible citation in:** Satterlee et al. (1995); Baker et al. (1995)	
?	A third multinational trial suggested an increasing dose-response	**Human research: dosage/design (Phase II)**	**Cited in:** Tolleffson et al. (1995)<br>**Possible citation in:** Dittman et al. (1995)	
June 1993 – 14 February 1994	A single global Phase III protocol was conducted in 18 countries involving 178 investigative sites. Over 1996 patients were randomized to either olanzapine (5 to 20 mg) or haloperidol (5 to 20	**Human research: efficacy (Phase III)**	**Cited in:** Tolleffson et al. (1995)<br>**Publication:** Beasley et al.	

	mg) (Study F1D-MC-HGAJ).		(1997)	
November 1993	First results from the first pivotal clinical trial involving olanzapine (Study F1D-MC-HGAJ)	**Human research: efficacy (Phase III)**	**Cited in:** Worthen et al. (1997)	
1992-1997	Four double-blind pivotal studies, which compare olanzapine to placebo and/or haloperidol, are presented. The results suggest that olanzapine is as effective as haloperidol for positive symptoms and more effective than haloperidol for the treatment of the negative symptoms of schizophrenia.	**Human research: efficacy (Phase III)**	**Cited in:** Beasley, Tollefson and Tran (1997).<br>**Cited in:** Cummins et al. (1998)	
6-12 April 1995	Results from Phase III clinical studies presented at "The V International Congress on Schizophrenia Research", Wormsprings, VA USA	**Human research: efficacy (Phase III)**	**Cited in:** Tran et al. (1995) (Cochrane Central Register of Controlled Trials)	
14 June 1995	First study in actual patients submitted for publication	**Human research: dosage/design (Phase II)**	**Publication:** Baldwin and Montgomery (1995)	
22 September 1995	Standard NDA application submitted (NDA 20-592)	**Regulatory approval / first non-research use in patients**	**Cited in Regulatory Approval:** NDA 20-592 (1996)<br>**Cited:** Kaitin and Healy (2000)	
22 September 1995	EMEA (now EMA) application submitted	**Regulatory approval / first non-research use in patients**	**Cited in:** Kaitin and Healy (2000)<br>**Cited in Regulatory Approval:** EMA (2004)	
November 1995	Publication of the results from the first clinical study in patients with schizophrenia	**Human research: dosage/design (Phase II)**	**Publication:** Baldwin and Montgomery (1995)	
27 September 1996	EMEA (now EMA) approval; CPMP (now CHMP) issued a marketing authorisation 0646/96 valid throughout the European Union	**Regulatory approval / first non-research use in patients**	**Cited in:** Kaitin and Healy (2000)<br>**Cited in:** EMA (2004)	

30 September 1996	FDA approval	**Regulatory approval / first non-research use in patients**	**Regulatory Approval:** NDA 20-592 (1996)<br>**Cited in:** Kaitin and Healy (2000)	
18 October 1996	Begin of study F1D-MC-HGEH comparing olanzapine vs. placebo in the treatment of mania associated with bipolar I disorder	**Human research: efficacy (Phase III)**	**Cited in:** LillyTrials (2004)	
October 1996	US launch	**Regulatory approval / first non-research use in patients**	**Cited in:** Kaitin and Healy (2000)<br>**Possible citation in:** IMS Data	
October 1996	UK launch	**Regulatory approval / first non-research use in patients**	**Cited in:** Kaitin and Healy (2000)<br>**Citation in:** IMS Data	
22 August 1997	Conclusion of study F1D-MC-HGEH comparing olanzapine vs. placebo in the treatment of mania associated with bipolar I disorder	**Human research: efficacy (Phase III)**	**Cited in:** LillyTrials (2004)	
September 1997	Begin of study F1D-MC-HGFU analysing olanzapine added to mood stabiliser in the treatment of bipolar disorder	**Human research: efficacy (Phase III)**	**Cited in:** LillyTrials (2004)	
2 May 1998	Study F1D-US-HGGD (HGGD) to compare 1-year cost effectiveness of initial treatment with olanzapine versus a fail-first algorithm on conventional antipsychotics (CON)	**Effectiveness / post-launch research**	**Cited in:** LillyTrials (2004)	
1998	West Midlands Development and Evaluation Committee Report declared olanzapine more cost-effective over haloperidol as first and second choice therapy for schizophrenia	**Research review & synthesis on effectiveness and safety**	**Publication:** Cummins et al. (1998)	

December 1998	Begin of study F1D-MC-HGDG comparing olanzapine vs. placebo in the treatment of bipolar disorder, manic or mixed	**Human research: efficacy (Phase III)**	**Cited in:** LillyTrials (2004)	
1999	Comparison between atypical antipsychotics	**Effectiveness / post-launch research**	**Cited in:** Soares et al. (1999)	
September 2000	Conclusion of study F1D-MC-HGDG comparing olanzapine vs. placebo in the treatment of bipolar disorder, manic or mixed	**Human research: efficacy (Phase III)**	**Cited in:** LillyTrials (2004)	
October 2000	Conclusion of study F1D-MC-HGFU analysing olanzapine added to mood stabiliser in the treatment of bipolar disorder	**Human research: efficacy (Phase III)**	**Cited in:** LillyTrials (2004)	
14 March 2001	Application to EMA marketing authorisation to license olanzapine for the treatment of bipolar disorder	**Regulatory approval / first non-research use in patients**	**Publication:** EMA (2004)	
6 September 2002	Study F1D-US-HGGD (HGGD) to compare 1-year cost effectiveness of initial treatment with olanzapine versus a fail-first algorithm on conventional antipsychotics (CON)	**Effectiveness / post-launch research**	**Cited in:** LillyTrials (2004)	
6 June 2002	NICE recommended olanzapine as one of the first line options for schizophrenia	**National policy / announcement / guidelines / advice**	**Publication:** NICE Clinical Guideline 1 (2002)	
June 2002	EMA grants the marketing authorisation to use olanzapine for the treatment of bipolar disorder	**Regulatory approval / first non-research use in patients**	**Cited in:** EMA (2004)	
2003	More than 1,000,000 prescriptions in England (1,237,000 prescriptions)		**Publication:** NHS Information Centre	We have all statistics on prescription in England between 1996 (launch) and 2011. We did not include this event into the matrix as a calibration point for the "Intervention becomes standard of practice" track because it would be too arbitrary.

24 September 2003	NICE issued guidance to the NHS in England and Wales on the use of olanzapine and valproate semisodium in the treatment of acute mania associated with bipolar disorder (technology appraisal TA66)	**Reimbursement / financial support**	**Publication:** NICE (2003)	

Issues arisen in developing the timeline:

- [applicable to both amlodipine and olanzapine] If we keep both the "first non-research use in patients" and the "reimbursement/financial support" tracks this necessitates duplication of launch calibration point whenever we look at pre-NICE medicines
- [applicable to both amlodipine and olanzapine] For pharmaceuticals, the "Intervention becomes standard of practice" could be questionable, as it is not clear how one can distinguish it from the "National policy announcement/ guidelines / advice" track
- [applicable to both amlodipine and olanzapine] In some cases it was not possible to find the publication of a study/event and the citation in a publication about the study/event was used as calibration point instead. This can possibly introduce a bias in time lag estimation as the citation may occur several years after the event
- [applicable to both amlodipine and olanzapine] Information about initial drug development (phase I and II clinical trials) is usually confidential. And, especially for "old" drugs, there are no online available public registry of trials
- [applicable to both amlodipine and olanzapine] the US patents are relatively easy to retrieve, while there is no online available register for the UK patents

Matrix: **Olanzapine: (approximately) 28 years from the beginning of research to the NICE recommendation**

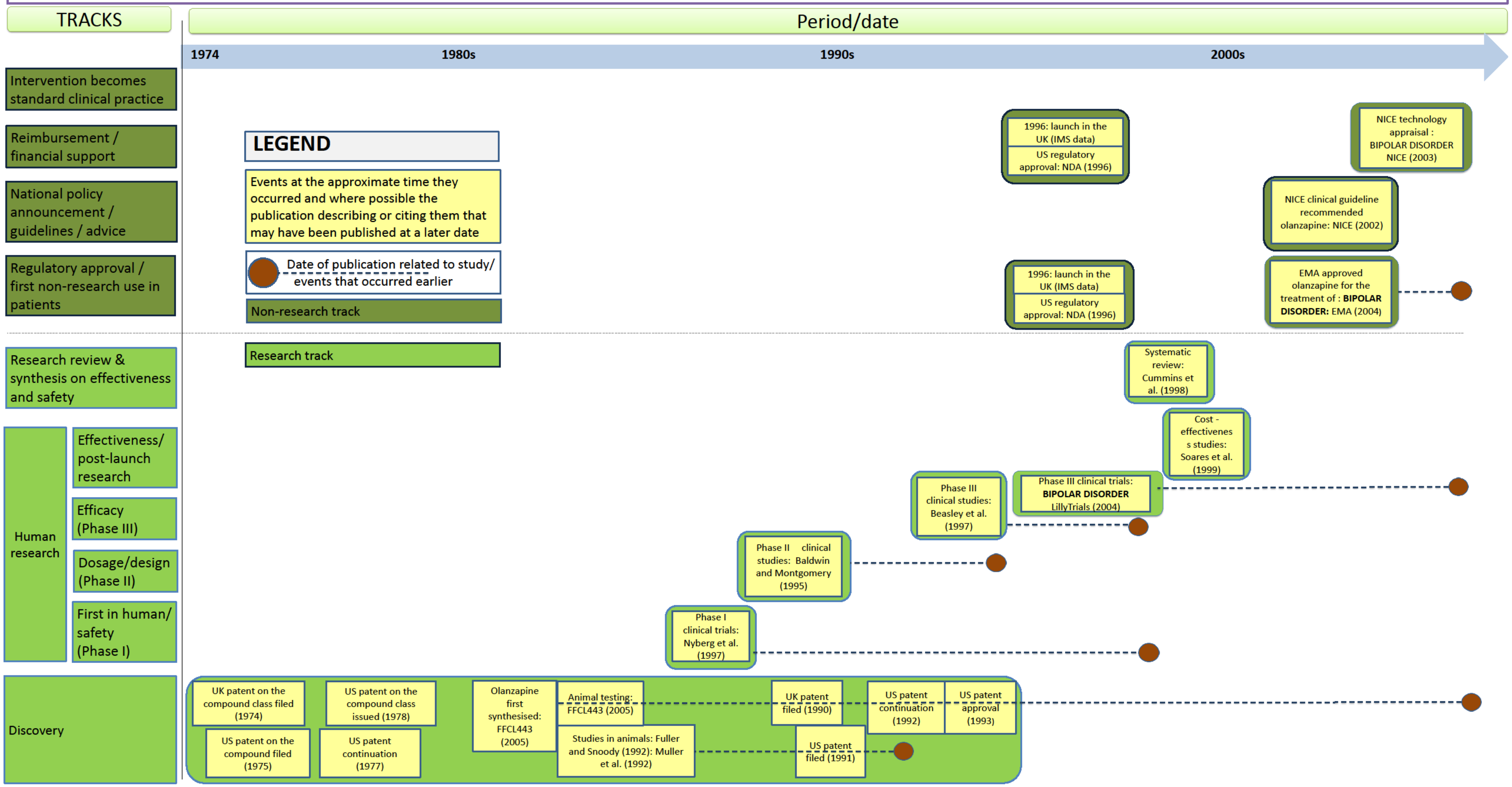

METHODOLOGICAL REFLECTIONS

This case study was conducted researching the online available information (both in published journals and in the grey literature) covering all the possible calibration points throughout the different tracks of the drug history, from early research to NICE clinical guidelines. This approach requires a deep knowledge of a drug research, development, launch and post-launch processes to identify the main events to be recorded; and involves both backward and forward tracing from the different calibration points. Valuable sources of information (which cannot be expected to be available in every drug case study) are the court cases, which reconstruct in detail the events related to the R&D of a drug.

There are advantages and disadvantages related to this approach. The main advantage is that this approach allows covering all the key stages of a drug history as it also considers the grey literature, which is a valuable (and sometimes the only) source of information for a drug commercial history (patents, regulatory application and approval, launch, national policy recommendation, uptake). When applied to more recent technologies, this approach potentially allows gathering more information, as it should be expected that drugs/interventions researched and developed in the Internet era have an extensive amount of records available online. One disadvantage of this approach is that it cannot be automated, as it requires seeking specific information manually. However, there are also economies of learning due to the recurrence of the main sources of information (e.g. the US Patent and Trademark Office or the FDA “Orange Book”).

Some observations can also be made about the information availability and validity. In general, clinical studies at the earlier stages of a drug development (phase I and II) are not usually published in peer-reviewed journals or considered in research synthesis publications (an exception is represented by “more popular” drugs or drugs whose patent has been disputed in a court case). Moreover, how far peer-review publications record the start date of clinical studies varies, and practice is evolving. Furthermore, the date of publication of the study may differ significantly from the date when the study took place, and online datasets of clinical trials (e.g. clinicaltrials.gov) are relatively recent and therefore contain little information relevant to older drugs.

It should also be noticed that many different and staggered patents can be associated with a single drug. In this case, interesting information can be obtained investigating why different patents were granted. For instance, in the olanzapine case, the new patent testifies how previous research was not able to detect which compound within the patented class was the best candidate to be developed.

It is also important to remember that NICE was set up in 1999. Therefore any measurement of lapsed time involving the publication date of a NICE guideline as calibration point should be considered as illustrative only if referred to interventions available before 1999.

REFERENCES

Baker RW, Ames D, Umbricht D, Chengappa KNR, Schooler NR (1995) Olanzapine's impact on depressive and obsessive-compulsive symptoms in schizophrenia. *Psychopharmacology Bulletin*. Pages 549

Baldwin DS, Montgomery SA. (1995) First clinical experience with olanzapine (LY 170053): results of an open-label safety and dose-ranging study in patients with schizophrenia. *Int Clin Psychopharmacol*. 1995 Nov;10(4):239-44.

Beasley CM Jr, Hamilton SH, Crawford AM, Dellva MA, Tollefson GD, Tran PV, Blin O, Beuzen JN. (1997). Olanzapine versus haloperidol: acute phase results of the international double-blind olanzapine trial. *Eur Neuropsychopharmacol*. May;7(2):125-37.

Chakrabarti, J.K. and Tupper, D.E. (1978). *U.S. Patent No. 4,115,574*. Washington, DC: U.S.

Chakrabarti, J.K., Hotten, T.M. and Tupper, D.E. (1993). *U.S. Patent No. 5,229,382*. Washington, DC: U.S.

Cummins, C., Stevens, A., & Kisely, S. (1998). *The use of olanzapine as a first and second choice treatment in schizophrenia*. NHS Executive South & West R&D Directorate.

Dittmann RW, Beasley CM, Geuppert M, Muhlenbacher D (1995) Clinical efficacy and safety of olanzapine: Results from a double-blind multinational clinical trial. *Pharmacopsychiatry*. Volume 28, Pages 172.

European Medicines Agency (EMA, 2004). CPMP/0646/96.

Food and Drug Administration (FDA, 1996). New drug application NDA 20-592.

Fuller RW, Snoddy HD. (1992) Neuroendocrine evidence for antagonism of serotonin and dopamine receptors by olanzapine (LY170053), an antipsychotic drug candidate. *Res Commun Chem Pathol Pharmacol*. Jul;77(1):87-93.

Kaitin, K. I., & Healy, E. M. (2000). The New Drug Approvals of 1996, 1997, and 1998: Drug Development Trends in the User Fee Era*. *Drug Information Journal*,*34*(1), 1-14.

LillyTrials (2004) Zyprexa clinical trials results. Online resource retrieved on 25 February 2013 from http://www.lillytrials.com/results/Zyprexa.pdf.

Moore NA, Tye NC, Axton MS, Risius FC (1992); The behavioral pharmacology of olanzapine, a novel "atypical" antipsychotic agent. *J Pharmacol Exp Ther* 262(2):545.

NICE Clinical Guideline (2002). Treating and managing schizophrenia (core interventions). Clinical Guideline 1, NICE: London.

NICE Technology Appraisal (2003). Full Guidance on olanzapine and valproate semisodium in the treatment of acute mania associated with bipolar disorder. Technology Appraisal TA66, NICE: London.

Nyberg, S., Farde, L., and Halldin, C. (1997). A PET study of 5-HT2 and D2 dopamine receptor occupancy induced by olanzapine in healthy subjects. *Neuropsychopharmacology*. 16 1–7.

Satterlee WG, Reams SG, Burns PR, Hamilton S, Tran PV, Tollefson GD (1995) A clinical update on olanzapine treatment in schizophrenia and in elderly Alzheimer's disease patients. *Psychopharmacology Bulletin*. Volume 31, Pages 534.

Soares BGO, Fenton M, Chue P. (1999) Sulpiride for schizophrenia. *Cochrane Database of Systematic Reviews*. Issue 1.

Tollefson G, Beasley C, Tran P, Sanger T (1995) Olanzapine: An Exciting Atypical Antipsychotic The Clinical Experience. *Conference Abstract* (8th European College of Neuropsychopharmacology Congress. Venice, Italy. 30th September - 4th October, 1995).

Tran PV , Beasley CM , Tollefson GD , Beuzen JN , Holman SL , Sanger TM and Satterlee WG. (1995) Olanzapine: A Promising "Atypical" Antipsychotic Agent. *Conference Abstract: Schizophrenia Research* (The Vth International Congress on Schizophrenia Research, Wormsprings, VA USA. 6th-12th April, 1995.), 1995, 1, 2(Spec).

UK Intellectual Property Office (UK IPO, 1974). Patent application GB51240/74.

UK Intellectual Property Office (UK IPO, 1990). Patent application GB9009229.

United States District Court Southern District of Indiana, Indianapolis Division (FFCL443, 2005). Eli Lilly and Company and Lilly Industries ltd. vs. Zenith Goldline Pharmaceuticals, Inc., Dr. Reddy's Laboratories, ltd., and Teva Pharmaceuticals USA, Inc. Findings of Fact and Conclusions of Law No. 443 ordered on 14 April 2005.

US Patent and Trademark Office (US PTO, 1978). US Patent Number 4,115,574.

US Patent and Trademark Office (US PTO, 1993). US Patent Number 5,229,382.

Worthen, S. T., Kasher, J. S., Saunders, J., Mckenzie, D. L., Hizer, R. E., Acklin, S., & Ramsey, J. T. (1997). The Global Registration of Zyprexa™(Olanzapine). *Drug information journal*, *31*(1), 49-55.

Time lags between conducting medical research and its translation. Case Study 3: screening programme for Abdominal Aortic Aneurysms (AAA)

NARRATIVE ACCOUNT OF BACKGROUND/ DEFINITIONS AND KEY ASPECTS OF THE INTERVENTION'S DEVELOPMENT

Background/definition

As reported in the main paper on this topic (Ashton et al., 2002), ruptured Abdominal Aortic Aneurysms (AAA) caused about 6,800 deaths in England and Wales in 2000. Most were men. In men older than 65 AAA are responsible for 2.1% of deaths. An aneurysm is a localised widening, or dilation, of an artery. The blood vessel can burst, or rupture, because the vessel wall is weakened. The abdominal aorta is the largest artery in the abdominal cavity, and, as part of the aorta, it is a direct continuation of the descending aorta (of the thorax). Of the deaths from aneurysms in the abdominal aorta about half took place before the patient reached hospital, with the mortality rate for emergency surgery being between 30%-70%.

Ultrasound can reliably visualise the aorta, thus providing the possibility of detection through population screening at a size when rupture is unlikely to occur. The hope was that intervention at this stage could reduce the frequency of rupture, and so reduce mortality and the requirement for emergency hospital treatment.

Early applications of ultrasound

The account below demonstrates that there is no absolutely clear-cut starting point for a study of a screening programme for AAA. First, we briefly describe the development of ultrasonics in medicine (and that builds on the development of ultrasonics in other fields). Then we relate its early use in diagnosing AAA in the USA, and in a few cases in the UK. We also describe how at the same time several other methods were being developed for diagnosing AAAs and the potential of these for use in screening programmes was considered by several authors. Several key accounts in the USA and the UK identify a small study by Cabellon and colleagues in 1983 as being the start of population screening for AAA by ultrasonography (Cabellon et al., 1983).

The account of the use of ultrasonics in medicine started with its application in therapy rather than diagnosis (Woo, 2002). The first experimental uses in diagnosis seem to have been in the 1940s but at the *First Congress of Ultrasound Medicine* held in 1948 in Germany just two papers discussed it as a diagnostic tool, the rest were on its therapeutic use. Systematic investigations into using ultrasound as a diagnostic tool in the US took off in the late 1940s (Woo, 2002), with studies by Ludwig at the Naval Medical Research Institute in Bethesda (Ludwig and Struthers, 1949). In the late 40s and into 1950s Wild conducted a stream of research that became supported by the National Cancer Institute and, according to Woo (2000), pioneered ultrasound imaging as described in a paper in *Science* (Wild and Reid, 1952). In the UK important work on the use of ultrasound in diagnosis was conducted by Donald in the Department of Midwifery, University of Glasgow, and published in the *Lancet* (Donald et al., 1958). The acknowledgements in this paper credit the considerable contribution made by a local company that seconded an employee full-time to work with the university team and speeded progress.

Donald did report one example where he had used ultrasound to diagnose an AAA (Donald and Brown, 1961). This example does not feature in some of the main accounts of the history of using ultrasound for AAA diagnosis, but was reported by Bernstein et al. (1978).

Rather more frequently the study published in the USA in Jan 1966 by Segal et al. (1966) is described as the first case report of ultrasound for detection and size measurement of a patient with an AAA. In Oct 1966 Goldberg et al. published the findings from a small series of cases of ultrasonic measurement, and referred to Segal et al. as 'The only previous report of ultrasonic diagnosis'. Following that in the late 60s, and 70s and into the 80s there were a string of papers in the USA describing the use of ultrasound in the diagnosis of AAA when other factors indicated the likely presence of an AAA, and its use for measurement of AAAs to assist decisions about surgery. In an article published in 1977 Sutton and Garner claimed the method did not seem to be widely used in the UK, but they described how since Feb 1971 they 'assessed by ultrasound all patients referred to the X-ray department, St Mary's Hospital, with a diagnosis of aortic aneurysm.' (1977, p.741). In Glasgow McGregor and colleagues (1975) reported using ultrasonography in the diagnosis of AAA: '59 patients with prominent abdominal pulsation were examined and aneurysm was demonstrated in 22.' (p.133).

Meanwhile various other approaches, beyond physical examination, were being explored to assist diagnosis and treatment of AAA. For six and a half years between January 1964 and May 1970, Schilling and colleagues studied 2663 subjects; 1517 of the total were examined by lateral abdominal roentgenography annually on 2 – 7 occasions. They concluded: 'inasmuch as many aneurysms are asymptomatic, routine radiologic surveys are indicated at age 55 as operative risk is less enhanced by degenerative disease.' (Schilling et al., 1974). A range of papers describe various techniques to diagnose AAAs, including descriptions of radiography, angiography and Computed Tomography –CT. In a chapter published in 1982, and based on a conference on AAAs, one of the major US teams stated: 'Improving ultrasonic and CT scanning technology should permit the risk-free and accurate diagnosis of abdominal aneurysms on a routine basis in an outpatient setting.' (Bernstein et al., 1982, p. 230). A team at the Walter Reed Army Medical Center in Washington led by Dr Norman Rich had been attempting to develop ways to better evaluate AAAs, but had abandoned a prospective study set up in the later 1970s because the angiography was sometimes misleading (Rich et al., 1982).

A member of that team, Dr Silverio Cabellon, moved to the William Beaumont Army Medical Center in Texas and decided CT scanning would be too expensive, but that he could use the new ultrasound equipment available to him in a prospective way to routinely diagnose the presence or absence of AAAs in all patients with peripheral atherosclerotic vascular disease who met certain criteria. He reported on all 73 patients examined in the first 3 months (Cabellon et al, 1983). This study has been identified in the US and the UK as the starting point for AAA screening by ultrasound. (see Scott et al. in MASS application 1996, Wolf et al,1995; Lederle, 2003; Lederle, 2008)

Key issues in the timeline of the development of AAA screening and analysis of possible time lags

The timeline from 1984 onwards is dominated by the work of one team in the UK (the Chichester team led by Alan Scott and their collaboration with the Health Economics Research Group (HERG), Brunel University, and the MRC Biostatistics Unit at the University of Cambridge.) The two RCTs

conducted by this team constitute over 75% of the weight of evidence in the international reviews on this topic. Key issues from this time line include the rapid start of the first RCT (the Chichester study) in 1989 even before the pilot Community cohort study being conducted by the team had been completed: the pilot had crucially demonstrated the feasibility of the methods. Likewise, an application for the major MASS trial was submitted before the Chichester study had been completed, and the proposal for the MASS study made clear that the trial was intended to inform the policy on screening. There was a time lag here and a revised proposal had to be submitted to address issues including the organisation of the proposed study to ensure it was large enough to be a powerful study, and the desire to wait until the results were known from a trial about the benefits of surgery on small aneurysms. (This illustrates a major factor with screening which is the desirability of fully understanding the appropriate way to treat those identified as having the condition for which the screening is being conducted. This is especially important in a field where the treatment option itself carries a risk).

There was again an attempt to reduce any time lags because the UK National Screening Committee (NSC), which had previously made clear in documents that it was waiting for the results from the MASS trial (UKNSC, 2001), requested a presentation from the MASS team before the publication of the findings. There was considerable discussion about how best to implement any screening programme, but the NSC's decision in principle to recommend going ahead with a programme was made in 2005 (UKNSC, 2005) even before a Cochrane systematic review was published in 2007 (Cosford et al., 2007). This decision is being considered as the equivalent of regulatory approval. There was further analysis, planning and discussions about aspects of implementation before the Ministerial decision to go ahead with the policy was announced in early 2008 (Brown, 2008). The subsequent analysis necessary for the Impact Assessment (DH, 2008) that accompanies government spending decisions drew on the Cochrane review as well as the MASS study. The impact assessment estimated the health gain that would arise from full implementation and suggested it could prevent 2,800 deaths a year. It also estimated the net benefit of the programme, based on the quality adjusted life years to patients gained, at £3,884.1m over 20 years. The programme was implemented in England in a phased way between 2009 – 2013. The continuing findings from the follow-up studies informed aspects of the implementation of the screening programme. Implementation of the NHS AAA screening programme in England was successfully completed in Spring 2013. According to the programme's web site: 'The NHS AAA Screening Programme covers the whole of England. The programme is delivered by 41 local screening services which together offer screening to around 300,000 men every year during the year they turn 65….AAA screening programmes will be fully implemented across Northern Ireland, Scotland and Wales by the end of 2013.' (NHS Abdominal Aortic Aneurysm Screening Programme – Frequently Asked Questions)

There are also indications that the insistence by the MRC that the study be revised to make it more powerful might eventually have helped the translation process because the quality of the study was referred to in various documents as being one of the reasons why the research was translated into policy. Furthermore, an important contextual point that helps explain the international significance of the UK study was set out in the proposal to the MRC: 'screening studies with a control population are not possible in countries with a predominantly private health care system.'

(In the USA where there were no RCTs, there was an even more rapid introduction of policy with the US Preventive Services Task Force systematic review in 2005 drawing heavily on the MASS study

(USPSTF, 2005; Fleming et al., 2005). Following that The Screening for Abdominal Aortic Aneurysms Very Efficiently (SAAAVE) Act was passed in 2006 and allowed Medicare to offer screening from January 2007 (Lederle, 2008). There is not, however, universal provision of AAA screening for those reaching 65).

Finally, it is possible to argue that the researchers, the MRC and the NSC combined in this case to play the role that a company plays in the development and uptake of a drug – proactively driving the research and its translation through to ultimate uptake and considerable health gain.

TIMELINE

Period/ Date	Study(ies)/Events	Track	Calibration point	Comment
**July 1983- Nov 1983**	First reported (uncontrolled) study of ultrasonic screening for AAA involving consecutive patients: Cabellon et al.	Human research	**Start of patient recruitment**: July 1983;<br>**End of patient recruitment**: Sept 1983;<br>**Publication:** Nov 1983: (Cabellon et al, 1983)	Generally described as the first ultrasonographic screening paper: Scott et al application to MRC (referenced here as MRC, Jan 1996); Wolf et al, 1995; review by Lederle, 2003 and 2008. Study was small involving just 73 patients, and selected population, but it was a sequence of all patients diagnosed at the Medical Centre as having atherosclerosis which was viewed as a major risk factor for AAA.
**1983-6**	Uncontrolled study of two groups of 100 hypertensive patients	Human research	**Presentation of findings:** Nov 1983<br>**Publication:**<br>***first****:* April 1984 – Abstract of conference presentation (Twomey et al)<br>***main****:* Twomey et al, 1986	Not known when patient recruitment started, and while findings presented same time as Cabellon publication the abstract not published until 1984, and main article not until 1986.
**1984-91**	Community cohort (uncontrolled) study in UK: informed the team's subsequent pilot RCT of ultrasonic screening for AAA by Scott et al	Human research	**Start of patient recruitment**: 1984;<br>**Publications:**<br>***first:*** Scott et al, 1988: brief account in BMJ;<br>***main:*** Scott et al, 1991 (163 cites);	In 1995 paper below (Scott et al) this 1984-91 community study (ie uncontrolled) is described as pilot for the main study (the Chichester study) which, in turn, is described as the first known RCT in this field. But that 'main study' started in 1988 well before results of 1984 study were published in 91. Results of 6 year pilot study perhaps less important than demonstration that able to undertake screening (as shown in 1988 brief account in BMJ). Contemporaneously there were six other non RCTs that reported between 1986 and 1992 and that were listed in the 1996 application to the MRC from Scott et al: in addition to Twomey et al, 2 studies of selected populations: Allardice et al, 1988; and Bengtsson et al, 1989; and 3 studies of unselected populations: Collin et al, 1988; (201 cites); O'kelly and Heather, 1989; Smith et al, 1992. In some the data collection started early, eg Allardice et al, 1988 started in Feb 1984. Collin became part of the MASS team.
**1989-1995 (and on to 2002)**	The Chichester Study. Pilot RCT: Involved 15,775 people.	Human research	**Start of data collection:** 1989;<br>**Publications:**<br>***main:*** Scott et al, 1995 (216 cites);<br>***important follow-up:*** Vardulaki et al,	In the proposal for Multicentre Aneurysm Screening Study (MASS) (large RCT), the Chichester study is described as 'a five year pilot study of 15,000 men and women aged 65-80yr in a randomised controlled trial.'

			2002.<br>***final follow-up:*** Ashton et al, 2007.	Despite being described as a pilot RCT, the findings from Chichester study included (along with those from 3 others) in the Cochrane review, and other reviews.<br>Contributed 10.0% of the weight in the US Preventive Services Task Force review (Fleming et al, 2005)
**1993-2002 (and on to 2012)**	Multi-centre Aneurysm Screening Study (MASS study): RCT: involved 67,800 men. (Continuing follow-up studies reporting data at 7, 10 and 13 years.)	Human research (including effectiveness and post-launch research)	**Original proposal submitted:** Nov 1993 to MRC;<br>**Revised proposal submitted:** Oct 1995 to MRC;<br>**Proposal funded:** April 1996;<br>**Start of patient recruitment:** Jan 1997;<br>**Last patient recruited:** May, 1999;<br>**Publications:**<br>***first***: Ashton et al, Lancet, 2002 (472 cites)<br>Buxton/MASS, BMJ; 2002 (143 cites): simultaneous publication of clinical and cost effectiveness studies.<br>***important further analyses:***<br>Kim et al, 2007; (7 year data)<br>Thompson et al, 2009; (10 year data)<br>***final follow-up:*** Thompson et al, 2012; (13 year data)	Proposal turned down (as described above) partly because results of surgery trial were not yet available;<br>Jan 1996 meeting of MRC's Health Services and Public Health Research Board asked for the proposal to be revised and re-submitted.<br><br>UK National Screening Committee (NSC) asked for an early presentation of findings, prior to publication.<br><br>Contributed 65.9% of the weight in the US Preventive Services Task Force review (Fleming et al, 2005)
**1994 - 2005**	RCT: Denmark: involved 12,658 people.	Human research	**Start of patient recruitment:** April 1994<br>**Last patient recruited:** 1998/9<br>**Publications:**<br>***early:*** Lindholt et al, 1996;<br>***preliminary results***: Lindholt et al, Ugeskrift for Laeger, 1997 [in Danish]<br>***important papers:*** Lindholt et al, 2002;<br>Lindholt et al, BMJ, 2005	Danish studies complex with RCT starting recruiting earlier than MASS (April 1994), but smaller studies; important reporting in 2002 and 2005, but also some earlier papers.<br>Contributed 7.3% of the weight in the US Preventive Services Task Force review (Fleming et al, 2005)
**I995 – 2004**	RCT: Western Australia: involved 38,704 people.	Human research	**Start of patient recuitment:** April 1996;	Study started recruiting earlier than MASS: April 1996, but main findings not reported until after: Oct 2004.

			**Publications:**<br>***early comment:*** Spencer et al, 2000;<br>***initial results:*** Lawrence-Brown et al, 2001;<br>***first full results:*** Norman et al, 2003<br>***main:*** Norman et al, BMJ; 2004.	Contributed 16.8% of the weight in the US Preventive Services Task Force review (Fleming et al, 2005)<br>Early comment by Spencer et al (2000) that selective screening for AAA using easily recognisable risk factors is feasible, but not worthwhile as approx. 25% of the clinically significant cases would be missed.
**2003**	First major systematic review identified: Lederle, 2003.	Research review	**Publication:** Lederle, 2003.	The 4 studies described above were the only ones included: Chichester; MASS; Western Australia; Denmark. MASS the largest.<br>Also Cabellon et al (1983) is the first ultrasonography study mentioned.
**2005**	First internationally recognised review conducted for a government agency: US Preventive Services Task Force (Fleming et al, 2005)	Research review	**Publication:** Fleming et al, 2005	The 4 studies described above were the only ones included: Chichester; MASS; Western Australia; Denmark. MASS the largest. In 2005 in addition to the article by Fleming et al., the report published from this review (USPSTF, 2005) contained two elements. The Evidence Synthesis of the Cost-Effectiveness Analyses of Population-Based Screening for Abdominal Aortic Aneurysm highlights the quality of the cost effectiveness analysis in MASS. It states: 'we believe that the detailed micro-costing approach used in the MASS CEA, as well as its use of probabilistic sensitivity analysis, mitigated its being set outside the United States (it was conducted in the United Kingdom) and justified a "good" quality rating.' (USPSTF, 2005)
**2005**	UK National Screening Committee recommendation to ministers for introduction of a population screening programme in principle.	Regulatory approval	**Regulatory approval:** NSC, 2005: 'Screening of men aged 65, with the offer of a single test being made at that age, can be recommended in principle as a programme that meets the criteria and standards of the National Screening Committee. The configuration of services is a critical issue to be considered further before any implementation of new screening programmes.' (UKNSC, Nov, 2005)	The decision in principle is here viewed as the equivalent to regulatory approval, but the decision to introduce a national population screening policy has to be taken in the UK by ministers, and further work was required.<br>There is evidence from the files of considerable liaison (dissemination) between the research team and the NSC leading up to and after the decision by NSC. Nov 2004: Presentations to NSC Workshop; Feb 2005: presentation to NSC;<br>Oct 2006: NSC Workshop on National Plan for AAA screening

**2007**	First major UK review: Cochrane review (Cosford et al, 2007)	Research review	**Publication:** (Cosford et al, 2007)	The 4 studies described above were the only ones included: Chichester; MASS; Western Australia; Denmark. MASS the largest.<br>Introduction noted that the health department considered the evidence was sufficiently strong to introduce screening, through a managed development of a co-ordinated service, but it continued: 'There are, however, no published systematic reviews of the evidence for screening from randomised controlled trials.'
**Jan 2008**	Announcement by Prime Minister Gordon Brown of Government policy to introduce a national programme of AAA screening	National policy announcement/ guidelines/advice	**National policy announced:**<br>Jan 2008 PM's speech to mark 60th anniversary of founding of NHS (Brown, 2008).	Policy announced as part of PM's speech to mark 60th anniversary of founding of NHS.
**July 2008**	Financial support level agreed by Department of Health in England	Reimbursement/ financial support	**Financial support:** July 2008: Impact assessment to support policy signed off by Health Minister and published by DH: DH Impact Assessment of a National Screening Policy for Abdominal Aortic Aneurysms (DH, 2008).	The Impact Assessment draws heavily on evidence from MASS and Cochrane review to support implementation of the screening programme. The impact assessment estimated the health gain that would arise from full implementation and suggested it could prevent 2,800 deaths a year. It also estimated the net benefit of the programme, based on the quality adjusted life years to patients gained, at £3,884.1m over 20 years.
**2009**	**Clinical guideline:** The Society for Vascular Surgery (USA). Recommended one-time ultrasound screening for AAA for all men over 65.	(National policy announcement/ guideline/advice: but USA not UK)	**USA Clinical guideline:** first known international Clinical Guideline recommending AAA screening Chaikof et al (2009)	Guideline identified 4 RCTs, ie the 4 described above. Recommendation: 'One-time ultrasound screening for AAA is recommended for all men at or older than 65 years. Screening men as early as 55 years is appropriate for those with a family history of AAA.' The Level of recommendation was described as 'Strong', the Quality of evidence described as 'High'.<br><br>As early as Jan 2004 there had been advice in form of Consensus Statement from 3 American vascular societies recommending AAA screening (Kent, 2004) with broader criteria than those used by the subsequent US Preventive Services Task Force. The Statement referred to various papers from the above studies, especially MASS.

**2009-13**	Phased implementation of the NHS Abdominal Aortic Aneurysm Screening Programme (NAAASP) began in 2009 with coverage throughout England being completed in Spring 2013	Intervention becomes standard practice	**Intervention becomes standard practice:** phased implementation began in 2009 with coverage throughout England being completed in Spring 2013	In some parts of the UK there had already been a local introduction of screening, but standard practice is taken to be the introduction of the national programme, even though that took until Spring 2013 to complete. Introduced in 2012 in Northern Ireland, Scotland and Wales.<br>According to the programme's web site: 'The NHS AAA Screening Programme covers the whole of England. The programme is delivered by 41 local screening services which together offer screening to around 300,000 men every year during the year they turn 65….AAA screening programmes will be fully implemented across Northern Ireland, Scotland and Wales by the end of 2013.' (NHS Abdominal Aortic Aneurysm Screening Programme – Frequently Asked Questions)

Matrix: AAA screening: 26 years from first trial to standard practice in the UK

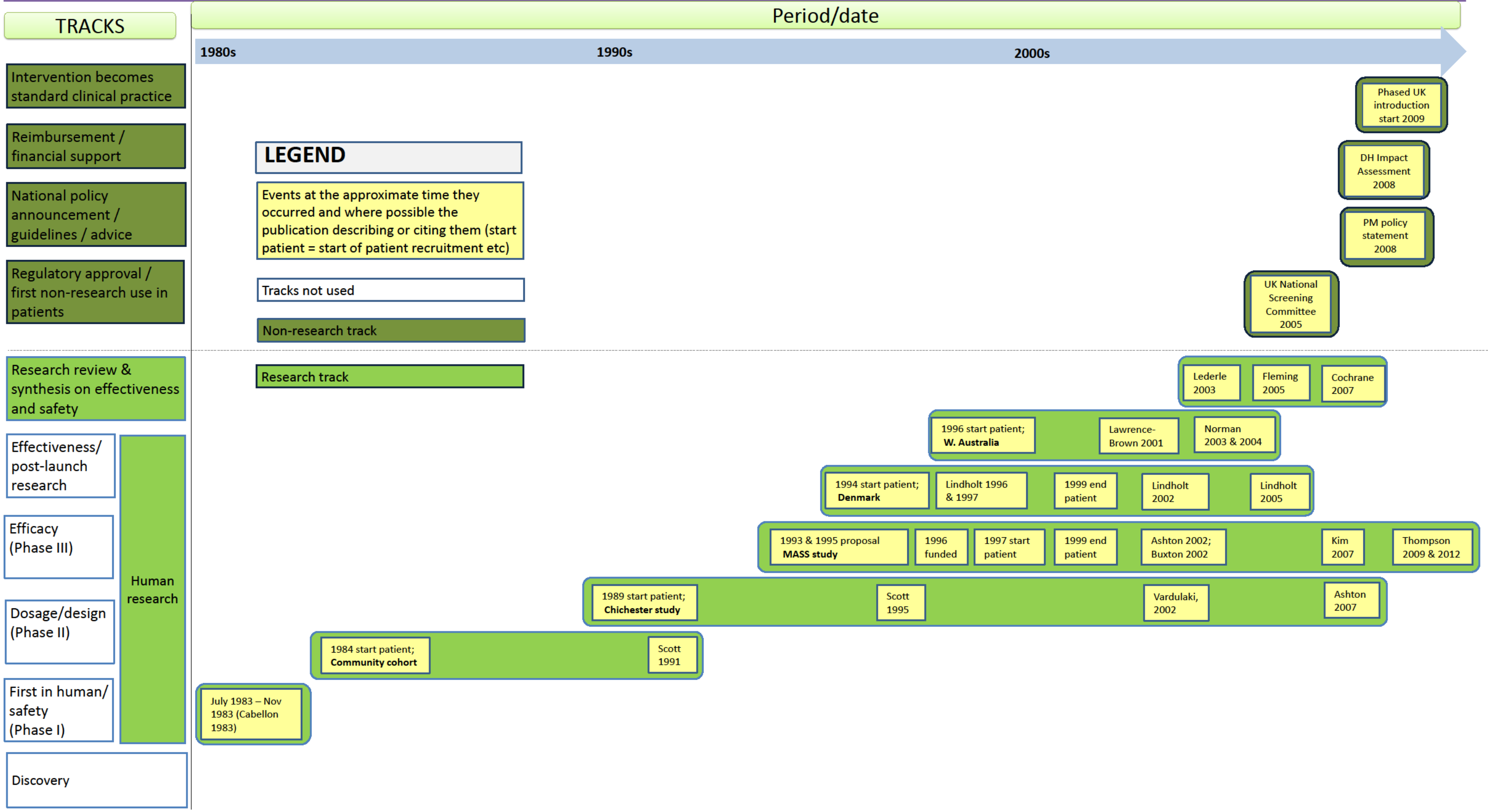

METHODOLOGICAL REFLECTIONS

In this case it was possible to make considerable progress in identifying the detailed steps of the timeline, and assess the time elapsed as being 26 years from the first screening study to the intervention becoming standard practice. A full range of case study techniques was successfully deployed to make this progress: documentary and archival analysis (which was greatly aided by a leading member of the MASS team, Prof Martin Buxton, being a member of the time lags project team); a Google search; a review of relevant publications; interview with a key player, Dr Silverio Cabellon, who conducted the first AAA ultrasound screening study.

For AAA it was possible to identify start points/start of patient recruitment for some key projects and these were very important for understanding the nature of the time line, eg the case study showed how the first RCT started before the pilot study had published its findings, so it would have been misleading had the case study relied solely on publications. Also for the MASS study, the information about the date the proposal was originally submitted and then re-submitted was very useful and would have been hidden by a reliance solely on the publication dates of the first RCT, which was described as a pilot for the bigger RCT.

AAA was one of the cases where it was also possible to gather information about some important markers listed in Trochim but excluded from our final collective list. In some instances these proved to be very useful for understanding time lag issues, for example in MASS the details about the end of patient recruitment enable us to see that the study involved a relatively short period of patient recruitment, but a longer follow-up period.

We might need to consider if the scope or length of some of the studies was sufficient, for example while the pharmaceutical case studies described earlier identified important time lags by going back into the Discovery track, this part was missing from the AAA study which only focussed on a shorter span and so did not analyse possible delays that were touched on in the background to the case study.

The matrix allows the presentation of the data in a way that clearly demonstrates what may be a feature of the time lines for screening research, ie a relatively long research period, which is necessary to conduct thorough studies of the potential benefits of introducing screening policies, followed by a reasonably rapid translation into policy that is then implemented. In this case, however, the usual phases of human research that are relevant in a pharmaceutical intervention do not translate neatly into the types of studies conducted for a screening intervention.

There were a few aspects, such as the first small uncontrolled study in the UK by Twomey et al (1984 and 1986), where some details are missing from this account and it might have been useful to undertake further interviews. However, this case study concentrated on a specific intervention and was able to work along the time dimension reasonably comprehensively by focussing on the details of the events and studies that were key to understanding the time elapsed, and the causes of the small delays that did occur.

REFERENCES

Allardice JT, Allwright GJ, Wafula JMC, Wyatt AP. (1988) High prevalence of abdominal aortic aneurysm in men with peripheral vascular disease: screening by ultrasonography. *Br J Surg*;**75:**240-2.

Ashton HA, Buxton M, Day NE, Kim LG, Marteau TM, Scott RAP, Thompson SG, Walker NM (on behalf of the Multicentre Aneurysm Screening Study Group) (2002) The Multicentre Aneurysm Screening Study (MASS) into the effect of abdominal aortic aneurysm screening on mortality in men: a randomised controlled trial, *Lancet*;**360**:1531-9.

Ashton HA, Gao L, Kim LG, Druce PS, Thompson SG, Scott RA. (2007) Fifteen-year follow-up of a randomized clinical trial of ultrasonographic screening for abdominal aortic aneurysms. *Br J Surg*;**94:**696-701.

Bengtsson H, Norrgard Ő, Ängquist KA, Ekberg O, Őberg L, Bergqvist D. (1989) Ultrasound screening of the abdominal aorta among siblings of patients with abdominal aortic aneurysms. *Br J* Surg;**76:**589-91.

Bernstein EF, Dilley RB, Goldberger LE, Gosink BB, Leopold GR. (1976) Growth rates of small abdominal aortic aneurysms. *Surgery*;**80**(6):765–73.

Bernstein EF, Harris RD, Leopold GR. (1982) Diagnosis of aneurysms by ultrasound and computed tomography. In: Bergan JJ, Yao JST (eds). *Aneurysms: Diagnosis and Treatment.* New York: Grune & Stratton; 217-31.

Brown G. (2008) Speech on the National Health Service (7 January 2008). http://webarchive.nationalarchives.gov.uk/20090114000528/number10.gov.uk/page14171 [Last accessed 14 July 2014]

Buxton M, Ashton H, Campbell H, Day NE, Kim LG, Marteau TM, Scott RAP and Thompson SG (2002) (or MASS, 2002) Multicentre aneurysm screening study (MASS): cost effectiveness analysis of screening for abdominal aortic aneurysms based on four year results from randomised controlled trial. *BMJ*;**325**:1135-8.

Cabellon S, Moncrief CL, Pierre DR, Cavenaugh DG. (1983) Incidence of abdominal aortic aneurysms I patients with artheromatous arterial disease. *Am J Surg*;**146:**575-6.

Chaikof EL, Brewster DC, Dalman RL, Makaroun MS, Illig KA, Sicard GA, Timaran CH, Upchurch GR Jr, Veith FJ; Society for Vascular Surgery. (2009) The care of patients with an abdominal aortic aneurysm: the Society for Vascular Surgery practice guidelines. *J Vasc Surg*;**50**(4 Suppl):S2-49.

Cosford PA, Leng GC, Thomas J. (2007) Screening for abdominal aortic aneurysm. *Cochrane Database Syst Rev* 2007;(2)CD002945.

Department of Health. (2008) *Impact Assessment of a national Screening Programme for Abdominal Aortic Aneurysms. Department of Health.* http://webarchive.nationalarchives.gov.uk/20080726153931/http://dh.gov.uk/en/Publicationsandstatistics/Publications/PublicationsLegislation/DH_086050 [Lat accessed 14 July 2014]

Donald, I., MacVicar, J. and Brown, T.G. (1958) Investigation of abdominal masses by pulsed ultrasound. *Lancet* **271:**1188-95.

Donald I, Brown TG. (1961) Demonstration of tissue interfaces within the body by ultrasonic echo sounding. *Brit J Radiology;* **xxxiv:**539-46.

Fleming et al. (2005) Screening for abdominal aortic aneurysm: A best-evidence systematic review for the US Preventive Services Task Force. *Ann Intern Med*;**142:**203-11.

Goldberg BB, Ostram BJ, Isard HJ. (1966) Ultrasonic aortography. *JAMA*;**198**(4):119-24.

Kent KC, Zwolak RM, Jaff MR, Hollenbeck ST, Thompson RW, Schermerhorn ML, Sicard GA, Riles TS, Cronenwett JL; Society for Vascular Surgery; American Association of Vascular Surgery; Society for Vascular Medicine and Biology. (2004) Screening for abdominal aortic aneurysm: A consensus statement. *J Vasc Surg*;**39**:267–9.

Kim L, Thompson S, Briggs A, Buxton M, Campbell H (2007) How cost-effective is screening for abdominal aortic aneurysms? *J Med Screening*; **14**:46-52.

Lawrence-Brown MM, Norman PE, Jamrozik K, Semmens JB, Donnelly NJ, Spencer C, et al. (2001) Initial results of ultrasound screening for aneurysm of the abdominal aorta in Western Australia: relevance for endoluminal treatment of aneurysm disease. *Cardiovasc Surg*;**9(**3):234-40.

Lederle FA. (2003) Ultrasonic screening for abdominal aortic aneurysms. *Ann Intern Med;* **139:**516-22.

Lederle FA. (2008) Screening for AAA in the USA. *Scan J Surg;* **97:**139-41.

Lindholt JS, Henneberg EW, Fasting H, Juul S. (1996) Hospital based screening of 65-73 year old men for abdominal aortic aneurysms in the county of Viborg, Denmark. *J Med Screening*;**3(**1):43-6.

Lindholt JS, Fasting H, Henneberg EW, Juul S. (1997) [Preliminary results of screening for abdominal aortic aneurysm in the county of Viborg]. [Danish]. *Ugeskrift for Laeger*;**159**(13):1920-3.

Lindholt JS, Juul S, Fasting H, Henneberg EW. (2002) Hospital costs and benefits of screening for abdominal aortic aneurysms. Results from a randomised population screening trial. *Eur J Vasc Endovasc Surg*;**23**(1):55-60.

Lindholt JS, Juul S, Fasting H, Henneberg EW. (2005) Screening for abdominal aortic aneurysms: single centre randomised controlled trial. *BMJ*;**330**(7494):750-3.

Ludwig, G.D. and Struthers, F.W. (1949) Considerations underlying the use of Ultrasound to detect Gallstones and Foreign Bodies in Tissue. Naval Medical Research Institute Reports, Project #004 001, Report No. 4, June 1949. [Available in Woo, 2002]

MASS, 2002: *See Buxton et al. 2002 above.*[Same publication but referenced in different ways in different places].

McGregor JC, Pollock JG, Anton HC. (1975) The value of ultrasonography in the diagnosis of abdominal aortic aneurysm. *Scott Med J*;**20:**133e7.

Medical Research Council. (Jan 1996) Multi-centre Aneurysm Screening Study (MASS) application.

NHS Abdominal Aortic Aneurysm Screening Programme. Frequently Asked Questions. http://aaa.screening.nhs.uk/questions [Last accessed 14 July 2014]

Norman PE, Jamrozik K, Lawrence-Brown M, Dickinson J. (2003) Western Australia randomised controlled trial of screening for abdominal aortic aneurysms [Abstract]. *Br J Surg*;**90:**492.

Norman PE, Jamrozik K, Lawrence-Brown MM, Le MT, Spencer CA, Tuohy RJ, et al. (2004) Population based randomised controlled trial on impact of screening on mortality from abdominal aortic aneurysm. *BMJ*;**329**(7477):1259-64.

O'Kelly TJ, Heather BP. (1989) General practice population screening for abdominal aortic aneurysm: a pilot study. *Br J Surg*;**76:**479-80.

Rich NM, Clagett GP, Salander JM, Cabellon S, Eddelman WL. (1982) Role of arteriography in the evaluation of aortic aneurysms. In: Bergan JJ, Yao JST (eds). *Aneurysms: Diagnosis and Treatment*. New York: Grune & Stratton; 233-55.

Schilling FJ, Christakis G, Hempel HH, Orbach A. (1974) The natural history of abdominal aortic and iliac atherosclerosis as detected by lateral abdominal roentgenograms in 2663 males. *J Chronic Dis*;**27:**37–45.

Scott RAP, Ashton H, Sutton GLJ. (1988) Ultrasound screening of a general practice population for aortic aneurysms. *Br J Surg;* **73:**318.

Scott RAP, Ashton HA, Kay DN. (1991) Abdominal aortic aneurysm in 4327 screened patients: prevalence, development and management over 6 years. *Br J Surg*;**78:**1122-5.

Scott RAP, Wilson NM, Ashton HA, Kay DN. (1995) Influence of screening on the incidence of ruptured abdominal aortic aneurysm: 5 year results of a randomised controlled study. *Br J Surg*;**82:**1066-70.

Segal BL, Likoff W, Asperger Z, Kingley B. (1966) Ultrasound diagnosis of an abdominal aortic aneurysm. *Amer J Cardiol;* **17:**101-3.

Smith FC, Grimshaw GM, Paterson IS, Shearman CP, Hamer JD. (1992) Ultrasound screening for abdominal aortic aneurysm in an urban community. *Br J Surg*;**79:**A358.

Spencer CA, Jamrozik K, Norman PE, Lawrence-Brown MM. (2000) The potential for a selective screening strategy for abdominal aortic aneurysm. *J Med Screening*;**7**(4):209-11.

Sutton D, Garner RG. (1977) Ultrasound in the diagnosis and measurement o aneurysms of the abdominal aorta. *Postgraduate Med J;* **53:**741-4.

Thompson SG, Ashton HA, Gao L, Buxton MJ and Scott RAP on behalf of the Multicentre Aneurysm Screening Study (MASS) Group. (2012) Final follow-up of the Multicentre Aneurysm Screening Study (MASS) randomized trial of abdominal aortic aneurysm screening. *Br J Surg*;**99:**1649-56.

Thompson SG, Ashton HA, Gao L, Scott RA; Multicentre Aneurysm Screening Study Group. (2009) Screening men for abdominal aortic aneurysm: 10 year mortality and cost effectiveness results from the randomised Multicentre Aneurysm Screening Study. *BMJ*;**338:** b2307.

Twomey A, Twomey EM, Wilkins RA, Lewis JD. (1984) Unrecognized aneurysmal disease in male hypertensive patients. *Br J Surg*;**71:**307-8.

Twomey A, Twomey E, Wilkins RA, Lewis JD. (1986) Unrecognised aneurysmal disease in male hypertensive patients. *Int Angiol*;**5:**269–73.

US Preventive Services Task Force (2005): *Screening for Abdominal Aortic Aneurysm: Recommendation Statement.* http://www.uspreventiveservicestaskforce.org/uspstf/uspsaneu.htm and *Cost-Effectiveness Analyses of Population-Based Screening for Abdominal Aortic Aneurysm. Evidence Synthesis.* http://www.uspreventiveservicestaskforce.org/uspstf05/aaascr/aaacost.htm

UK National Screening Committee. (2001) *Second Report of the UK National Screening Committee.* Department of Health. http://www.google.co.uk/url?url=http://www.aogm.org.mo/assets/Uploads/aogm/Guidelines/RCOG---UK/Second-report-of-UK-NSC.pdf&rct=j&frm=1&q=&esrc=s&sa=U&ei=jDLEU-iSH-HR7AbCrYGoDQ&ved=0CCwQFjAE&usg=AFQjCNFUQd0oXWzTQaQeYrvY8OAa3Fk1Mg [Last accessed 14 July 2014].

UK National Screening Committee. (2005) *Screening for Abdominal Aortic Aneurysm*. http://aaa.screening.nhs.uk/getdata.php%3Fid%3D742&rct=j&frm=1&q=&esrc=s&sa=U&ei=kfrDU4OLNYr17AaF5YGIDw&ved=0CBoQFjAB&usg=AFQjCNFvPf1Nz65ZzFhVleX5e3VNDQpj_Q [Last accessed 14 July 2014].

Vardoulaki, K.A., Walker, N.M., Day, N.E., Duffy, S.W., Ashton, H.A., Scott, R.A. (2000) Quantifying the risks of hypertension, age, sex and smoking in patients with abdominal aortic aneurysm. *Br J Surg*;**87:**195–200.

Wild JJ, Reid JM. (1952) Application of echo-ranging techniques to the determination of structure of biological tissues. *Science;***115**:226-30.

Wolf YG, Otis SM, Schwend RB, Bernstein EF. (1995) Screening for abdominal aortic aneurysms during lower extremity arterial evaluation in the vascular laboratory. *J Vasc Surg*;**22:**417-23.

Woo JSK. (2002) A Short History of the Development of Ultrasound in Obstetrics and Gynecology. Available at: http://www.ob-ultrasound.net/history1.html (Accessed 9 June 2013).

Time lags between conducting medical research and its translation. Case Study 4: Smoking reduction

NARRATIVE ACCOUNT OF BACKGROUND/ DEFINITIONS AND KEY ASPECTS OF THE INTERVENTION'S DEVELOPMENT

While this case study focuses on smoking as a cause of cardiovascular disease, much of the evidence and policy reviewed relates to the relationship between smoking and lung cancer. This is because it was this relationship with cancer which led to the majority of policy responses and interventions, and as such the evidence relating to cancer is a more appropriate point to start from when exploring the time lag. For comparison, however, the timeline does note key research, reviews and statements relating to cardiovascular disease.

Although writers had speculated about a link between tobacco and cancer since the 1850s, it was not until 1912 that Isaac Adler first proposed that smoking (as opposed to tobacco dust) might be a cause of lung cancer. The first links between tobacco and arteriosclerosis were also suggested in the late 19th century. The number of deaths from lung cancer increased dramatically in the first half of the 20th century, leading to the MRC organising a conference in 1947 to examine possible causes. The suggestion at this conference that tobacco, and in particular cigarette smoking, might be a factor led to the MRC funding a case control study to look at the link between smoking and lung cancer (Doll & Hill, 1950). This study, along with four US studies published the same year, all concluded that cigarette smoking was likely a causal factor in the development of lung cancer.

Building on these studies, and realising that a different kind of evidence was needed to make a conclusive case, Doll and Hill began a prospective cohort study of 40,000 British doctors, tracking their smoking habits and investigating whether it was possible to predict lung cancer risk. Preliminary results in 1954 suggested an association between smoking and lung cancer, as well as some effect on coronary thrombosis (Doll & Hill, 1954), while in the same year early findings from a large US study indicated a higher death rate among smokers from both lung cancer and coronary artery disease (Hammond & Horn, 1954). Although it had first been suggested back in 1934 that smoking might contribute to coronary thrombosis, and further evidence of a link with cardiovascular disease had emerged during the 1940s, this had not been conclusive.

Further findings from the British Doctors Study were published in 1956, and even at this early stage the authors were able to conclude a death rate from cancer that was 20 times higher in smokers compared with non-smokers. At the same time, evidence was also beginning to build on the nature of tobacco, with the discovery of carcinogens in tobacco smoke and evidence on the effects of tar in causing tumour growth in animals. In 1957, both the MRC and the US Surgeon General declared a causal link between cigarette smoking and cancer, and in the following years, influential reports from the US Surgeon General and the Royal College of Physicians summarised the evidence and recommended a variety of restrictions on sales and marketing.

Throughout the 1960s and 1970s, a range of advertising restrictions were introduced (with a UK ban on TV advertising of cigarettes enforced in 1965) and various anti-smoking campaigns implemented, focused both on lobbying for smoke-free environments (for which public support was growing) and

on encouraging people to stop smoking. During this time the evidence on smoking harms had also continued to build, both from the British Doctors Study's ongoing analysis and more widely, with an increasing emphasis on the potential effects of environmental tobacco smoke. The first epidemiological evidence linking passive smoking to lung cancer was published in 1981, and in 1986 a number of reports concluded a causal link, leading to proposals for further restrictions on smoking in public places (e.g. Froggatt, 1988). Similarly conclusive epidemiological evidence on the role of passive smoking in cardiovascular disease was published in 1991 (Glantz & Parmley, 1991). As part of the UK Government's 1994 action plan to reduce smoking, a new Scientific Committee on Smoking and Health was launched. The Committee published a review of the evidence on passive smoking in 1998, concluding that it is a cause of lung cancer, and in the years that followed a number of reports called for a ban on smoking in public places (including from the British Medical Association and the Chief Medical Officer). In 2004 the Scottish First Minister announced that Scotland would implement a total ban on smoking in workplaces and public places, while the Department of Health released a white paper proposing similar legislation for the majority of workplaces and public places in England and Wales. A similar announcement for Northern Ireland was made the following year. In 2006 Scotland became the first UK country to implement smokefree legislation, with Northern Ireland, England and Wales following in 2007.

At the same time as evidence was mounting on the effects of passive smoking, there was also an increase in the availability of smoking cessation services to individuals (initially through organisations such as ASH and QUIT, then on the NHS from 1998) and moves to further restrict tobacco advertising. In 1990 the European Parliament voted in favour of a total ban on advertising, which led to a European Commission proposal for a ban in 1991, and the eventual adoption of an EU Directive banning advertising and sponsorship in Member States in 1998. This Directive was overturned by the European Court of Justice three years later as its implications were deemed beyond the EU's powers, but a more restricted Directive replaced it in 2002. In the UK, the 1992 Smee report summarised the evidence on the effects of tobacco advertising on consumption and the 1994 action plan to reduce smoking included measures to restrict advertising. In 1996 Guernsey became the first government in the British Isles to agree a complete ban, before a similar bill was passed in the UK parliament in 2002. Most recently, the UK Government launched a public consultation on plain packaging of cigarettes (2012), with a survey suggesting that 62% of adults in England are in favour.

TIMELINE

Key – colours represent different categories of 'intervention'

- Taxation
- **Legislation – including**
  - advertising restriction
  - sales restriction
  - smokefree legislation
- Public Health campaigns

Period/date	Study/Event	Track	Calibration point	Comments
1761	John Hill, an English physician, reports a case of nasal snuff causing cancer of the nose – the next case is not documented until 2007…	Discovery		
1850s	Writers first link tobacco to cancer, particularly of the mouth and face	Discovery	**Publications:** (Jacobs, 1855; Shew, 1854)	
1893	Huchard (1893) suggests that nicotine causes arteriosclerosis, and cites the experiments of Claude Bernard in demonstrating that nicotine produces arterial hypertension by vasoconstriction	Discovery	**Publication:** (Huchard, 1893)	
1898	Hermann Rottmann, a medical student, in Würzburg proposed that tobacco dust—not smoke—might be causing the elevated incidence of lung tumours among German tobacco workers	Discovery		**Source:** (R. N. Proctor, 2012)
1904	Erb (1904) finds that 25 out of 45 patients with intermittent claudication were heavy smokers	Discovery	**Publication:** (Erb, 1904)	
1908	Buerger (1908) describes a rare form of vascular disease in young people and notes that it rarely occurs in non-smokers. These findings are confirmed by several others over the following 30 years, but the occasional diagnosis of the disease in non-smokers was considered to rule out	Discovery	**Publication:** (Buerger, 1908)	**Source:** (Doll, 1998**)**

	smoking as a cause.			
1912	Isaac Adler first links smoking with lung cancer (rather than tobacco dust), but states that this is only a possible cause and notes that there is not sufficient evidence (Adler, 1912)	Discovery	**Publication:** (Adler, 1912)	
1934	First suggestion that smoking might be responsible for an increase in coronary thrombosis	Discovery	**Publication:** (Howard, 1934)	
1939	Müller (1939) publishes first case control study, comparing retrospectively smoking behaviour of patients who had died of lung cancer with healthy controls. He concluded that "the extraordinary rise in tobacco use" was "the single most important cause of the rising incidence of lung cancer".	Discovery	**Publication:** (Müller, 1939)	Details in English in Proctor (2000) and Doll (1998).
1940	English et al. (1940) compare (i) the habits of 1000 patients with coronary thrombosis to 1000 matched controls, and (ii) the incidence of coronary disease in 1000 smokers compared with 1000 non-smokers. They conclude that smoking tobacco probably has "a more profound effect on younger individuals owing to the existence of relatively normal cardiovascular systems, influencing perhaps the earlier development of coronary disease".	Discovery	**Publication:** **(**English, et al., 1940)	
1944	Schairer & Schöningen (1944) support Müller's findings with a larger sample and comparison stomach cancer group.	Discovery	**Publication:** (Schairer & Schöniger, 1944)	Details in Doll (1998)
Late 1940s	Numerous reports in Germany of tobacco causing hypertension, angina and atherosclerosis	Discovery	**Publications**	Source: (Doll, 1998)
1947	MRC conference on possible causes of increase in deaths from lung cancer. It was hypothesised that tobacco, particularly when smoked in the form of cigarettes, might be responsible. This led directly	Research review & synthesis	**Key review (conference)**	http://www.mrc.ac.uk/Achievementsimpact/Storiesofimpact/Smoking/index.htm

	to MRC-supported Doll and Hill (1950) study			
1947	43% increase in cigarette tax in the UK leads to 14% reduction in cigarette consumption among men			
1948	Wassink (1948) supports Muller's and Schairer & Schoningen's findings, demonstrating a relationship between smoking and lung cancer in the Netherlands	Discovery	**Publication:**<br>(Wassink, 1948)	
1950	Five key papers published, all describing case control studies showing a link between smoking and lung cancer: Doll and Hill (1950) in the UK; and Wynder and Graham, Schrek et al., Levin et al., and Mills and Porter in the US. The first two were particularly influential	Discovery	**Publications:**<br>(Doll & Hill, 1950)<br>(Levin, Goldstein, & Gerhardt, 1950)<br>(E. Wynder & Graham, 1950)<br>(Mills & Porter, 1950)<br>(Schrek, Baker, Ballard, & Dolgoff, 1950)	
1951	British Doctors Study begins – 40,000 doctors born between 1900 and 1930 tracked (Doll & Hill)	Human research	**Start of data collection (Oct 1951):**<br>Detailed in Doll and Peto (1976)	
1953	Ernst L Wynder finds that painting cigarette tar on the backs of mice creates tumours. This is the first biological link between smoking and cancer.	Discovery	**Publication:**<br>(E. L. Wynder, Graham, & Croninger, 1953)	
1954	Preliminary results of the British Doctors Study published. Doll and Hill suggest an association between smoking and lung cancer and a "significant adjuvant effect" of tobacco on coronary thrombosis.	Discovery	**Publication:**<br>(Doll & Hill, 1954)	
1954	Preliminary results of a large US study of over 187,000 men indicate a higher death rate overall, from coronary artery diseases and from lung cancer among smokers than among non-smokers. The authors also concluded that "probably nicotine is at least partially responsible for the findings in relation to diseases of the coronary arteries."	Discovery	**Publication:**<br>(Hammond & Horn, 1954)	
1954	Reader's Digest publishes an article entitled "The cigarette controversy" documenting the evidence			

	on the association between smoking and lung cancer.			
1955	Cooper and Lindsey (1955) demonstrate existence of carcinogens in cigarette smoke	Discovery	**Publication:**<br>(Cooper & Lindsey, 1955)	
Mid-1950s	In the US, individuals begin to sue tobacco companies for damages			
1956	Doll and Hill publish first results of the British Doctors Study, demonstrating a death rate from lung cancer 20 times higher in those that smoke than those that don't (Doll & Hill, 1956)	Human research	**Publication:**<br>(Doll & Hill, 1956)	
1957	MRC statement that there is a causal relationship between tobacco smoke and cancer	Research review & synthesis	**Key review:**<br>(Medical Research Council, 1957)	
1957	US Surgeon General declares that the official position of the U.S. Public Health Service is that a causal relationship exists between smoking and lung cancer	Research review & synthesis	**Key review**	
1958	First health authority smoking withdrawal clinic opened in Salford	First non-research used in patients	**First use in UK patients in non-research context**	There are likely to be other similar services that this case study was not able to capture in detail
April 1959	RCP sets up Committee "to report on the question of smoking and atmospheric pollution in relation to carcinoma of the lung and other diseases".	Discovery		
1960	Tobacco advertising has increased, primarily driven by cigarette marketing. In 1960, £11m spent on advertising.			(Royal College of Physicians, 1962)
1962	Three-quarters of men and half of women in Britain smoke			(Royal College of Physicians, 1962)
1962	Influential RCP report "Smoking and Health" summarises evidence on smoking causing lung cancer. It also suggests that smoking probably increases the risk of dying from coronary heart disease, and that it might promote the	Research review & synthesis	**Key review:**<br>RCP (Royal College of Physicians, 1962)	

	development and progression of peripheral vascular disease. It recommends advertising restrictions, increased taxation among other things.			
1962	Tobacco Advisory Committee (representing manufacturers) agrees to implement code of advertising practice to make cigarettes seem less glamorous	National policy/guidelines/advice	**Intervention becomes standard practice**	
1964	Influential report published by the US Surgeon General on smoking harms. Over 7,000 papers were reviewed between 1962 and 1964. The report estimated the increased mortality rate and increased risk of lung cancer for smokers compared with non-smokers	Research review & synthesis	**Publication:** (Advisory Committee to the Surgeon General of the Public Health Service, 1964)	
1965	Japanese prospective cohort study on passive smoking begins	Human research	**Start of data collection**	
1965	UK government bans cigarette advertising on TV	National policy/guidelines/advice	**Intervention becomes standard practice**	
1967	1st World Conference on Smoking and Health, New York	Research review & synthesis		
1967	First report on effects of environmental tobacco smoke on children's health	Discovery	**Publication:** (Cameron, 1967)	
1969	Radio Times bans cigarette advertising	National policy/guidelines/advice		
1969	Health Education Council (set up in 1968) launches its first anti-smoking campaign, highlighting risk of lung cancer			
1970	WHO report on 'the limitation of smoking' presented to 23rd World Health Assembly, calling for an end to cigarette advertising and promotion			

	among other recommendations			
1971	Second RCP report 'Smoking and Health Now' published, endorsing 1970 WHO report. ASH report claims caused a permanent 5% drop in cigarette consumption and that a clear divide had emerged whereby professional classes stopped smoking and other did not.	Human research	**Key review:** (Royal College of Physicians, 1971)	
1971	US Surgeon General's report concludes that cigarette smoking is a major risk factor for coronary heart disease in men.	Discovery	**Key review:**	
1971	Action on Smoking and Health (ASH) set up by the RCP as a campaigning public health charity to eliminate harm caused by tobacco.			Is launch of a campaigning group equivalent to first non-research use in patients?
1971	ASH campaigning leads to an increase in provision of non-smoking accommodation on London transport	Intervention becomes standard practice		
1971	First voluntary agreement between government and industry, including warnings on packs and scientific committee established to explore less dangerous forms of smoking. A series of such agreements followed.	National policy/guidelines/advice		
1974	ASH publishes a report calling for more support for smoking cessation clinics. ASH reports no action from health authorities			
1975	Responsibility for Code of Advertising Practice of cigarettes moves from industry to Advertising Standards Authority and it agrees to develop a stricter code.	National policy/guidelines/advice	**Announcement about national policy**	
1975	Gallup poll indicates that two TV documentaries in successive weeks result in 160,000 people (5% if audience) giving up			
1976	BBC smoking cessation campaign 'Stop smoking			

	with Nationwide' runs for several months			
1976	Public opinion: DHSS and NOP poll shows that 70% of the population - a majority of both smokers and non-smokers - favoured further restrictions on smoking in all public places			
1976	Cigarette taxation increased by 3.5p			
1976	Doll and Peto publish 20 year follow up and conclude that between a half and a third of smokers will die because of their smoking	Human research	**Publication:**<br>(Doll & Peto, 1976)	
1977	HEC launches a TV campaign focusing on the rights of non-smokers and smoking by women.			
1977	New voluntary agreement with industry limits marketing and introduction of high tar brands, and strengthens health warnings and advertising restrictions	National policy/guidelines/advice		These agreements are not classified as interventions in standard practice, as it is not clear if they were adhered to and the agreements changed frequently.
1977	Third RCP 'Smoking or Health' report summarises evidence and makes strongest call yet for government action	Research review & synthesis	**Key review:**<br>(Royal College of Physicians, 1977)	
1978	Independent Broadcasting Authority publishes Code of Advertising which terms cigarettes 'unacceptable products' not to be advertised on radio	National policy/guidelines/advice	**Guidelines issued**	
1979	WHO report 'Controlling the smoking epidemic' reviews recent evidence and highlights the risks of passive smoking, as well as socioeconomic effects of smoking	Research review & synthesis	**Publication:**<br>(WHO Expert Committee on Smoking Control, 1979)	
1980	BBC Panorama reports on tobacco industry, highlighting its refusal to acknowledge harms			
1980	BBC Horizon highlights less well known effects of smoking and advantages of giving up			
1981	First epidemiological evidence linking passive smoking with lung cancer – a 10 year prospective	Human research	**Publication:**<br>(Hirayama, 1981)	

	cohort study in Japan. Concluded lung cancer incidence higher among non-smoking women married to smokers than those married to non-smokers.			
1981	The majority of UK people do not smoke			
1981	Cigarette tax increased by 14p, the biggest percentage increase since 1947			
1982	US Surgeon General's report declares cigarette smoking the major cause of cancer mortality in the US and highlights that cessation appears to reduce cancer risk.	Research review & synthesis	**Key review:** (US Department of Health and Human Services, 1982)	
1982	BMA asks the government to ban all forms of tobacco advertising			
1983	Fourth RCP report 'Health or Smoking?' examines health risks of passive smoking, concluding that more than 100,000 people die each year from smoking-related illness in the UK and calling for an end to tobacco advertising and promotion	Research review & synthesis	**Key review:** (Royal College of Physicians, 1983)	
1983	Surgeon General's report states that "smoking-related cardiovascular disease is estimated to account for more deaths than any other smoking-related disease, including cancer", noting that smokers' death rates from coronary heart disease were 70% higher than for non-smokers.	Research review & synthesis	**Key review:** (US Department of Health and Human Services, 1983)	
1984	National No Smoking Day launched in the UK to take place in March every year			
1984	'The Smoke Ring' by campaigning journalist Peter Taylor is published, discussing the politics of the tobacco industry. It is publicised by a BBC Panorama programme aired the same day.			
1985	London Regional Transport bans smoking in underground trains and stations, following a fire at Oxford Circus station, possibly caused by a			

	cigarette			
1985	‘Give Up Smoking’ kit launched by ASH and the Health Education Council for use in GP surgeries			
1985	HEC TV advert campaign highlights that smoking is killing almost as many women as breast cancer			
1985	DHSS issues guidelines asking health authorities to introduce smoking policies in all health premises	National policy/guidelines/advice	**Guidelines issued**	
1985	UK case control study finds, among other things, reduced lung cancer risk among “long-term ex-smokers”	Human research	**Publication:**<br>(Alderson, Lee, & Wang, 1985)	
1986	Protection of Children (Tobacco) Act passes, making it illegal to sell any tobacco product to under-16s (previously only applied to loose tobacco)	National policy/guidelines/advice	**Intervention becomes standard practice**	
1986	BMA publishes “Great Expectorations”, exploring tobacco industry marketing			
1986	BMJ article concluding that passive smoking is a cause of lung cancer. The authors combined data from 13 previous studies, which individually were too small to generate conclusive results.	Human research	**Publication:**<br>(Wald, Nanchahal, Thompson, & Cuckle, 1986)	
1986	Three reports review evidence on passive smoking and conclude that it is a cause of lung cancer	Research review & synthesis	**Publications:**<br>(U.S. Department of Health and Human Services, 1986)<br>(International Agency for Research on Cancer, 1986)<br>(National Research Council, 1986)	
1986	Britain has highest death rate from lung cancer in the world (WHO)			
1987	Independent Television ceases transmission of tobacco-sponsored sport			
1987	Launch of European Commission’s ‘Europe Against Cancer’, a 3 year campaign to raise			

	awareness of risky behaviour			
1988	HEA launches ‘Smoking and Me’, an education programme aimed at 12-13 year olds			
1988	Environmental tobacco smoke linked to heart disease in non-smokers	Human research	**Publications:** (Judson Wells, 1988; Kristensen, 1989)	
1988	Less than one-third of UK adults smoke, but decline has slowed			
1988	Independent Scientific Committee on Smoking and Health publishes the Froggatt Report. Among its conclusions it highlights a 10-30% increased risk of developing lung cancer for non-smokers exposer to other people’s smoke. Recommends that workplaces and public places should be non-smoking where separate provision for non-smokers is not possible.	Research review & synthesis	**Key review:** (Froggatt, 1988)	
1988	In the US, a court awards damages against a tobacco company for a lung cancer death.			
1989	US Surgeon General’s report summarises evidence on harms and possible interventions		**Key review:** (US Department of Health and Human Services, 1989)	
1990	Parents Against Tobacco launched to campaign for legislation to protect children from tobacco. It is a coalition of MPs, TV personalities, activists and the public.			
1990	Telephone advice and counselling service launched by QUIT for people trying to stop smoking	First non-research use in patients	**First use in UK patients**	
1990	European parliament votes in favour of banning tobacco advertising	National policy/guidelines/advice		
1991	Publication of ‘Passive smoking: a health hazard’, summarising research on passive smoking for the general public (by Imperial Cancer Research Fund	Research review & synthesis	**Key review:** (Imperial Cancer Research Fund & Cancer Research Campaign, 1991)	

	and Cancer Research Campaign)			
1991	Review of ten epidemiological studies concludes that passive smoking leads to about a 30% increase in risk of death from ischemic heart disease or myocardial infarction among nonsmokers living with smokers.	Research review & synthesis	**Key review:**<br>(Glantz & Parmley, 1991)	
1991	16p increase in cigarette tax			
1991	European Commissioners call for advertising ban and a major campaign is launched in the UK, led by Doctors for Tobacco Law (29 organisations representing almost all of the UK's doctors). First activity was a demonstration held outside Rothmans' AGM.	National policy/guidel ines/advice		
1991	For the first time health warnings are legally required on tobacco packaging, in line with EC requirements.	National policy/guidel ines/advice	**Intervention becomes standard practice**	
1991	EC directive making TV advertising illegal comes into force	National policy/guidel ines/advice	**Intervention becomes standard practice**	
1991	'From the Billboard to the Playground' highlights evidence on effects of tobacco advertising on children	Research review & synthesis	**Publication:**<br>(Hastings, Aitken, & MacKintosh, 1991)	
1991	Health Education Authority publishes 'The Smoking Epidemic', a survey of deaths from tobacco-related disease and details of costs to the NHS	Human research	**Publication:**<br>HEA (Health Education Authority, 1991)	
1992	MEPs vote in favour of banning tobacco advertising	National policy/guidel ines/advice		
1992	Children and Young Persons (Protection from Tobacco) Act comes into force, tightening previous legislation, including banning sales of single cigarettes.	National policy/guidel ines/advice	**Intervention becomes standard practice**	

1992	13p increase in tobacco duty			
1992	Government White Paper 'The Health of the Nation' published, aiming to reduce prevalence of smoking to 20% by 2000.	National policy/guidelines/advice	**Announcement about national policy**	
1992	The Smee Report on effect of tobacco advertising on consumption, including the effect of advertising bans	Human research	**Key review:**<br>(Smee, 1992)	
1993	Health Education Authority publishes 'The Smoking Epidemic: a Prescription for Change', which puts the annual cost of smoking in terms of GP consultations, prescriptions, and inpatient and outpatient visits at £610m to the NHS in England and Wales	Discovery	**Publication:**<br>(Health Education Authority, 1993)	
1993	'Quitting is Winning' anti-smoking campaign launched in London, targeting parents who smoke			
1994	Government action plan to reduce smoking, outlining action on: price; increasing awareness of risks and supporting people to give up; advertising controls; protection from passive smoking; and improving scientific understanding. A new Scientific Committee on Tobacco and Health is launched.	National policy/guidelines/advice	**Announcement about national policy**	
1994	More than 50 organisations make a joint submission to the Chancellor of the Exchequer, supporting regular tax increases to control tobacco consumption			
1994	Update on the British Doctors Study published, concluding that around half of all smokers will die from smoking related causes	Human Research	**Publication:**<br>(Doll, Peto, Wheatley, Gray, & Sutherland, 1994)	
1994	Government launches three year £13.5m national anti-smoking campaign in England aimed at adults			
1995	Health Education Authority launches 'Put smoking out of fashion' campaign aimed at models and			

	modelling agencies			
1996	Government launches three-year campaign aimed at teenagers			
1996	Guernsey's State Parliament becomes first government in British Isles to impose a complete ban on tobacco advertising, coming into force in 1997	National policy/guidelines/advice		
1997	New Labour government announces its commitment to ban tobacco advertising, and Health Secretary announces that tobaccos advertising of sport will be banned. An Anti-smoking summit is held to discuss ways to reduce smoking.	National policy/guidelines/advice	**Announcement about national policy**	
1997	MINTEL survey suggests a rise in number of smokers in the UK for the first time in 20 years. Contrary to earlier trends, the increase appears to be in the highest socioeconomic groups.<br>Similarly, an ONS survey reveals a 1% increase in smoking prevalence in the 11-15 age group, and the 1996 General Household Survey finds a rise in prevalence among adults for the first time since smoking data was first collected by the government in 1972.			
1997	European Council of Health Ministers votes to ban tobacco advertising throughout the EU.	National policy/guidelines/advice		
1998	Government-appointed Scientific Committee on Tobacco and Health publishes its review on the evidence on passive smoking, concluding that it is a cause of lung cancer	Research review & synthesis	**Key review:**<br>(UKDH, 1998)	
1998	EP votes in favour of EU Directive to ban tobacco advertising and sponsorship, and it is formally adopted by MS.	National policy/guidelines/advice		
1998	Gro Harlem Brundtland, newly elected DG of the	National		

	WHO, calls for worldwide ban on tobacco advertising. WHO sets a target of reducing smoking in Europe to under 20%.	policy/guidelines/advice		
1998	White Paper on first ever national comprehensive tobacco control strategy includes targets to reduce smoking prevalence, launch local NHS smoking cessation services, ban tobacco advertising and promotion, action against tobacco smuggling, increase tobacco taxation by 5% in real terms each year, media campaigns, tougher enforcement on under age sales, and support further restrict smoking in the workplace but only through a voluntary charter.	National policy/guidelines/advice	**Announcement about national policy**	
1999	World Health Assembly backs a resolution to begin work on a new Framework Convention on Tobacco Control (FCTC)	National policy/guidelines/advice		
2000	Evidence from UK trends shows that smoking cessation can greatly reduce lung cancer risk, even among those smoking well into middle age	Human Research	**Publication:** (Peto et al., 2000)	
2000	House of Commons Health Committee report on tobacco industry concludes that in almost every area it is under-regulated or poorly-regulated, and calls for establishment of a Tobacco Regulation Authority.			
2000	UK introduces entitlement to receive behavioural support from trained advisor plus NRT or bupropion on NHS, local cessation services expanded to have national coverage.	National policy/guidelines/advice	**Announcement about national policy**	(West, 2006)
2001	European Court of Justice overturns EU Directive on tobacco advertising, ruling that this is beyond the EU's powers. A new, more limited Directive is published that would ban press and radio advertising, as well as sponsorship of sports events taking place in more than one EU country.	National policy/guidelines/advice		

2002	CRUK launches draft code of practice urging universities and research organisations to reject tobacco industry funding, while itself committing to not funding any institution which also receives industry money.	National policy/guidelines/advice	**Guidelines issued**	
2002	Bill to ban tobacco advertising, which had begun as a Private Member's bill in the House of Lords, is passed in parliament.	National policy/guidelines/advice	**Announcement about national policy**	
2002	EU Directive on tobacco advertising is adopted	National policy/guidelines/advice		
2002	British Medical Association report calls for ban on smoking in public places because of effects of passive smoking.		(Gulland, 2002)	
2003	First phase of Tobacco Advertising and Promotion Act 2002 is implemented, banning advertising on billboards, in print media, direct mail, internet advertising and new promotions.	National policy/guidelines/advice	**Intervention becomes standard practice**	
2003	WHO FCTC adopted by the World Health Assembly, 21 May 2003	National policy/guidelines/advice		http://www.who.int/fctc/about/en/index.html
2003	CMO Liam Donaldson challenges the government to ban smoking in public places in his 2002 Annual Report.			
2004	British Heart Foundation anti-smoking campaign showing fat oozing out of a smoker's artery. BHF reports that the campaign is effective in increasing calls to NHS smoking helpline and visits to website.			
2004	Follow-up results from the British Doctors Study show that, on average, smoking lowered life expectancy by 10 years. Around half of those who smoked were killed by their habit. Stopping smoking at ages 30, 40, 50 and 60 increased life	Human research	**Publication:** (Doll, et al., 2004)	

	expectancy by around ten, nine, six and three years, respectively. (Doll, Peto, Boreham, & Sutherland, 2004)			
2004	Government adviser Derek Wanless publishes 'Securing Good Health for the Whole Population', which recommends banning smoking in work places among other things. This is echoed by the CMO in his 2003 annual report, which concludes that this would bring a net benefit to society of £2.3-2.7bn annually.	Research review & synthesis	**Key review:** (Wanless, 2004)	
2004	A government white paper follows proposing a ban in the majority of workplaces and public places (but short of a total ban).	National policy/guidelines/advice	**Announcement about national policy**	
2004	Scottish FM announces that Scotland will introduce a total ban on smoking in workplaces and public places.	National policy/guidelines/advice	**Announcement about national policy**	
2005	WHO Framework Convention on Tobacco Control (WHO FCTC) reaches the level of ratifications necessary (40) for entry into force, 27 Feb 2005	National policy/guidelines/advice		http://www.who.int/fctc/ten_fctc/en/index.html
2005	Cochrane review of individual behavioural counselling for smoking cessation	Research review & synthesis	**Systematic review** (Cochrane): (T. Lancaster & L. Stead, 2005)	
2005	Cochrane review of self-help interventions for smoking cessation	Research review & synthesis	**Systematic review** (Cochrane): (T. Lancaster & L. F. Stead, 2005)	
2005	Scottish Parliament passes Smoking, Health and Social Care (Scotland) Bill to ban smoking in all workplaces and public places	National policy/guidelines/advice		
2005	Final phase of Tobacco Advertising and Promotion Act 2002 is implemented, banning tobacco sponsorship of global sports. EU Directive banning cross-border advertising and sponsorship comes into effect at the same time (July).	National policy/guidelines/advice	**Intervention becomes standard practice**	

2005	Northern Ireland Minister announces that smoking will be banned in all workplaces by April 2007.	National policy/guidelines/advice	**Announcement about national policy**	
2006	NICE PH intervention guidance PHI1: Brief interventions and referral for smoking cessation in primary care and other settings	National policy/guidelines/advice	**Clinical guideline publication**	
March 2006	Scotland implements smokefree legislation	National policy/guidelines/advice	**Intervention becomes standard practice**	
July 2006	Government issues proposals to raise minimum age for purchase of tobacco to 18 (from 16)	National policy/guidelines/advice	**Announcement about national policy**	
2007	NICE PH intervention guidance PHI5: Workplace health promotion: how to help employees to stop smoking	National policy/guidelines/advice	**Clinical guideline publication**	
March 2007	Chancellor announces that VAT on stop smoking aids will be reduced to 5%	National policy/guidelines/advice		
2007	Wales and Northern Ireland (in April) and England (in July) implement smokefree legislation	National policy/guidelines/advice	**Intervention becomes standard practice**	
Oct 2007	Legal age for purchase of tobacco increases to 18	National policy/guidelines/advice	**Intervention becomes standard practice**	
July 2008	ONS Survey: 80% of Britons support smokefree law.			
2008	NICE public health guidance 10 on smoking cessation services	National policy/guidelines/advice	**Guideline issued**	
2009	General Household Survey 2007 reveals lowest ever number of smokers and a record 59% who have never smoked			

Sept 2009	New tobacco control measures to restrict sales to young people voted through in both Scotland and Westminster. Includes banning vending machines and point of sale display.	National policy/guidelines/advice	**Intervention becomes standard practice**	
March 2010	ASH cost-benefit analysis of increasing tobacco tax suggests that a 5% increase would decrease numbers of smokers by 190,000 and lead to economic benefits of over £270m. A Policy Exchange report also argues for a 5% rise in tobacco duty.	Human research	**Publication:**<br>(ASH, 2010)	Have included in 'human research' because considers an intervention
March 2010	Chancellor raises tobacco duty by 1% above inflation and commits to increase it by 2% above inflation between 2011 and 2014.	National policy/guidelines/advice		
Oct 2010	Association of Public Health Observatories launches Local Tobacco Control Profiles for England – web based tool to provide data on the extent of tobacco use, tobacco related harm, and measures being taken to reduce harm at a local level. It is intended for local authorities.			http://www.tobaccoprofiles.info/
Dec 2010	Government launches Public Health White Paper and commits to consultation on plain packaging of tobacco products	National policy/guidelines/advice	**Announcement about national policy**	
March 2011	Government launches Tobacco Plan for England, committing to reducing adult prevalence to 18.5% by 2015	National policy/guidelines/advice	**Announcement about national policy**<br>(Department of Health, 2011)	
2012	Chancellor raises tobacco duty by 5% above inflation	National policy/guidelines/advice		
April-Aug 2012	Public consultation on plain packaging, with more than 200,000 responses. YouGov find 62% of adults in England are in favour.	Human research		
Oct 2012	DH launches 'Stoptober', the first mass smoking cessation attempt.	First non-research use in patients		

2012	First protocol to the WHO FCTC adopted - the Protocol to Eliminate Illicit Trade in Tobacco Products	National policy/guidelines/advice		http://www.who.int/fctc/ten_fctc/en/index.html
2012	DH launches £2.7m 9-week media campaign showing a tumour growing on a cigarette.			
2013	WHO FCTC current membership of 176 countries, covering 90% of the world's population	National policy/guidelines/advice		http://www.who.int/fctc/ten_fctc/en/index.html

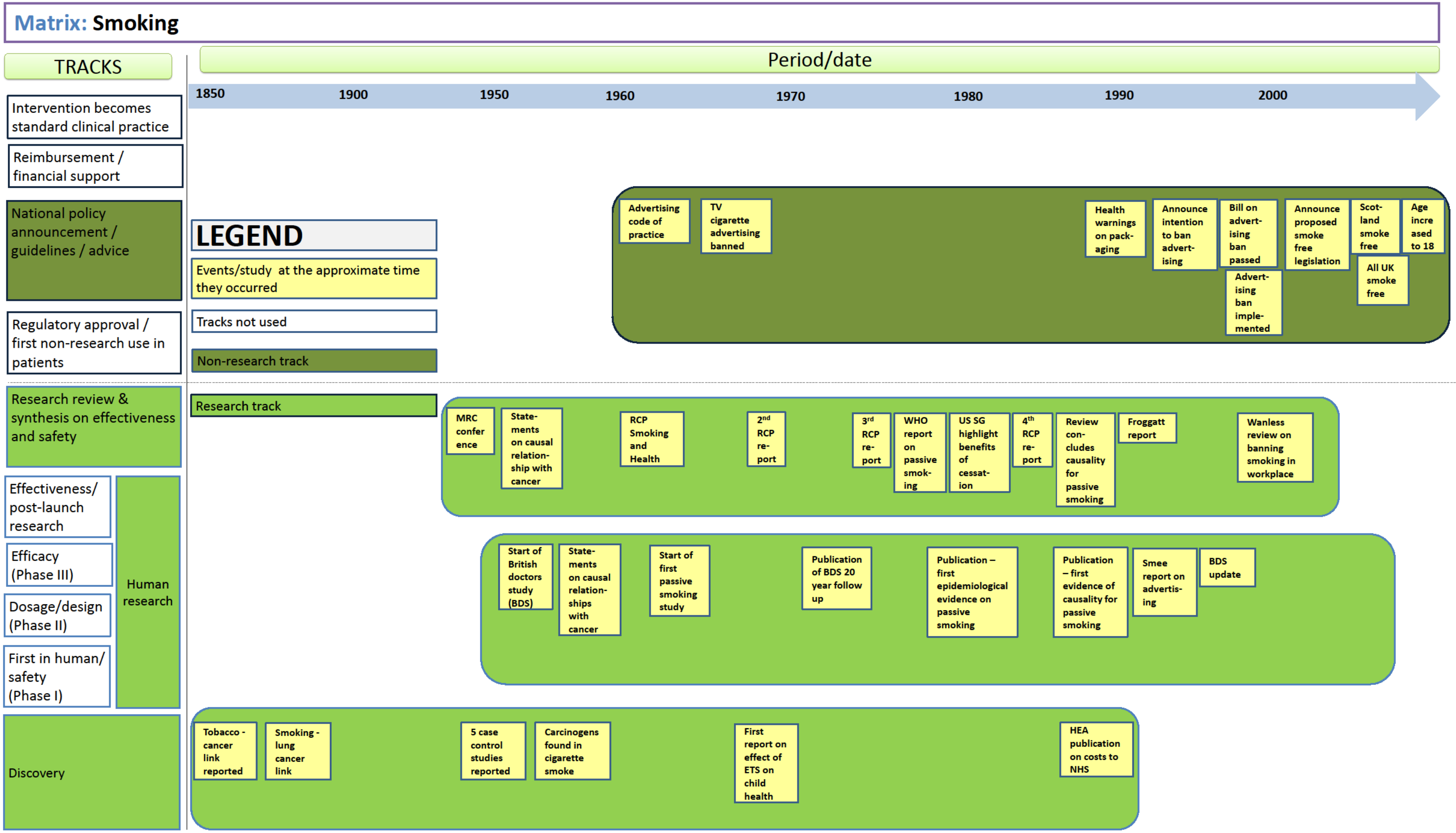
Matrix: Smoking
TRACKS
Period/date
1850
1900
1950
1960
1970
1980
1990
2000
Intervention becomes standard clinical practice
Reimbursement / financial support
National policy announcement / guidelines / advice
Regulatory approval / first non-research use in patients
Research review & synthesis on effectiveness and safety
Effectiveness/ post-launch research
Efficacy (Phase III)
Dosage/design (Phase II)
First in human/ safety (Phase I)
Human research
Discovery
LEGEND
Events/study at the approximate time they occurred
Tracks not used
Non-research track
Research track
Advertising code of practice
TV cigarette advertising banned
Health warnings on pack-aging
Announce intention to ban advert-ising
Bill on advert-ising ban passed
Advert-ising ban imple-mented
Announce proposed smoke free legislation
Scot-land smoke free
All UK smoke free
Age incre ased to 18
MRC confer ence
State-ments on causal relation-ship with cancer
RCP Smoking and Health
2nd RCP re-port
3rd RCP re-port
WHO report on passive smok-ing
US SG highlight benefits of cessat-ion
4th RCP re-port
Review con-cludes causality for passive smoking
Froggatt report
Wanless review on banning smoking in workplace
Start of British doctors study (BDS)
State-ments on causal relation-ships with cancer
Start of first passive smoking study
Publication of BDS 20 year follow up
Publication – first epidemiological evidence on passive smoking
Publication – first evidence of causality for passive smoking
Smee report on advertis-ing
BDS update
Tobacco - cancer link reported
Smoking - lung cancer link
5 case control studies reported
Carcinogens found in cigarette smoke
First report on effect of ETS on child health
HEA publication on costs to NHS

METHODOLOGICAL REFLECTIONS:

- The main challenge of this case study is that it is not looking at one definable intervention, since there are numerous approaches to reducing smoking and they are not easily separable. This has affected the scope (as explained below) and means that different 'interventions' are intertwined in the timeline table. These are classified into different categories and colour coded in the table as indicated in the key.
- Interventions that are commercially developed are not included (e.g. drugs, stop smoking aids).
- Evidence on harms other than cardiovascular disease (the focus of the study) and cancer (the main driver of policy and interventions relating to smoking) was not included to keep the scope manageable.
- While the case study includes research evidence internationally, it focuses on intervention in the UK. This is mostly restricted to national level policies, campaigns and activities, as it was not possible to capture everything that has taken place locally or regionally.
- Discovery/human research is defined as follows: harms evidence is classified as discovery unless a comparison with a non-smoking group is used – the logic being that in these cases not smoking is effectively the 'intervention'. Any research looking at a specific intervention is classified as human research.
- Subcategories within human research have not been used, as these were not easily definable in this case study.
- Evidence on effectiveness of the various 'interventions' is rarely included, as in an attempt to constrain the scope, the case study focus has been on evidence of harms and details on implementation. In some instances, the evidence on effectiveness is either not specific to smoking/public health (e.g. on effects of taxation) or only emerged following the implementation of an 'intervention' as its effectiveness was assessed. In this latter case, effectiveness evidence will have had little/no effect on the time lag.
- An important aspect of this case study is awareness raising – e.g. campaigns, media coverage, information provision. This does not fit easily into our 'track' structure. Campaigns can be classed as interventions, but this is less clear for events such as a TV documentary on the tobacco industry or a statement by a lobbying group.
- In some cases, it is difficult to distinguish between tracks e.g. in cases where the national policy is the intervention, such as increasing tobacco duty. For this reason some 'events' have not been classified into tracks.
- Calibration points – the following definitions are used:
    - Announcement about national policy – a government statement of intent (e.g. commit to reducing smoking, or to implement a ban in future)
    - Guidelines issued – when non-compulsory guidelines or codes of practice are launched (e.g. around advertising)
    - Intervention becomes standard practice – when a policy or regulation comes into force and is compulsory (this is the same as a national policy being implemented, but by definition it becomes standard practice immediately if it is a law)

REFERENCES

Adler, I. (1912). *Primary malignant growths of the lungs and bronchi*: Longmans, Green, and Co.

Advisory Committee to the Surgeon General of the Public Health Service. (1964). *Smoking and Health: Report of the Advisory Committee to the Surgeon General of the United States*. Washington, D.C.: US Government Printing Office.

Alderson, M. R., Lee, P. N., & Wang, R. (1985). Risks of lung cancer, chronic bronchitis, ischaemic heart disease, and stroke in relation to type of cigarette smoked. *Journal of Epidemiology and Community Health, 39*(4), 286-293.

ASH. (2010). *The Effects of Increasing Tobacco Taxation: A Cost Benefit and Public Finances Analysis*.

Buerger, L. (1908). Thrombo-angiitis obliterans: a study of the vascular lesions leading to presenile spontaneous gangrene. *The American Journal of the Medical Sciences, 136*(4), 567-580.

Cameron, P. (1967). The presence of pets and smoking as correlates of perceived disease. *Journal of Allergy, 40*(1), 12-15.

Cooper, R., & Lindsey, A. (1955). 3: 4-Benzpyrene and other polycyclic hydrocarbons in cigarette smoke. *British journal of cancer, 9*(2), 304.

Department of Health. (2011). *Healthy Lives, Healthy People: A Tobacco Control Plan for England*. London: DH.

Doll, R. (1998). Uncovering the effects of smoking: historical perspective. *Statistical Methods in Medical Research, 7*(2), 87-117.

Doll, R., & Hill, A. B. (1950). Smoking and carcinoma of the lung. *British medical journal, 2*(4682), 739-748.

Doll, R., & Hill, A. B. (1954). The mortality of doctors in relation to their smoking habits. *British medical journal, 1*(4877), 1451.

Doll, R., & Hill, A. B. (1956). Lung cancer and other causes of death in relation to smoking. *British medical journal, 2*(5001), 1071-1081.

Doll, R., & Peto, R. (1976). Mortality in relation to smoking: 20 years' observations on male British doctors. *British medical journal, 2*(6051), 1525.

Doll, R., Peto, R., Boreham, J., & Sutherland, I. (2004). Mortality in relation to smoking: 50 years' observations on male British doctors. *Journal of Epidemiology and Community Health, 58*(11), 930-930.

Doll, R., Peto, R., Wheatley, K., Gray, R., & Sutherland, I. (1994). Mortality in relation to smoking: 40 years' observations on male British doctors. *BMJ: British Medical Journal, 309*(6959), 901.

English, J. P., Willius, F. A., & Berkson, J. (1940). Tobacco and coronary disease. *Journal of the American Medical Association, 115*(16), 1327-1329.

Erb, W. (1904). Ueber dysbasia angiosclerotica ("intermittierendes Hinken"). *Münch Med Wochenschr, 51*, 905-908.

Froggatt, P. (1988). *Fourth report of the independent scientific committee on smoking & health*: HMSO.

Glantz, S. A., & Parmley, W. W. (1991). Passive smoking and heart disease. Epidemiology, physiology, and biochemistry. *Circulation, 83*(1), 1-12.

Gulland, A. (2002). BMA steps up call for ban on smoking in public places. *BMJ: British Medical Journal, 325*(7372), 1058.

Hammond, E. C., & Horn, D. (1954). The relationship between human smoking habits and death rates. *JAMA, 155*(15), 1316-1328.

Hastings, G., Aitken, P., & MacKintosh, A. (1991). *From the Billboard to the Playground*. Glasgow: University of Strathclyde.

Health Education Authority. (1991). The smoking epidemic. *The Lancet, 338*(8779), 1387.

Health Education Authority. (1993). *The smoking epidemic. A prescription for change*. London: HEA.

Hirayama, T. (1981). Non-smoking wives of heavy smokers have a higher risk of lung cancer. *British Medical Journal (Clinical research ed.), 283*(6296), 916.

Howard, T. (1934). Coronary occlusion, based on the study of 165 cases. *Med. Times and Long Island Med. Jr*, 337-341.

Huchard, H. (1893). *Traité clinique des maladies du coeur et des vaisseaux* (2nd ed.). Paris: Doin.

Imperial Cancer Research Fund, & Cancer Research Campaign. (1991). *Passive smoking: a health hazard*. London: ICRF/CRC.

International Agency for Research on Cancer. (1986). *IARC monographs on the evaluation of the carcinogenic risk of chemicals to man. v. 38, Tobacco smoking*. Lyon, France: World Health Organization.

Jacobs, F. (1855). On Cancer of the Lip. *The Boston Medical and Surgical Journal, 53*(1), 13-14.

Judson Wells, A. (1988). An estimate of adult mortality in the United States from passive smoking. *Environment International, 14*(3), 249-265.

Kristensen, T. S. (1989). Cardiovascular diseases and the work environment: a critical review of the epidemiologic literature on chemical factors. *Scandinavian journal of work, environment & health*, 245-264.

Lancaster, T., & Stead, L. (2005). Individual behavioural counselling for smoking cessation. *Cochrane Database Syst Rev, 2*.

Lancaster, T., & Stead, L. F. (2005). Self-help interventions for smoking cessation. *Cochrane Database Syst Rev, 3*(3).

Levin, M., Goldstein, H., & Gerhardt, P. (1950). Cancer and tobacco smoking: A preliminary report. [doi: 10.1001/jama.1950.02910390008002]. *Journal of the American Medical Association, 143*(4), 336-338.

Medical Research Council. (1957). Tobacco Smoking and Lung Cancer. *BMJ, 1*(5034), 1523-1524.

Mills, C. A., & Porter, M. M. (1950). Tobacco smoking habits and cancer of the mouth and respiratory system. *Cancer research, 10*(9), 539-542.

Müller, F. (1939). Tabakmissbrauch und lungencarcinom. *Zeitschrift für Krebsforschung, 49*(1), 57-85.

National Research Council. (1986). *Environmental tobacco smoke: measuring exposures and assessing health effects*. Washington, D.C.

Peto, R., Darby, S., Deo, H., Silcocks, P., Whitley, E., & Doll, R. (2000). Smoking, smoking cessation, and lung cancer in the UK since 1950: combination of national statistics with two case-control studies. *BMJ, 321*(7257), 323-329.

Proctor, R. (2000). *The Nazi war on cancer*: PRINCETON University Press.

Proctor, R. N. (2012). The history of the discovery of the cigarette–lung cancer link: evidentiary traditions, corporate denial, global toll. *Tobacco Control, 21*(2), 87-91.

Royal College of Physicians. (1962). *Smoking and Health*. London: Royal College of Physicians.

Royal College of Physicians. (1971). *Smoking and Health Now*. London: Royal College of Physicians.

Royal College of Physicians. (1977). *Smoking or Health*. London: Royal College of Physicians.

Royal College of Physicians. (1983). *Health or Smoking?* London: Royal College of Physicians.

Schairer, E., & Schöniger, E. (1944). Lungenkrebs und tabakverbrauch. *Zeitschrift für Krebsforschung, 54*(4), 261-269.

Schrek, R., Baker, L. A., Ballard, G. P., & Dolgoff, S. (1950). Tobacco smoking as an etiologic factor in disease. I. Cancer. *Cancer research, 10*(1), 49-58.

Shew, J. (1854). *Prize Essay: Tobacco Diseases, with a Remedy for the Habit*: Fowlers and Wells.

Smee, C. (1992). *Effect of Tobacco Advertising on Tobacco Consumption (The Smee Report)*. London: DH.

U.S. Department of Health and Human Services. (1986). *The health consequences of involuntary smoking. A report of the Surgeon General.* Washington, D.C.: U.S. DHHS, Public Health Service, Office of the Assistant Secretary for Health, Office of Smoking and Health.

UKDH. (1998). *Report of the Scientific Committee on Tobacco and Health*. London: Department of Health (UK), Department of Health And Social Services, Northern Ireland, The Scottish Office Department of Health, Welsh Office.

US Department of Health and Human Services. (1982). *The Health Consequences of Smoking: Cancer: A Report of the Surgeon General*: United States. Public Health Service. Office on Smoking and Health.

US Department of Health and Human Services. (1983). *The Health Consequences of Smoking: Cardiovascular Disease: A Report of the Surgeon General.*: United States Public Health Service.

US Department of Health and Human Services. (1989). *Reducing the Health Consequences of Smoking: 25 Years of Progress. A Report of the Surgeon General.* .

Wald, N. J., Nanchahal, K., Thompson, S. G., & Cuckle, H. S. (1986). Does breathing other people's tobacco smoke cause lung cancer? *British Medical Journal (Clinical research ed.), 293*(6556), 1217.

Wanless, D. (2004). *Securing good health for the whole population*. London: HM Treasury.

Wassink, W. F. (1948). Onstaansvoorwasrden voor Longkanker. *Ned Tijdschr Geneeskd, 92*, 3732-3747.

WHO Expert Committee on Smoking Control. (1979). *Controlling the Smoking Epidemic: Report of the WHO Expert Committee on Smoking Control*: World Health Organization.

Wynder, E., & Graham, E. (1950). Tobacco smoking as a possible etiologic factor in bronchogenic carcinoma. *JAMA, 143*, 329-336.

Wynder, E. L., Graham, E. A., & Croninger, A. B. (1953). Experimental Production of Carcinoma with Cigarette Tar. *Cancer research, 13*(12), 855-864.

Time lags between conducting medical research and its translation. Case Study 5: CBT and depression

NARRATIVE ACCOUNT OF BACKGROUND/ DEFINITIONS AND KEY ASPECTS OF THE INTERVENTION'S DEVELOPMENT

Definition of CBT

It is generally accepted that 'CBT' refers to a 'family of allied therapies that draw on a common base of behavioural and cognitive models of psychological disorders and utilise a set of overlapping techniques' (Churchill et al., 2012). The full NICE guidelines on depression define CBT as:

> *… time-limited, structured psychological interventions, derived from the cognitive behavioural model of affective disorders and where the patient:*
>
> - *works collaboratively with the therapist to identify the types and effects of thoughts, beliefs and interpretations on current symptoms, feelings states and/or problem areas*
> - *develops skills to identify, monitor and then counteract problematic thoughts, beliefs and interpretations related to the target symptoms/problems*
> - *learns a repertoire of coping skills appropriate to the target thoughts, beliefs and/or problem areas.*
>
> *In most individual trials of CBT, the manual used was Beck's Cognitive Therapy of Depression (1979) which advocates 16 to 20 sessions for treatment and relapse prevention work.*

The development of CBT to treat depression

Throughout the 1960s and 1970s A.T. Beck wrote extensively on the connection between depression, cognitive distortions and the use of CBT to treat depression, which led to the first RCT being carried out in 1977 (categorised as a phase III study in the timeline), and CBT being developed for the treatment of depression in 1979. Between 1977 and 1998 78 RCTs were conducted in this area, and in 1989 the first meta-analysis was published.

However, it wasn't until 2000 that a series of cost-benefit studies were undertaken, which is thought to have unlocked funding to roll out the implementation of CBT across the NHS. The full NICE depression guidelines (2009), describe the development of CBT as follows:

the development work took place in the 1960s and 1970s; the manual was published in 1979 (Beck et al., 1979); the first RCTs were published in the late 1970s and early 1980s (Rush et al., 1977; Kovacs et al., 1981; Rush et al., 1981); the first meta-analysis was conducted in 1990 (Robinson et al.,1990); and the effectiveness and cost-effectiveness studies have only started to emerge in the last decade (Bower et al., 2000; Byford et al., 2003; Scott et al., 2003). In summary, over the past 50 years there has been a significant expansion of theories and therapies for depression. However, only a relatively small number of these therapies have travelled the full empirical road and demonstrated that they are efficacious and can be cost-effective treatment options for the NHS.

The use of CBT to treat depression was recommended in the Department of Health's 'Treatment Choice in Psychological Therapies and Counselling' in 2001 and in 2004 the first NICE guidance on depression was published which also recommended CBT for the treatment of depression. However, despite these recommendations CBT was not widely available given the lack of qualified CBT therapists. In 2006 the Improving Access to Psychological Therapies (IAPT) initiative was launched which from the outset 'recognised that a national shortage of CBT practitioners, who are skilled in helping people recover from depression and anxiety disorders, was the core deficiency preventing routine NHS delivery of the NICE guidelines' (Improving Access to Psychological Therapies, 2006). As part of the IAPT initiative in 2008 Health Secretary Alan Johnson announced an extra 3,600 therapists would be trained to provide CBT treatment on the NHS at a cost of £173 million per year from 2010. The scope of CBT and computerised CBT (CCBT) was broadened in the NICE 2009 depression guidelines which suggest that CBT (either alone or in combination with medication) should be used for mild, moderate and severe cases of depression. The 2004 guidelines had stated that there was insufficient evidence to introduce CCBT technology into the NHS.

In 2011 the Department of Health released 'Talking Therapies: A four-year plan of action' which underscored how important the economic case was in rolling out the use of CBT across the NHS. It stated that '*the evidence that proved CBT is as effective as medication in helping people with depression and anxiety disorders – and better at preventing relapse – led to the economic case that secured annual funding to begin the national roll-out in the three years to March 2011. Key to the economic case was an argument that effective therapeutic interventions combined with employment support could reduce the numbers of people on sick pay and benefits'.*

TIMELINE

All dates in the period/date column refer to publication dates. Publication dates have also been used to draw the matrix.

Period/date	Study/Event	Track	Calibration point	Comments
1962	Seminal study on rational emotive behaviour therapy (REBT) – an early form of CBT. (A. Ellis) (book)	Discovery	**Publications:**<br>Ellis, A. (1962) Reason and Emotion in Psychotherapy, Lyle Stewart: New York<br><br>**Reviews:**<br>Cited in Cochrane Review Protocol (Churchill et al., 2012)<br><br>**Guidelines:**<br>Cited in full NICE depression guidance (2009)	
1977-1998	78 randomised controlled clinical trials regarding the use of CBT and depression published (see Gloaguen, et al., 1998)	Narrative	N/A	
1960s and 1970s	Beck wrote extensively on the connection between depression, cognitive distortions and the use of cognitive therapy as treatment	Discovery	**Publications:**<br>Beck, A. T. Thinking and depression: I. Idiosyncratic content and cognitive distortions. Archives of General Psychiatry, 1963, 9, 324-333.<br>Beck, A. T. Thinking and depression: II. Theory and therapy. Archives of General Psychiatry, 1964, 10, 561-571.<br>Beck, A. T. Depression: Clinical experimental, and theoretical aspects. New York: Hoeber, 1967. (Republished as Depression: Causes and treatment. Philadelphia: University of Pennsylvania Press, 1972.)<br>Beck, A. T. Cognitive therapy and the emotional disorders. New York: International Universities Press, 1976.<br>Beck, A. T., & Beamesderfer, A. Assessment of depression: The depression inventory. In P. Pichot (Ed.), Modern problems in pharmacopsychiatry (Vol. 7). Basel, Switzerland: Karger, 1974.<br>A. T., & Shaw, B. F. Cognitive approaches to depression. In A. Ellis & R. Grieger (Eds.), Handbook of rational emotive theory and practice. New York: Springer, 1977.	
1977	First RCT of CT in depression carried out (Rush et al., 1977) n=41 (n=19 cognitive therapy,	Human research – efficacy (phase III)	**Publications:**<br>Rush, A.J., Beck, A.T., Hollon, S.D. et al., (1977) Comparative efficacy of cognitive therapy and pharmacotherapy in the treatment of depressed	

	n=22 imipramine). NIMH Grant MH-19989-06, a grant from the National Association of Mental Health, and NIMH Grant MH-27759-01.		outpatients. Cogn. Ther. Res. 1 (1), 17-37.<br><br>**Guidelines:**<br>Cited in full NICE depression guidance	
1979	CBT developed for treatment of depression (Beck, A.T. (1979) Cognitive Therapy of Depression, Guilford Press, NY)		**Publications:**<br>Beck, A.T. (1979) Cognitive Therapy of Depression, Guilford Press, NY<br><br>**Guidelines:**<br>Cited in full NICE depression guidance (2009)<br><br>**Reviews:**<br>Cited in Cochrane Review Protocol (Churchill et al., 2012)	
1981	Kovacs et al. conduct an RCT which compares the use of CT with pharmacotherapy for depressed patients and find that *'the cognitive-therapy patients showed greater symptomatic improvement and a higher treatment-completion rate. A one-year naturalistic follow-up of the 35 subjects who completed the protocol revealed that although many of the patients had a variable clinical course, both original treatment groups remained generally well. Self-rated depressive symptomatology was significantly lower for those who, one year earlier, had completed cognitive therapy than for those who had been in the clinical trial's pharmacotherapy cell'.*	Human research – efficacy (phase III)	**Publications:**<br>Kovacs, M., Rush, A. T., Beck, A. T., et al. (1981) Depressed outpatients treated with cognitive therapy or pharmacotherapy: a one-year follow-up. Archives of General Psychiatry, 38, 33–39.<br><br>**Guidelines:**<br>Cited in full NICE depression guidance (2009)<br>http://www.nice.org.uk/nicemedia/live/12329/45896/45896.pdf<br><br>N.B. not mentioned in Cochrane Review Protocol (Churchill et al, 2012)	

	N=44. This study was supported by National Institute of Mental Health (NIMH) grant MH-30847, a grant from the National Association of Mental Health, and the Center for Cognitive Therapy and Research, Philadelphia. The data analyses and preparation of this report were supported by NIMH grants MH-30915-01 and MH-35420-03.			
1981	Rush et al. conduct an RCT which compares cognitive therapy with pharmacotherapy and finds that '*[d]uring cognitive therapy improvements in hopelessness, views of the self and mood generally preceded changes in vegetative and motivational symptoms. On the other hand, no consistent pattern of change was associated with pharmacotherapy. While these results are compatible with the notion that cognitive therapy initially alters negative thinking and mood, which secondarily leads to improvements in vegetative and motivational symptoms, further studies with a placebo or wait-list group are needed to determine if this is a unique effect of cognitive therapy.*'<br><br>N=35. This study was supported	Human research – efficacy (phase III)	**Publications:**<br>Rush, A. J., Kovacs, M., Beck, A. T., et al. (1981) Differential effects of cognitive therapy and pharmacotherapy on depressive symptoms. Journal of Affective Disorders, 3, 221–229.<br><br>**Guidelines:**<br>Cited in full NICE depression guidance<br>http://www.nice.org.uk/nicemedia/live/12329/45896/45896.pdf<br><br>N.B. not mentioned in Cochrane Review Protocol (Churchill et al, 2012)	

	in part by grants from the National Institute of Mental Health to A.J. Rush (MH-28459) and A.T. Beck (MH-19989-06).			
1989	Key large well-controlled clinical trial – conducted by the Treatment of Depression Collaborative Research Program, the study compared the efficacy of CBT, interpersonal psychotherapy, and pharmacotherapy for depression. Outcomes for CBT and interpersonal therapy were generally equivalent (Elkin et al., 1989) n=250, funded by NIMH	Human research – efficacy (phase III)	**Publications:**<br>Elkin, I., Shea, M.T., Watkins, J.T. et al. (1989) National Institute of Mental Health Treatment of Depression Collaborative Research Program. General effectiveness of treatments. Arch Gen Psychiatry. 1989 Nov; 46(11):971-82<br><br>Pilot phase began in July 1980<br>Study initiated in 1982 after completion of pilot phase<br>1979 research protocol<br>1980 revised research plan<br><br>**Guidelines:**<br>Cited in full NICE guidance<br>http://www.nice.org.uk/nicemedia/live/12329/45896/45896.pdf<br><br>N.B. not mentioned in Cochrane Review Protocol (Churchill 2012)	
1989	Dobson et al. (1989) conduct first meta-analysis on the use of CBT in treating depression and found that CBT is superior to untreated controls, wait list, pharmacotherapy, behaviour therapy, and a heterogeneous group of other therapies.	Research review and synthesis on effectiveness	**Publications:**<br>Dobson, K.S. (1989). A meta-analysis of the efficacy of cognitive therapy of depression. *Journal of Consulting and Clinical Psychology,* 57, 414-419.	
1990	Robinson et al. conducted a review and found that '*depressed clients benefit substantially from psychotherapy, and these gains appear comparable to those observed with pharmacotherapy*'.	Research review and synthesis on effectiveness	**Publications:**<br>Robinson, L. A., Berman, J. S. & Neimeyer, R. A. (1990) Psychotherapy for the treatment of depression: a comprehensive review of controlled outcome research. Psychological Bulletin, 108, 30–49<br><br>**Guidelines:**<br>Cited in full NICE depression guidelines	
1994	A second key large well-	Human research –	**Publications:**	

	controlled clinical trial (Shapiro, 1994) n=117	efficacy (phase III)	Shapiro, D.A., Barkham, M., Rees, A. et al. (1994) Effects of treatment duration and severity of depression on the effectiveness of cognitive-behavioural and psychodynamic-interpersonal psychotherapy. Journal of Consulting and Clinical Psychology, 62, 522-534.<br><br>**Guidelines:**<br>Cited in 'Treatment Choice in Psychological Therapies and Counselling' (2001) as evidence for using CBT to treat depression and full NICE depression guidelines	
1998	Gloaguen et al. conduct a meta-review	Research review and synthesis on effectiveness	**Publications:**<br>Gloaguen, V., Cottraux, J. Cucherat, M. and Blackburn, I. (1998). A meta-analysis of the effects of cognitive therapy in depressed patients. Journal of Affective Disorders, 49, 59-72.<br><br>**Review:**<br>Cited in Cochrane Review Protocol (Churchill et al., 2012)<br><br>N.B. not in full NICE depression guidance	
2000	King et al (2000) *'reported results of a pragmatic controlled trial of 464 patients (of whom 197 were randomised between usual GP care, non-directive counselling and cognitive behaviour therapy, 137 chose a specific therapy and 130 were randomised between the two psychological therapy conditions). They found counselling and cognitive behaviour therapy were equally effective and superior to usual GP treatment for both depression and mixed anxiety/depression at 4 months. By one year, the control group had improved to be equivalent to the psychological*	Human research – efficacy (phase III)	**Publications:**<br>King M, Sibbald B, Ward E, Bower P, Lloyd M, Gabbay M, Byford S. (2000) Randomised controlled trial of non-directive counselling, cognitive-behaviour therapy and usual general practitioner care in the management of depression as well as mixed anxiety and depression in primary care. The National Coordinating Centre for Health Technology Assessment (NCCHTA) (Vol.4: No.19): 83.<br><br>Patients recruited between February 1996 and November 1997<br><br>Response in 1993(?) to HTA programme call 93/07<br><br>**Guidelines:**<br>Cited in 'Treatment Choice in Psychological Therapies and Counselling' (2001) as evidence for recommending CBT for the treatment of depression.<br><br>Cited in 'The Nice Guideline on the Treatment and Management of Depression in Adults' and full NICE guidelines	

	therapy groups. At 12 months, the patients who had received non-directive counselling expressed higher levels of satisfaction than the other two groups.'			
2000-2003	Key studies highlighting the cost-benefit of the use of CBT in treating depression<br><br>Cross-link with launch of IAPT – the economic case led to annual funding to begin the national roll-out of CBT from 2008-2011.<br><br>Bower et al. found that *'both brief psychological therapies [non-directive counselling and CBT] may be significantly more cost effective than usual care in the short term, as benefit was gained with no significant difference in cost. There are no significant differences between treatments in either outcomes or costs at 12 months.'*	Human research – effectiveness/ post-launch research	**Publications:**<br>Bower, P., Byford, S., Sibbald, B., et al. (2000) Randomised controlled trial of nondirective counselling, cognitive-behaviour therapy, and usual general practitioner care for patients with depression. II: Cost-effectiveness. British Medical Journal, 321, 1389–1392<br><br>Patients recruited February 1996 to November 1997<br><br>Byford, S., Knapp, M., Greenshields, J., et al. (2003) Cost-effectiveness of brief cognitive behaviour therapy versus treatment as usual in recurrent deliberate self-harm: a decision-making approach. Psychological Medicine, 33, 977–986<br><br>Scott, J., Palmer, S., Paykel, E., et al. (2003) Use of cognitive therapy for relapse prevention in chronic depression. Cost-effectiveness study. British Journal of Psychiatry, 182, 221–227.<br><br>**Guidelines:**<br>Cited in full NICE guidelines	Cannot find other calibration points.
2001	Department of Health publishes 'Treatment Choice in Psychological Therapies and Counselling' which draws on 8 high quality reviews and 2 Cochrane reviews to conclude that the 'best evidence' for effective psychological therapy for depression is for cognitive behavioural therapy.	National policy announcement/ guidelines/ advice	**Guidelines:**<br>Department of Health 'Treatment Choice in Psychological Therapies and Counselling'	

2004	First NICE guidance on depression published (amended in 2007 and updated again in 2010 – current version of guidelines) states that CBT should be used for mild depression. However it states that there is insufficient evidence to introduce CCBT technology into the NHS.	National policy announcement/ guidelines/ advice	**Guidelines:**<br>Full NICE depression guidance (2004)	This point marks the 'launch' of CBT. All RCTs past this point will be categorised as 'human research – effectiveness/ post-launch research'
2005	Major high quality controlled trial comparing CT with a commonly prescribed serotonin reuptake inhibitor (paroxetine) found that cognitive therapy was equally effective for the initial treatment of moderate to severe depression (DeRubeis et al, 2005)	Human research – effectiveness/ post-launch research	**Publications:**<br>DeRubeis, R. J., Hollon, S. D., Amsterdam, J. D., et al. (2005) Cognitive therapy versus medications in the treatment of moderate to severe depression. Archives of General Psychiatry, 62, 409–416<br>(submitted for publication 12 Aug 2003) Could not find any other calibration points<br><br>**Guidelines:**<br>Cited in full NICE guidelines<br>http://www.nice.org.uk/nicemedia/live/12329/45896/45896.pdf	
2006	Improving Access to Psychological Therapies launched by Department for Health with a focus on adults of working age (although in 2010 it was broadened to adults of all ages)<br><br>*'From the outset it recognised that a national shortage of cognitive behavioural therapy (CBT) practitioners, who are skilled in helping people recover from depression and anxiety disorders, was the core deficiency preventing routine NHS delivery of the NICE guidelines'*	National policy announcement/ guidelines/ advice	N/A	

2008	Health Secretary Alan Johnson announces an extra 3,600 therapists will be trained to provide CBT treatment on the NHS at a cost of £173 million per year from 2010 (as part of IAPT).	Reimbursement/ financial support	N/A	
2008	Leading psychotherapy experts claim the superiority of CBT is a myth at a conference at UEA on 7 July 2008.	Narrative	N/A	
2008	Ekers et al. (2008) undertook a systematic review of 17 RCTs and found CBT and behavioural therapy to be equivalent in terms of depression recovery rates, symptom levels and participant dropout.	Research review and synthesis on effectiveness	**Publications:**<br>Ekers, D., Richards, D., Gillbody, S. (2008) 'A meta-analysis of randomized trials of behavioural treatment of depression'. Psychological Medicine, 38(5): 611-23<br><br>**Reviews:**<br>Cited in Cochrane Review Protocol (Churchill et al., 2012)	
2008	Stiles (2008) compared CBT, person-centred therapy (PCT) and psychodynamic therapy (PDT) and found that they all had equivalent outcomes.	Human research – effectiveness/ post-launch research	**Publications:**<br>Stiles, W.B., Barkham, M., Mellor-Clark, J. and Connell, J. (2008) 'Effectiveness of cognitive-behavioural, person-centred and psychodynamic therapies in UK primary-care routine practice: replication in a larger sample'. Psychological Medicine, 38. 677-688<br><br>**Reviews:**<br>Cited in Cochrane Review Protocol (Churchill, 2012)<br>**Guidelines:**<br>Cited in full NICE depression guidelines	
2009	NICE guidelines suggest CBT or CCBT should be used for mild to moderate depression, CBT in combination with antidepressant medication should be used for moderate to severe depression and CBT should be used for patients at risk of relapse into depression	National policy accouncement/ guidelines/ advice	**Guidelines:**<br>NICE depression guidelines 2009	

2011	‘Talking Therapies: A four-year plan of action’ released by the Department of Health.<br><br>*‘The aim is to develop talking therapies services that offer treatment for depression and anxiety disorders approved by the National Institute for Health and Clinical Excellence (NICE) across England by March 2015, the end of the Spending Review period’.*<br><br>The plan states that ‘*the evidence that proved CBT is as effective as medication in helping people with depression and anxiety disorders – and better at preventing relapse – led to the economic case that secured annual funding to begin the national roll-out in the three years to March 2011. Key to the economic case was an argument that effective therapeutic interventions combined with employment support could reduce the numbers of people on sick pay and benefits*’.	National policy announcement	N/A	
2012	Cochrane Review protocol entitled ‘Cognitive behavioural therapies versus other psychological therapies for depression’ to compare the comparative effectiveness and acceptability of CBT interventions	Research review and synthesis on effectiveness	**Review:**<br>Cochrane Review Protocol Churchill R, Moore THM, Caldwell D, Davies P, Jones H, Furukawa TA, Lewis G, Hunot V (2012) ‘Cognitive behavioural therapies versus other psychological therapies for depression (Protocol)’ The Cochrane Collaboration.	

	for depression **(a full review on this subject has yet to be published)**<br><br>The protocol states that *'[d]espite the extensive evidence base demonstrating the effectiveness of CBT approaches, little attention has been given to any potential negative or adverse outcomes of therapy. Debate also continues over the potential mechanisms of action in CBT (Gaudiano 2008). For example, Ekers 2008 and colleagues undertook a systematic review of 17 RCTs and found CBT and behavioural therapy to be equivalent in terms of depression recovery rates, symptom levels and participant dropout*'.			

CBT & DEPRESSION: 27 years from first RCT to the NICE recommendation

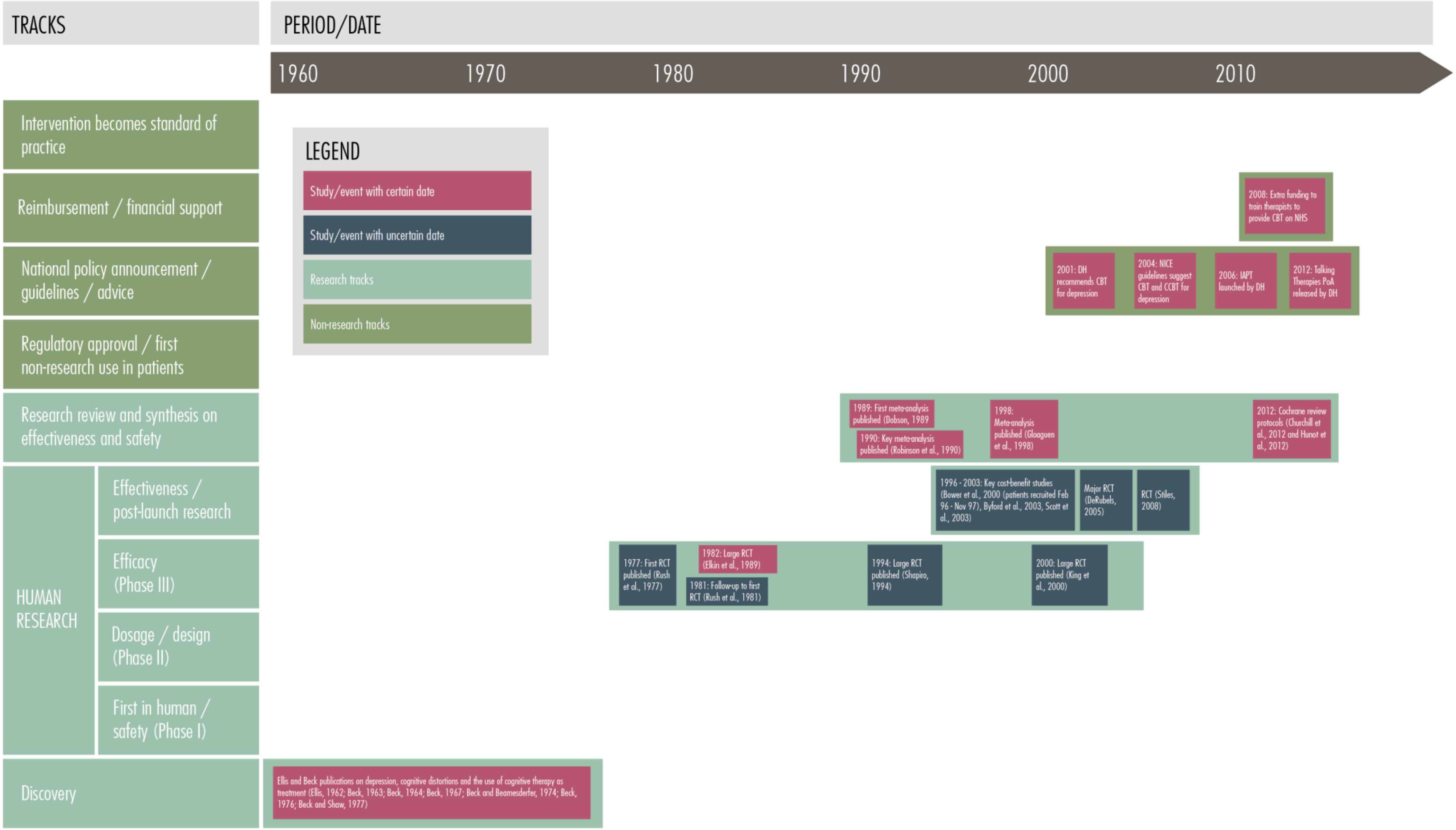

METHODOLOGICAL REFLECTIONS

This case study was developed through consulting a range of documents identified through literature searches in PubMed and Google Scholar, and analysing the references of each document. Therefore, the sole method employed in developing the case study was mining literature. This process was made more difficult given that a Cochrane review on the use of CBT and depression has not been published (although there are Cochrane reviews on the periphery of this subject). This may be something to bear in mind regarding recommendations to automate the process. It should be noted that this case study does not encompass all RCTs or studies more generally undertaken on the subject of CBT and depression (as there are a very large number). Rather, it attempts to identify key studies throughout the narrative. All RCTs undertaken after CBT was recommended to treat depression in NICE guidelines in 2004 have been categorised as 'effectiveness/post-launch research' along with cost-benefit studies which took place prior to 2004. Although RCTs are sure to continue beyond 2008, this stream stops based on the most recent relevant RCT in the Cochrane review protocol, which was the Stiles study published in 2008.

A key obstacle in developing the case study was identifying a range of calibration points such as proposal submitted, first patient recruited and dates of data collection. In most cases this information was not given in papers, and therefore where it was given it could not be used for reasons of consistency. If other case studies have used calibration points other than publication dates, the time lags between this work and others may not be comparable.

More generally, the matrix worked well in providing a visualisation of CBT's development. However, it did not provide any insight regarding the cause of time lags in CBT's development – particularly given that it does not capture negative events. If the matrix were to capture and clarify causes it would need to capture general narrative and context as well as just events – e.g. to show the lack of trained CBT practitioners in preventing the widespread adoption of CBT.

REFERENCES

Beck, A. T. Cognitive therapy and the emotional disorders. New York: International Universities Press, 1976.

Beck, A. T. Depression: Clinical experimental, and theoretical aspects. New York: Hoeber, 1967. (Republished as Depression: Causes and treatment. Philadelphia: University of Pennsylvania Press, 1972.)

Beck, A. T. Thinking and depression: I. Idiosyncratic content and cognitive distortions. Archives of General Psychiatry, 1963, 9, 324-333.

Beck, A. T. Thinking and depression: II. Theory and therapy. Archives of General Psychiatry, 1964, 10, 561-571.

Beck, A. T., & Beamesderfer, A. Assessment of depression: The depression inventory. In P. Pichot (Ed.), Modern problems in pharmacopsychiatry (Vol. 7). Basel, Switzerland: Karger, 1974.

Beck, A.T. (1979) Cognitive Therapy of Depression, Guilford Press, NY

Beck, A.T., & Shaw, B. F. Cognitive approaches to depression. In A. Ellis & R. Grieger (Eds.), Handbook of rational emotive theory and practice. New York: Springer, 1977.

Bower, P., Byford, S., Sibbald, B., et al. (2000) Randomised controlled trial of nondirective counselling, cognitive-behaviour therapy, and usual general practitioner care for patients with depression. II: Cost-effectiveness. British Medical Journal, 321, 1389–1392

Byford, S., Knapp, M., Greenshields, J., et al. (2003) Cost-effectiveness of brief cognitive behaviour therapy versus treatment as usual in recurrent deliberate self-harm: a decision-making approach. Psychological Medicine, 33, 977–986

Churchill R, Moore THM, Caldwell D, Davies P, Jones H, Furukawa TA, Lewis G, Hunot V (2012) 'Cognitive behavioural therapies versus other psychological therapies for depression (Protocol)' The Cochrane Collaboration.

Department of Health 'Treatment Choice in Psychological Therapies and Counselling'

DeRubeis, R. J., Hollon, S. D., Amsterdam, J. D., et al. (2005) Cognitive therapy versus medications in the treatment of moderate to severe depression. Archives of General Psychiatry, 62, 409–416

Dobson, K.S. (1989). A meta-analysis of the efficacy of cognitive therapy of depression. Journal of Consulting and Clinical Psychology, 57, 414-419.

Ekers, D., Richards, D., Gillbody, S. (2008) 'A meta-analysis of randomized trials of behavioural treatment of depression'. Psychological Medicine, 38(5): 611-23

Elkin, I., Shea, M.T., Watkins, J.T. et al. (1989) National Institute of Mental Health Treatment of Depression Collaborative Research Program. General effectiveness of treatments. Arch Gen Psychiatry. 1989 Nov; 46(11):971-82

Ellis, A. (1962) Reason and Emotion in Psychotherapy, Lyle Stewart: New York

Gloaguen, V., Cottraux, J. Cucherat, M. and Blackburn, I. (1998). A meta-analysis of the effects of cognitive therapy in depressed patients. Journal of Affective Disorders, 49, 59-72.

King M, Sibbald B, Ward E, Bower P, Lloyd M, Gabbay M, Byford S. (2000) Randomised controlled trial of non-directive counselling, cognitive-behaviour therapy and usual general practitioner care in the management of depression as well as mixed anxiety and depression in primary care. The National Coordinating Centre for Health Technology Assessment (NCCHTA) (Vol.4: No.19): 83.

Kovacs, M., Rush, A. T., Beck, A. T., et al. (1981) Depressed outpatients treated with cognitive therapy or pharmacotherapy: a one-year follow-up. Archives of General Psychiatry, 38, 33–39.

National Institute for Health and Clinical Excellence (2010) Depression: The treatment and Management of depression in adults (updated edition). National clinical Practice Guideline 90. The British Psychological Society and the Royal College of Psychiatrists

Robinson, L. A., Berman, J. S. & Neimeyer, R. A. (1990) Psychotherapy for the treatment of depression: a comprehensive review of controlled outcome research. Psychological Bulletin, 108, 30–49

Rush, A. J., Kovacs, M., Beck, A. T., et al. (1981) Differential effects of cognitive therapy and pharmacotherapy on depressive symptoms. Journal of Affective Disorders, 3, 221–229.

Rush, A.J., Beck, A.T., Hollon, S.D. et al., (1977) Comparative efficacy of cognitive therapy and pharmacotherapy in the treatment of depressed outpatients. Cogn. Ther. Res. 1 (1), 17-37.

Scott, J., Palmer, S., Paykel, E., et al. (2003) Use of cognitive therapy for relapse prevention in chronic depression. Cost-effectiveness study. British Journal of Psychiatry, 182, 221–227.

Shapiro, D.A., Barkham, M., Rees, A. et al. (1994) Effects of treatment duration and severity of depression on the effectiveness of cognitive-behavioural and psychodynamic-interpersonal psychotherapy. Journal of Consulting and Clinical Psychology, 62, 522-534.

Stiles, W.B., Barkham, M., Mellor-Clark, J. and Connell, J. (2008) 'Effectiveness of cognitive-behavioural, person-centred and psychodynamic therapies in UK primary-care routine practice: replication in a larger sample'. Psychological Medicine, 38. 677-688

Time lags between conducting medical research and its translation. Case Study 6: CBT and schizophrenia

NARRATIVE ACCOUNT OF BACKGROUND/ DEFINITIONS AND KEY ASPECTS OF THE INTERVENTION'S DEVELOPMENT

Definition of CBT

It is generally accepted that 'CBT' refers to a 'family of allied therapies that draw on a common base of behavioural and cognitive models of psychological disorders and utilise a set of overlapping techniques' (Churchill et al., 2012). The full NICE guidelines on depression define CBT as:

> *… time-limited, structured psychological interventions, derived from the cognitive behavioural model of affective disorders and where the patient:*
>
> - *works collaboratively with the therapist to identify the types and effects of thoughts, beliefs and interpretations on current symptoms, feelings states and/or problem areas*
> - *develops skills to identify, monitor and then counteract problematic thoughts, beliefs and interpretations related to the target symptoms/problems*
> - *learns a repertoire of coping skills appropriate to the target thoughts, beliefs and/or problem areas.*
>
> *In most individual trials of CBT, the manual used was Beck's Cognitive Therapy of Depression (1979) which advocates 16 to 20 sessions for treatment and relapse prevention work.*

The development of CBT to treat schizophrenia

A.T. Beck first applied CBT techniques to schizophrenia in 1952, although it had little impact as schizophrenia was thought to be incurable. Beck himself said that after publishing this paper he forgot about it and went on to focus on depression instead. This was reinforced by Freud's announcement in 1957 that psychoanalysis cannot be used as a treatment for schizophrenia as people with schizophrenia cannot form proper attachment to a therapist. From 1979 until the late 1980s very small studies were carried out (some only involving 1 patient), assessing how CBT could be applied to schizophrenic patients. This caused A.S. Bellack, president of the Association for the Advancement of Behavioural Therapy to describe schizophrenia as 'behaviour therapy's forgotten child' in 1986. (It is worthy of note that during this time studies for the use of CBT and depression were much larger and I have categorised them as phase III studies as opposed to phase II studies). However, throughout the 1980s there had been consistent findings that family environments were strongly influential in determining the recurrence of schizophrenic symptoms and a group of psychologists in the UK using rehabilitation approaches had also started taking cognitive approaches in understanding rehabilitation.

In 1988 David Kingdon was working on the Nottingham Neurosis Study (Tyrer et al, 1988, 1990) in which cognitive therapy was employed by a group of patients with neurotic disorders. Kingdon realised that the techniques he was using with patients who had psychoses could be described as a variant of cognitive therapy. This prompted him and colleagues to define the techniques used,

incorporate additional components, and refine them with further study of the literature. Throughout the early 1990s there were several developments of CBT interventions for psychotic patients and by 1996 there was enough evidence to start generating RCTs. From 1996-2002 there were 6 key RCTs with promising results. However, the first Cochrane review on the use of CBT to treat schizophrenia was published in 2002 and found that 'trial-based data supporting the wide use of cognitive behavioural therapy for people with schizophrenia or other psychotic illnesses are far from conclusive' (Cormac et al., 2002 p.2).

In 2002 the first NICE guidance was published which stated that CBT (and family interventions) should be used in the treatment of schizophrenia. This was followed in 2004 by the American Psychiatric Association recommending 'cognitive behaviourally oriented psychotherapy' to treat patients with schizophrenia, and in 2005 by the Canadian Psychiatric Association recommending CBT for treatment-resistant schizophrenic patients. The reason for accelerated approval in the UK may be because in the US the separation of psychology and psychiatry has prevented a multidisciplinary approach to treatment – which facilitated the development of CBT for the treatment of schizophrenia in the UK. Moreover, research funding in the US is more widely available in biological psychiatry which has contributed to the prominence of biological psychiatrists in the US.

In 2006 the National Forum for Assertive Outreach Annual Conference surveyed attendees to find out what the barriers were to the implementation of psychosocial interventions in their area. Respondents reported a lack of organisational investment, the structured nature of CBT, caseload issues, medication issues, application to people with sensory impairment, staff apathy and staff burnout as some of the barriers to the implementation of CBT for psychosis. In response to the lack of organisational investment, Improving Access to Psychological Therapies was launched by the Department for Health in the UK. *'From the outset it recognised that a national shortage of cognitive behavioural therapy (CBT) practitioners, who are skilled in helping people recover from depression and anxiety disorders, was the core deficiency preventing routine NHS delivery of the NICE guidelines' (Talking Therapies: A Four Year Plan of Action).* In 2008, Health Secretary Alan Johnson announced an extra 3,600 therapists will be trained to provide CBT treatment on the NHS at a cost of £173 million per year from 2010 as part of IAPT. Similarly in 2011 the Department of Health released 'Talking Therapies: A four-year plan of action' which aimed to develop talking therapies services that are recommended by NICE.

However, on 7 July 2008 leading psychotherapy experts claimed that the superiority of CBT is a myth at a conference at UEA. Similarly, in 2009 Practice Guidelines for the Treatment of Patients with Schizophrenia was published by the American Psychiatric Association which stated that there is not consistent evidence that CBT improves outcomes among individuals who are experiencing acute psychotic symptoms. In 2012 Jones et al. published a Cochrane review which found that '[t]rial-based evidence suggests no clear and convincing advantage for cognitive behavioural therapy over other - and sometime much less sophisticated - therapies for people with schizophrenia' (Jones et al., 2012). These findings may have come too late to have a real impact on the uptake of CBT, given the money that has already been invested into rolling out the treatment across the NHS.

TIMELINE

All dates in the period/date column refer to publication dates. Publication dates have also been used to draw the matrix.

Year	Event	Track	Calibration point	Comments
1952	Early paper by A.T. Beck applies CBT techniques in the treatment of a schizophrenic patient	Discovery	**Publications:**<br>Beck, A. T. (1952). Successful outpatient psychotherapy of a chronic schizophrenic with a delusion based on borrowed guilt. Psychiatry, 15, 305-312	A.T. Beck's paper had little impact as schizophrenia was thought to be incurable, even for those discharged from hospital, and rarely treated successfully in the community. Beck says himself that after publishing this paper, he forgot about it and went on to focus on depression instead.
1957	Freud announces that psychoanalysis cannot be used as a treatment for schizophrenia as people with schizophrenia cannot form a proper attachment to a therapist.	Narrative		
1962	Seminal study on rational emotive behaviour therapy (REBT) – an early form of CBT. (A. Ellis) (book)	Discovery	**Publications:**<br>Ellis, A. (1962) Reason and Emotion in Psychotherapy, Lyle Stewart: New York<br><br>Not cited in Cochrane reviews (v1, v2 or v3) or full NICE guidelines	
1979	A.T. Beck collaborates on a study with Richard Hole and	Human research – dosage/design	**Publications:**<br>Hole, R.W., Rush, A. J. & Beck, A.T. (1979) A cognitive investigation of	

	John Rush (Hole, Rush and Beck, 1979) in which cognitive interventions were applied in a more systematic way with the delusions of several patients who had schizophrenia – with promising results **N=8** (interviews)	(phase II)	schizophrenic delusions. Psychiatry, 42, 312-319.<br><br>Not cited in Cochrane reviews (v1, v2 or v3)	
1982	Study on the use of cognitive restructuring for the treatment of a chronic schizophrenic.<br>**N=1**	Human research – dosage/design (phase II)	**Publications:**<br>Alford, G. S., Fleece, L., & Rothblum, E. (1982). Hallucinatory-delusional verbalizations: Modification in a chronic schizophrenic by self-control and cognitive restructuring. Behavior Modification, 6, 421-435<br><br>Not cited in full NICE schizophrenia guidance or Cochrane reviews (v1, v2 or v3)	Cannot obtain other calibration points such as date of data collection etc.
1983	In Canada Hartman and Cashman publish a preliminary report on the use of cognitive-behavioural and psychopharmacological treatment of delusional symptoms **N=3**	Human research – dosage/design (phase II)	**Publications:**<br>Hartman, L. M., & Cashman, E E. (1983). Cognitive-behavioral and psychopharmacological treatment of delusional symptoms: A preliminary report. Behavioural Psychotherapy, H, 50-61.<br><br>**Reviews:**<br>Cited in Cochrane reviews (v1, v2 and v3) although it was excluded from the review because it is not an RCT.<br><br>Not cited in full NICE schizophrenia guidance	
1985	Throughout the 1980s, consistent findings that family environments, in terms of the measure of expressed emotion (EE) were strongly influential in determining the recurrence of schizophrenic symptoms promoted the development of family interventions that were successful in reducing relapse.	Narrative	**Publications:**<br>**First:** Goldstein, M. J., Rodnick, E. H., Evans, J. R., May, P. R. A. & Steinberg, M. R. (1978). Drug and family therapy in the aftercare of acute schizophrenics. Archives of General Psychiatry 35 1169-1177. **N=104**<br>**Guidelines:**<br>Cited in full NICE schizophrenia guidance<br><br>Not cited in Cochrane reviews (v1, v2 or v3)<br><br>**Publications:**<br>**Last:** Leff, J., Berkowitz, R., Shavit, N., Strachan, A., Glass, I. & Vaughn, C. (1989). A trial of family therapy versus a relative's group for schizophrenia.	Cannot obtain other calibration points such as date of data collection

			British Journal of Psychiatry 154, 58-66. **N=12**<br><br>**Guidelines:**<br>Cited in full NICE schizophrenia guidance<br><br>Not cited in Cochrane reviews (v1, v2 or v3)	
1985	Main proponents of CBT work started drawing on cognitive psychology and CBT as it was applied to psychosis and testing these approaches.	Narrative	N/A	
1985	Strong tradition of rehabilitation in UK meant that a group of psychologists who were working using rehabilitation approaches were also taking cognitive approaches and using cognitive understanding in their rehabilitation. This was in a number of centres in the UK, principally Manchester, Birmingham and London.	Narrative	N/A	
1986	Association for the Advancement of Behavioural Therapy (AABT) Presidential address by A.S. Bellack describes schizophrenia as 'behaviour therapy's forgotten child' and questions the erroneous assumptions about schizophrenia that prevented behaviour therapists from working on the disorder.	Narrative	N/A	
1988	P. Tyrer, et al. publish 'The Nottingham Study of Neurotic	Human research – efficacy (phase III)	**Publications:**<br>P. Tyrer, et al. (1988) 'The Nottingham Study of Neurotic Disorder Comparison of	

	Disorder Comparison of Drug and Psychological Treatments'. The Lancet 332:8605 (1988) 235-240.<br>**N=210**		Drug and Psychological Treatments'. The Lancet 332:8605 235-240<br><br>N.B. not cited in full schizophrenia guidance or Cochrane reviews (v1, v2 or v3)	
1988	David Kingdon was working on the Nottingham Neurosis Study (Tyrer et al, 1988, 1990) in which cognitive therapy was employed by a group of patients with neurotic disorders. Kingdon realised that the techniques he was using with patients who had psychoses could be described as a variant of cognitive therapy. This prompted him and colleagues to define the techniques used, incorporate additional components, and refine them with further study of the literature.	Narrative	**Publications:**<br>P. Tyrer, et al. (1988) 'The Nottingham Study of Neurotic Disorder Comparison of Drug and Psychological Treatments'. The Lancet 332:8605 235-240<br><br>Tyrer, P., Seivewright N., Ferguson, B., Murphy, S., Darling, C., Brothwell, J., Kingdon, D. and Johnson, A.L. (1990) 'The Nottingham Study of Neurotic Disorder: relationship between personality status and symptoms'. Psychological Medicine, Volume 20, Issue 02 May 1990 pp. 423-431<br><br>N.B. not cited in full schizophrenia guidance or Cochrane reviews (v1,v2 or v3)	
1990	Development of cognitive-behavioural interventions for psychotic patients as direct therapies for specific symptoms (Chadwick and Lowe, 1990) and as a way of enhancing patients' coping skills (e.g. Tarrier et al., 1990)	Human research – dosage/design (phase II)	**Publications:**<br>Chadwick, P. D. J. and Lowe, C. F. (1990) 'Measurement and modification of delusional beliefs', Journal of Consulting and Clinical Psychology, 58: 225-32. **N=6**<br>Not cited in Cochrane Reviews (v1, v2 or v3) or full NICE schizophrenia guidance<br><br>Tarrier, N., Harwood, S., Yusupoff, L., Beckett, R. and Baker, A. (1990) 'Coping strategy enhancement (CSE): A method of treating residual schizophrenic symptoms', Behavioural Psychotherapy 18: 283-93<br><br>Not cited in full NICE schizophrenia guidance or Cochrane Reviews (v1, v2 or v3)<br>**N=2**	Could not obtain other calibration points.
1990	Psychotherapy, in the form of Psychodynamic psychotherapy was largely discredited as a	Narrative		

	treatment for schizophrenia (Mueser & Berenbaum, 1990)			
1990	Vancouver conference. Seminal schizophrenia conference organised by the local mental health service in the university. Initially intended as a small meeting on developments in psychological treatments for psychosis, the event attracted a large crowd, demonstrating the growing interest in this subject. Attending this event were leading UK researchers in this area such as Douglas Turkington, David Kingdon, Paul Bebbington and Elizabeth Kuipers.	N/A		
1991	Development of cognitive-behaviour processes for psychotic patients as part of a normalising strategy designed to make patients more accepting of distressing experiences (e.g. Kingdon and Turkington, 1991). **N=64**	Human research – dosage/design (phase II)	**Publications:**<br>Kingdon, D.G. and Turkington, D. (1991) 'The use of cognitive behaviour therapy with a normalising rationale in schizophrenia', Journal of Nervous and Mental Disease 179: 207-211.<br><br>**Reviews:**<br>Cited in Cochrane reviews (v1, v2 and v3) although excluded from the review because it is not an RCT.<br><br>Not cited in full NICE guidance on schizophrenia	
1992	Chris Frith publishes The Cognitive Neuropsychology of Schizophrenia which introduces the idea that some symptoms of schizophrenia, such as thought-insertion and verbal auditory hallucinations,	N/A	**Publications:**<br>Frith, C.D. (1992) 'The Cognitive Neuropsychology of Schizophrenia (Essays in Cognitive Psychology)' Psychology Press.<br>**Theoretical.**<br><br>**Guidelines:**<br>Cited in full NICE guidance on schizophrenia	

	represent failures to self-monitor one's own thinking or conscious mental activity.		Not cited in Cochrane reviews (v1, v2 or v3)	
1992	N. Tarrier's 'Psychological treatment of positive schizophrenic symptoms' appears in: D. Kavanagh, ed. Schizophrenia: An Overview and Practical Handbook. London, UK: Chapman & Hall; 1992. This paper discusses an early form of CBT for schizophrenia which focuses on improving coping.	Cannot access books so unsure how to categorise	Publications:<br>Tarrier, N. 'Psychological treatment of positive schizophrenic symptoms'. In: Kavanagh D, ed. Schizophrenia: an overview and practical handbook. London: Chapman and Hall, 1992.<br><br>Not cited in full NICE guidance on schizophrenia or Cochrane reviews (v1, v2 or v3)	
1994	Further development of cognitive-behavioural interventions for psychotic patients as direct therapies for specific symptoms (e.g. Bentall et al., 1994; Chadwick and Birchwood, 1994; Garety et al., 1994)	Narrative		
1995	First International Conference on Psychological Treatments for Schizophrenia. 28-29 September 1995, Robinson College, Cambridge, England. This is a biannual event.	N/A	N/A	
1995	D. Fowler, P. Garety and E. Kuipers publish Cognitive-Behaviour Therapy for psychosis: Theory and Practice. Chichester, UK: Wiley; 1995.	Cannot access book so unsure how to categorise	**Publications:**<br>Fowler, D., Garety, P. and Kuipers, E. Cognitive-Behaviour Therapy for psychosis: Theory and Practice. Chichester, UK: Wiley; 1995.<br><br>Not cited in full NICE schizophrenia guidance or Cochrane reviews (v1, v2 or v3)	
1996	By this time there was enough evidence from the single case studies of the late 1980s and	Narrative	See rows below	

	early 1990s to start generating RCTs. These RCTs built up considerably throughout the mid-1990s.			
1996-2002	**6 key RCTs for the use of CBT on patients with schizophrenia**	Narrative	See rows below	
1996	RCT of cognitive behavioural therapy with acute inpatients Drury et al, 1996 **N=117**<br><br>Kemp et al, 1996 n=47, funded by the Medical Research Council	Human research – efficacy (phase III)	**Publications - Drury:**<br>Drury V, Birchwood M, Cochrane R, Macmillan F. Cognitive therapy and recovery from acute psychosis: A controlled trial. I. Impact on psychotic symptoms. British Journal of Psychiatry 1996; 169:593–601.<br><br>Drury V, Birchwood M, Cochrane R, Macmillan F. Cognitive therapy and recovery from acute psychosis: A controlled trial. II. Impact on recovery time. British Journal of Psychiatry 1996; 169:602–7.<br><br>**Guidelines:**<br>Cited in full NICE schizophrenia guidelines<br><br>**Reviews:**<br>Reviewed in Cochrane reviews (v1, v2 and v3)<br><br>**Publications - Kemp:**<br>Kemp, R., Hayward, P., Applewhaite, G. et al (1996) Compliance therapy in psychotic patients: randomised controlled trial. BMJ; 312:345<br><br>**Guidelines:**<br>Cited in full NICE schizophrenia guidelines<br><br>**Reviews:**<br>Reviewed in Cochrane Reviews (v1, v2 and v3)	Cannot obtain other calibration points
1997	Kuipers et al, 1997 **N=60**<br><br>Garety et al., 1997	Human research – efficacy (phase III)	**Publications:**<br>Kuipers, E., Garety, P., Fowler, D. et al. (1997) London-East Anglia randomised controlled trial of cognitive-behavioural therapy for psychosis. I: effects of the treatment phase. Br J Psychiatry; 171:319-27	

			Garety P, Fowler D, Kuipers E, Freeman D, Dunn G, Bebbington P, Jones S. London East-Anglia randomised controlled trial of cognitive behavioural therapy for psychosis: II. Predictors of outcome. British Journal of Psychiatry 1997; 171:420–6.<br><br>Kuipers E, Fowler D, Garety P, Chisholm D, Freeman D, Dunn G, Bebbington P, Hadley C. London East-Anglia randomised control trial of cognitive behavioural therapy or psychosis III: Follow-up and economic evaluation at 18 months. British Journal of Psychiatry 1998; 173:61–8.<br><br>**Reviews:**<br>Reviewed in Cochrane reviews (v1 and v2) although excluded from v3 because it reports on CBT compared to treatment as usual<br><br>**Guidelines:**<br>III cited in full NICE schizophrenia guidelines	
1998-2000 Cochrane reviews group as 1999	Tarrier et al, 1998 **N=87**, funded by the Wellcome Trust	Human research – efficacy (phase III)	**Publications:**<br>Tarrier N, Kinney C, McCarthy E, Humphreys L, Wittkowski A, Morris, J. Two-year follow-up of cognitive-behavioral therapy and supportive counselling in the treatment of persistent symptoms in chronic schizophrenia. Journal of Consulting and Clinical Psychology 2000;68(5): 917–22.<br>Tarrier N, Wittkowski A, Kinney C, McCarthy E, Morris J, Humpherys L. Durability of the effects of cognitive-behavioural therapy in the treatment of chronic schizophrenia: 12-month follow-up. British Journal of Psychiatry 1999;174:500–4.<br><br>Tarrier N, Yusupoff L, Kinney C, McCarthy E, Gledhill A, Haddock G, Morris J. Randomised controlled trial of intensive cognitive behavioural therapy for patients with chronic schizophrenia. BMJ 1998; 317:303–7.<br><br>**Reviews:**<br>Reviewed in Cochrane reviews (v1, v2 and v3)<br><br>**Guidelines:**	The Cochrane review also includes a study from 1993 in this group although I have removed from here (it was a controlled trial but not an RCT)

			Tarrier 1998 cited in full NICE schizophrenia guidance	
1998	Beck had begun to hear about the RCTs in the UK and starting the reading the literature. He invited the researchers to the US in order to find out more about what the therapy involved and how compatible it was his understanding of cognitive therapy. This was a significant meeting because it was the first time that UK researchers had formally met together. It also meant that Beck became a prominent 'champion' of CBT for schizophrenia in the US. At the instigation of Beck, this is now an annual meeting which is held either in Europe or North America to discuss progress.	Narrative		
2000	Sensky et al, 2000 **N=90**, funded by grant 039243, from the Wellcome Trust	Human research – efficacy (phase III)	**Publications:**<br>Sensky T, Turkington D, Kingdon D, Scott J, Siddle R, O-Carroll M, Barnes T. A randomized controlled trial of cognitive-behavioural therapy for persistent symptoms in schizophrenia resistant to medication. Archives of General Psychiatry 2000; 57:165–72.<br><br>**Reviews:**<br>Reviewed in Cochrane Reviews v1, v2 and v3<br><br>**Guidance:**<br>Cited in full NICE schizophrenia guidance	
2000	Drury et al, 2000 **N=34**, (cannot see funder information)	Human research – efficacy (phase III)	**Publications:**<br>Drury V, Birchwood M, Cochrane R. Cognitive therapy and recovery from acute psychosis: A controlled trial. British Journal of Psychiatry 2000; 177:8–14.	

			**Reviews:**<br>Reviewed in Cochrane Reviews v1, v2 and v3<br><br>Not cited in full NICE schizophrenia guidance, although Drury 1996 is which the Cochrane reviews group together with this study	
2002	'Schizophrenia: Core Interventions in the Treatment and Management of Schizophrenia in Primary and Secondary Care' was the first NICE guidance to be produced in 2002. It includes a recommendation that CBT (and family interventions) be used in the treatment of schizophrenia	National policy announcement/ guidelines/ advice	**Guidelines:**<br>NICE guidance – 'Schizophrenia: Core Interventions in the Treatment and Management of Schizophrenia in Primary and Secondary Care'	
2002	Turkington et al, 2002 **N=422**, funded by Pfizer	Human research – efficacy (phase III)	**Publications:**<br>Turkington D, Kingdon D, Turner T. Effectiveness of a brief cognitive behavioural therapy intervention in the treatment of schizophrenia. British Journal of Psychiatry 2002; 180:523–7.<br><br>**Reviews:**<br>Not cited in Cochrane review v1<br>Reviewed in Cochrane review v2<br>Cited in Cochrane Review v3 although excluded from the review as it compared CBT to treatment as usual rather than other psychosocial treatments<br><br>**Guidelines:**<br>Cited in full NICE schizophrenia guidelines	Does not state when data was collected or when funding was applied for. No other calibration points available.
2002	Meta-analyses of CBT	Research review and synthesis on effectiveness and safety	**Publications:**<br>Pilling, S. et al. (2002) Psychological treatments in schizophrenia: I. Meta-analysis of family intervention and cognitive behaviour therapy. Psychological Medicine, Volume 32, Issue 5, pp. 763-782<br><br>Pilling, S., Bebbington, P., Kuipers, E., et al. (2002) Psychological treatments in	

			schizophrenia: II. Meta-analyses of randomized controlled trials of social skills training and cognitive remediation. Psychological Medicine, 32, 783–791<br><br>**Reviews:**<br>II cited in Cochrane review v3<br><br>**Guidelines:**<br>II cited in full schizophrenia guidance	
2002	Cormac et al. publish Cochrane review on the use of CBT for the treatment of schizophrenia	Research review and synthesis on effectiveness and safety	**Publications:**<br>Cormac I, Jones C, Campbell C, Silveira DMNJ. Cognitive behaviour therapy for schizophrenia. The Cochrane Database of Systematic Reviews 2002, Issue 1. Art. No.: CD000524. DOI: 10.1002/14651858.CD000524.	
2004	American Psychiatric Association Practice guidelines for the treatment of patients with schizophrenia includes 'cognitive behaviourally oriented psychotherapy' among the recommended treatments for patients	National policy announcement/ guidelines/ advice	**Guidelines:**<br>Lehman, A., Lieberman, J.A., Dixon, L.B., McGlashan, T.H., Miller, A.L., Perkins, D.O., Kreyenbuhl, J. 'Practice Guideline for the Treatment of Patients With Schizophrenia: Second Edition' February 2004.	
2004	New studies support for role of CBT in reducing both positive and negative symptoms of schizophrenia (Trower et al, 2004) and improving social functioning (M. Startup, M.C. Jackson, S. Bendix, 2004).	Human research – effectiveness/ post-launch	**Publications:**<br>Trower P, Birchwood M, Meaden A, Byrne S, Nelson A, Ross K. Cognitive therapy for command hallucinations: randomised controlled trial.<br>Br J Psychiatry. 2004 Apr;184:312-20<br><br>**Reviews:**<br>Cited in Cochrane review v3 as background material (although not reviewed)<br><br>**Guidelines:**<br>Cited in full NICE schizophrenia guidelines<br><br>**Publications:**<br>Startup M, Jackson MC, Bendix S. North Wales randomized controlled trial of cognitive behaviour therapy for acute schizophrenia spectrum disorders: outcomes at 6 and 12 months. Psychol Med. 2004 Apr;34(3):413-22.	

			**Guidelines:**<br>Cited in full NICE schizophrenia guidelines<br><br>Not cited in Cochrane reviews v1, v2 or v3	
2004	Jones et al. produce a Cochrane Review	Research review & synthesis on effectiveness and safety	**Cochrane Review:**<br>Publication of Cochrane Review (updated in 2012)<br><br>Not cited in full NICE schizophrenia guidelines	
2005	Canadian Psychiatric Association publishes practical guidelines recommending CBT be offered to treatment-resistant patients	National policy announcement/ guidelines/ advice	**Guidelines:**<br>Canadian Journal of Psychiatry, Clinical Practice Guidelines: Treatment of Schizophrenia November 2005, (Vol 50, Supplement 1)	
2006	National Forum for Assertive Outreach Annual Conference surveyed attendees to find out what the barriers were to the implementation of psychosocial interventions in their area. Respondents reported a lack of organisational investment, the structured nature of CBT, caseload issues, medication issues, application to people with sensory impairment, staff apathy and staff burnout as some of the barriers to the implementation of CBT for psychosis.	Narrative	N/A	
2006	Improving Access to Psychological Therapies launched by Department for Health with a focus on adults of working age (although in 2010 it was broadened to	National policy announcement/ guidelines/ advice	N/A	

	adults of all ages)<br><br>*'From the outset it recognised that a national shortage of cognitive behavioural therapy (CBT) practitioners, who are skilled in helping people recover from depression and anxiety disorders, was the core deficiency preventing routine NHS delivery of the NICE guidelines'*			
2008	Health Secretary Alan Johnson announces an extra 3,600 therapists will be trained to provide CBT treatment on the NHS at a cost of £173 million per year from 2010 (as part of IAPT).	Reimbursement/ financial support	N/A	
2008	Further support for role of CBT in reducing both positive and negative symptoms of schizophrenia (D. Turkington, D. Kingdon P.J. Weiden, et al, 2006; 2008).	Human research – effectiveness/ post-launch research	**Publications:**<br>Turkington, D., Kingdon, D. and Weiden, P.J. (2006) Cognitive behavior therapy for schizophrenia. Am J Psychiatry. 2006 Mar;163(3):365-73<br><br>Turkington, D., Kingdon, D. and Weiden, P.J. (2008) Cognitive behavior therapy for schizophrenia. FOCUS 2008; 6:257-266<br><br>Not cited in full NICE schizophrenia guidelines, or Cochrane reviews	N.B. other Turkington 2006 and 2008 papers are cited in Cochrane review v3
2008	Leading psychotherapy experts claim the superiority of CBT is a myth at a conference at UEA on 7 July 2008.	Narrative	N/A	
2009	Support for role of CBT for reducing the occurrence of violence on schizophrenic patients (Haddock et al., 2009)	Human research – effectiveness/ post-launch research	**Publications:**<br>Haddock G, Barrowclough C, Shaw JJ, Dunn G, Novaco RW, Tarrier N. (2009) Cognitive-behavioural therapy v. social activity therapy for people with psychosis and a history of violence: randomised controlled trial. British Journal of Psychiatry;194(2):152–7.	

			**Cochrane review:**<br>Cited in Cochrane review v3<br><br>Not cited in full NICE schizophrenia guidelines.	
2009	Practice Guideline for the Treatment of Patients with Schizophrenia published by the American Psychiatric Association states that there is not consistent evidence that CBT improves outcomes among individuals who are experiencing acute psychotic symptoms	National policy announcement/ guidelines/ advice	**Guidelines:**<br>Dixon, L., Perkins, D. and Calmes, C. Guideline Watch (September 2009): Practice Guideline for the Treatment of Patients With Schizophrenia http://psychiatryonline.org/content.aspx?bookid=28&sectionid=1682213	
2009	Klingberg et al. assess CBT for treating the negative symptoms of schizophrenia and find that CBT for the reduction of negative symptoms is feasible and can be conducted safely	Human research – effectiveness/ post-launch research	**Publications:**<br>Klingberg S, Wittorf A, Herrlich J, Wiedemann G, Meisner C, Buchkremer G, Frommann N, Wölwer W. Cognitive behavioural treatment of negative symptoms in schizophrenia patients: study design of the TONES study, feasibility and safety of treatment. European Archives of Psychiatry and Clinical Neuroscience 2009;259(Suppl 2): 149-54.<br><br>**Cochrane review:**<br>Cited in Cochrane review v3<br><br>Not cited in full NICE schizophrenia guidelines.	

2009	Penn et al. assess CBT vs. supportive therapy for auditory hallucinations and find that supportive therapy as a specific impact on auditory hallucinations whereas CBT impacts on general psychotic symptoms.	Human research – effectiveness/ post-launch research	**Publications:**<br>Penn DL, Meyer PS, Evans E, Wirth RJ, Cai K, Burchinal M. A randomized controlled trial of group cognitive behavioral therapy vs. enhanced supportive therapy for auditory hallucinations. Schizophrenia Research 2009;109: 52–9.<br><br>**Cochrane review:**<br>Cited in Cochrane review v3<br><br>Not cited in full NICE schizophrenia guidelines.	
2009	Updated NICE guidelines on schizophrenia published, advising that CBT should be offered to 'all people with schizophrenia'	National policy announcement/ guidelines/ advice	**Guidelines:**<br>NICE Clinical guidelines for schizophrenia CG 82<br>http://www.nice.org.uk/nicemedia/live/11786/43608/43608.pdf	
2011	'Talking Therapies: A four-year plan of action' released by the Department of Health.<br><br>'The aim is to develop talking therapies services that offer treatment for depression and anxiety disorders approved by the National Institute for Health and Clinical Excellence (NICE) across England by March 2015, the end of the Spending Review period'.	National policy announcement/ guidelines/ advice	N/A	

	The plan states that 'the evidence that proved CBT is as effective as medication in helping people with depression and anxiety disorders – and better at preventing relapse – led to the economic case that secured annual funding to begin the national roll-out in the three years to March 2011. Key to the economic case was an argument that effective therapeutic interventions combined with employment support could reduce the numbers of people on sick pay and benefits'.			

CBT & SCHIZOPHRENIA: 12 years between development of CBT as direct therapy for psychosis and NICE recommendation

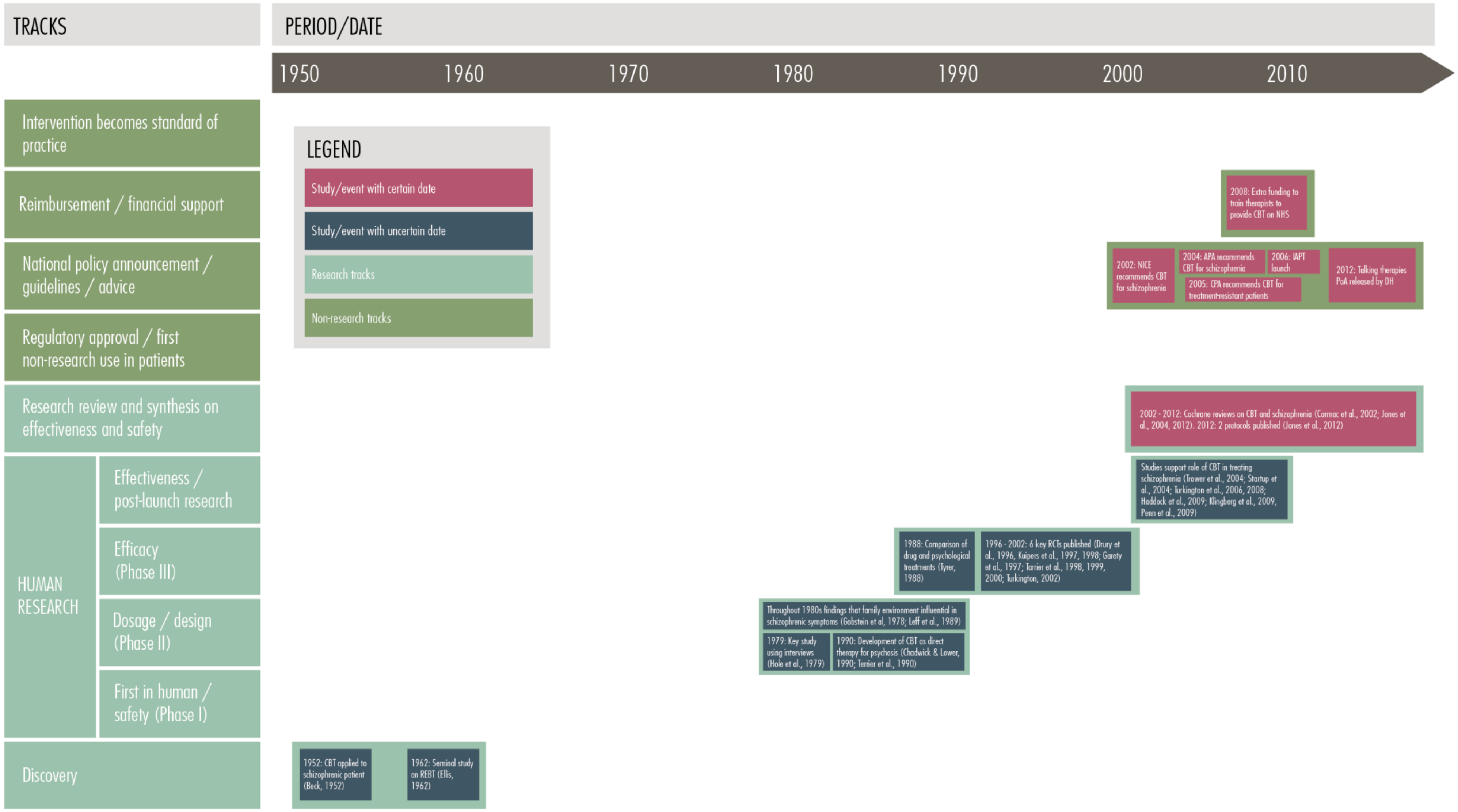

METHODOLOGICAL REFLECTIONS

This case study was developed through drawing upon pre-existing timelines developed by RAND Europe which set out the development of the use of CBT to treat schizophrenia. This was then cross-referenced and supplemented with exploring three versions of Cochrane reviews. Therefore, the primary method in developing the case study was mining literature. It should be noted that this case study does not encompass all RCTs or studies undertaken in regard to CBT and schizophrenia. Rather, it attempts to identify key studies in the narrative. All RCTs undertaken after 2002 when CBT was recommended to treat schizophrenia in NICE guidelines are categorised as 'effectiveness/post launch research'. Although RCTs are sure to continue beyond 2009, this stream stops based on the most recent relevant 2009 RCTs reviewed in the 2012 Cochrane review.

A key obstacle in developing the case study was identifying a range of calibration points such as proposal submitted, first patient recruited and dates of data collection. In most cases this information was not given in papers, and therefore where it was given it could not be used for reasons of consistency. If other case studies have used calibration points other than publication dates, the time lags between this work and others may not be comparable.

More generally, the matrix worked well in providing a visualisation of CBT's development. However, it did not provide any insight regarding the cause of time lags in CBT's development – particularly given that it does not capture negative events. If the matrix were to capture and clarify causes it would need to capture general narrative and context as well as just events – e.g. to show the lack of trained CBT practitioners in preventing the widespread adoption of CBT.

REFERENCES

Alford, G. S., Fleece, L., & Rothblum, E. (1982). Hallucinatory-delusional verbalizations: Modification in a chronic schizophrenic by self-control and cognitive restructuring. Behavior Modification, 6, 421-435

Beck, A. T. (1952). Successful outpatient psychotherapy of a chronic schizophrenic with a delusion based on borrowed guilt. Psychiatry, 15, 305-312

Chadwick, P. D. J. and Lowe, C. F. (1990) 'Measurement and modification of delusional beliefs', Journal of Consulting and Clinical Psychology, 58: 225-32.

Cormac I, Jones C, Campbell C, Silveira DMNJ. Cognitive behaviour therapy for schizophrenia. The Cochrane Database of Systematic Reviews 2002, Issue 1. Art. No.: CD000524. DOI: 10.1002/14651858.CD000524.(v1)

Drury V, Birchwood M, Cochrane R, Macmillan F. (1996). Cognitive therapy and recovery from acute psychosis: A controlled trial. I. Impact on psychotic symptoms. British Journal of Psychiatry; 169:593–601.

Drury V, Birchwood M, Cochrane R, Macmillan F. (1996) Cognitive therapy and recovery from acute psychosis: A controlled trial. II. Impact on recovery time. British Journal of Psychiatry; 169:602–7.

Drury V, Birchwood M, Cochrane R. (2000). Cognitive therapy and recovery from acute psychosis: A controlled trial. British Journal of Psychiatry; 177:8–14.

Ellis, A. (1962) Reason and Emotion in Psychotherapy, Lyle Stewart: New York.

Fowler, D., Garety, P. and Kuipers, E. Cognitive-Behaviour Therapy for psychosis: Theory and Practice. Chichester, UK: Wiley; 1995.

Frith, C.D. (1992) 'The Cognitive Neuropsychology of Schizophrenia (Essays in Cognitive Psychology)' Psychology Press.

Garety P, Fowler D, Kuipers E, Freeman D, Dunn G, Bebbington P, Jones S. (1997) London East-Anglia randomised controlled trial of cognitive behavioural therapy for psychosis: II. Predictors of outcome. British Journal of Psychiatry; 171:420–6.

Goldstein, M. J., Rodnick, E. H., Evans, J. R., May, P. R. A. & Hartman, L. M., & Cashman, E E. (1983). Cognitive-behavioral and psychopharmacological treatment of delusional symptoms: A preliminary report. Behavioural Psychotherapy, H, 50-61.

Hole, R.W., Rush, A. J. & Beck, A.T. (1979) A cognitive investigation of schizophrenic delusions. Psychiatry, 42, 312-319.

Jones C, Campbell C, Cormac I, Hacker D, Meaden A. (2009). Cognitive behaviour therapy versus standard care for schizophrenia (Protocol). Cochrane Database of Systematic Reviews, Issue 3. Art. No.: CD007964. DOI: 10.1002/14651858.CD007964.

Jones C, Cormac I, Campbell C, Meaden A, Hacker D. Cognitive behaviour therapy versus specific pharmacological treatments for schizophrenia (Protocol) (2009). Cochrane Database of Systematic Reviews, Issue 3. Art. No.: CD007965. DOI: 10.1002/14651858.CD007965.

Jones C, Cormac I, Silveira da Mota Neto JI, Campbell C. (2004). Cognitive behaviour therapy for schizophrenia. Cochrane Database of Systematic Reviews, Issue 4. Art. No.: CD000524. DOI: 10.1002/14651858.CD000524.pub2 (v2).

Jones C, Hacker D, Cormac I, Meaden A, Irving CB. Cognitive behaviour therapy versus other psychosocial treatments for schizophrenia. Cochrane Database of Systematic Reviews 2012, Issue 4. Art. No.: CD008712. DOI: 10.1002/14651858.CD008712.pub2 (v3).

Kemp, R., Hayward, P., Applewhaite, G. et al (1996) Compliance therapy in psychotic patients: randomised controlled trial. BMJ; 312:345.

Kingdon, D.G. and Turkington, D. (1991) 'The use of cognitive behaviour therapy with a normalising rationale in schizophrenia', Journal of Nervous and Mental Disease 179: 207-211.

Kuipers E, Fowler D, Garety P, Chisholm D, Freeman D, Dunn G, Bebbington P, Hadley C. London East-Anglia randomised control trial of cognitive behavioural therapy or psychosis III: Follow-up and economic evaluation at 18 months. British Journal of Psychiatry 1998; 173:61–8.

Kuipers, E., Garety, P., Fowler, D. et al. (1997) London-East Anglia randomised controlled trial of cognitive-behavioural therapy for psychosis. I: effects of the treatment phase. Br J Psychiatry; 171:319-27.

Leff, J., Berkowitz, R., Shavit, N., Strachan, A., Glass, I. & Vaughn, C. (1989). A trial of family therapy versus a relative's group for schizophrenia. British Journal of Psychiatry 154, 58-66.

Lehman, A., Lieberman, J.A., Dixon, L.B., McGlashan, T.H., Miller, A.L., Perkins, D.O., Kreyenbuhl, J. 'Practice Guideline for the Treatment of Patients With Schizophrenia: Second Edition' February 2004.

P. Tyrer, et al. (1988) 'The Nottingham Study of Neurotic Disorder Comparison of Drug and Psychological Treatments'. The Lancet 332:8605 235-240.

Pilling, S. et al. (2002) Psychological treatments in schizophrenia: I. Meta-analysis of family intervention and cognitive behaviour therapy. Psychological Medicine, Volume 32, Issue 5, pp. 763-782.

Pilling, S., Bebbington, P., Kuipers, E., et al. (2002) Psychological treatments in schizophrenia: II. Meta-analyses of randomized controlled trials of social skills training and cognitive remediation. Psychological Medicine, 32, 783–791.

Sensky T, Turkington D, Kingdon D, Scott J, Siddle R, O-Carroll M, Barnes T. A randomized controlled trial of cognitive-behavioural therapy for persistent symptoms in schizophrenia resistant to medication. Archives of General Psychiatry 2000; 57:165–72.

Startup M, Jackson MC, Bendix S. North Wales randomized controlled trial of cognitive behaviour therapy for acute schizophrenia spectrum disorders: outcomes at 6 and 12 months. Psychol Med. 2004 Apr;34(3):413-22.

Steinberg, M. R. (1978). Drug and family therapy in the aftercare of acute schizophrenics. Archives of General Psychiatry 35 1169-1177.

Tarrier N, Kinney C, McCarthy E, Humphreys L, Wittkowski A, Morris, J. Two-year follow-up of cognitive-behavioral therapy and supportive counselling in the treatment of persistent symptoms in chronic schizophrenia. Journal of Consulting and Clinical Psychology 2000;68(5): 917–22.

Tarrier N, Wittkowski A, Kinney C, McCarthy E, Morris J, Humpherys L. Durability of the effects of cognitive-behavioural therapy in the treatment of chronic schizophrenia: 12-month follow-up. British Journal of Psychiatry 1999;174:500–4.

Tarrier N, Yusupoff L, Kinney C, McCarthy E, Gledhill A, Haddock G, Morris J. Randomised controlled trial of intensive cognitive behavioural therapy for patients with chronic schizophrenia. BMJ 1998; 317:303–7.

Tarrier, N. 'Psychological treatment of positive schizophrenic symptoms'. In: Kavanagh D, ed. Schizophrenia: an overview and practical handbook. London: Chapman and Hall, 1992.

Tarrier, N., Harwood, S., Yusupoff, L., Beckett, R. and Baker, A. (1990) 'Coping strategy enhancement (CSE): A method of treating residual schizophrenic symptoms', Behavioural Psychotherapy 18: 283-93.

Trower P, Birchwood M, Meaden A, Byrne S, Nelson A, Ross K. Cognitive therapy for command hallucinations: randomised controlled trial. Br J Psychiatry. 2004 Apr;184:312-20.

Turkington D, Kingdon D, Turner T. Effectiveness of a brief cognitive behavioural therapy intervention in the treatment of schizophrenia. British Journal of Psychiatry 2002; 180:523–7.

Turkington, D., Kingdon, D. and Weiden, P.J. (2006) Cognitive behavior therapy for schizophrenia. Am J Psychiatry. 2006 Mar;163(3):365-73.

Turkington, D., Kingdon, D. and Weiden, P.J. (2008) Cognitive behavior therapy for schizophrenia. FOCUS 2008; 6:257-266.

Tyrer, P., Seivewright N., Ferguson, B., Murphy, S., Darling, C., Brothwell, J., Kingdon, D. and Johnson, A.L. (1990) 'The Nottingham Study of Neurotic Disorder: relationship between personality status and symptoms'. Psychological Medicine, Volume 20, Issue 02 May 1990 pp. 423-431.

Time lags between conducting medical research and its translation. Case Study 7: Early intervention in schizophrenia

NARRATIVE ACCOUNT OF BACKGROUND/ DEFINITIONS AND KEY ASPECTS OF THE INTERVENTION'S DEVELOPMENT

Early intervention in schizophrenia may refer to intervention in either the prodromal stage (i.e. to prevent onset of a psychotic episode) or the first episode stage (i.e. focusing on prompt detection and treatment of psychosis). Some studies have associated tailored first-episode interventions with an improvement in treatment response and long-term outcomes, and at the very least these engage people with care at an early stage in order to reduce suffering, but there have been few randomised controlled trials demonstrating a causal link. In the last ten years there have been efforts to reach individuals with early intervention and treatment in the prodromal stage. Though this is still an emerging area, there are some studies, including five randomised controlled clinical trials, which have demonstrated the potential effectiveness of prodromal interventions. Nonetheless, many argue that the evidence base is not considered strong and there remains debate in the literature and within the schizophrenia research community about the nature and robustness of the evidence base around the effectiveness of both prodromal interventions and first-episode interventions.

In the absence of national research and development strategies during the initial stages of development, many of the key events and initiatives in the development of early intervention services were driven at a local level – for example, the work of Patrick McGorry and Alison Yung at the EPPIC centre and PACE clinic in Australia. These intervention programmes and services for both the prodromal and first-episode stages not only established an initial evidence base, but also brought many researchers together to form an international network which began to establish similar programmes and services in other localities.

Despite locally strong initiatives in different countries, comparison between Canada, UK and USA suggests only the UK has evidence of a national research and clinical delivery strategy for intervention in the first-episode stage. There has also been considerable policy and advocacy activity at a national level, although this has triggered extensive debate, particularly around prodromal intervention. The suggestion that an ultra-high-risk group can be identified and treated based on prodromal symptoms is controversial, due to the possibility of 'false positive' diagnoses and unnecessary stigmatisation and discrimination. While a 'risk syndrome' was initially proposed for inclusion in DSM-V, this was subsequently dropped.

Due to the lack of clarity over the strength of the evidence base for the effectiveness of either form of early intervention, the practice guidelines in the USA, the UK and Canada vary in the degree to which they promote early intervention. Although the guidelines generally recognise the importance of providing treatment as early as possible and recommend intervention at the first-episode stage, they do not identify a specific tailored approach for this intervention, and none recommends prodromal interventions.

TIMELINE

Time	Event/Study	Track	Calibration point
1979-1990	Northwick Park Study of early intervention in the first episode stage (England)<br>*Highlighted treatment delay in schizophrenia was important in determining outcomes ie "the most important determinant of relapse was duration of illness prior to starting neuroleptic medication" Crow et al 1986*	**Human research**	**Publications**: Johnston et al 1986; Crow et al 1986<br><br>**<u>Cited</u> on Systematic Review v1, v2 & v3 (2011) [Cochrane Collaboration, Marshall and Rathbone]:** Crow et al 1986 but excluded from systematic review as a drug study.
1984-1988	Buckingham study of early intervention in the prodromal stage (England)<br><br>*The first study to focus on early detection of psychosis in the prodromal period. Considered one of the first early intervention programs. Aim was to integrate mental health care into the primary care system in a rural English community*.	**Human research**	**Publications**: Falloon 1992; Falloon et al 1996; Falloon 2000<br><br>**<u>Cited</u> on Systematic Review v1, v2 & v3 (2011) [Cochrane Collaboration, Marshall and Rathbone]:** Falloon 1992 and 2000 but excluded from systematic review as non-randomised.
1997-	Cologne Early Recognition and Intervention Centre (FETZ)<br><br>*The Cologne Early Recognition and Intervention Center for mental crises (FETZ) was set up in late 1997 as the first European center dedicated to the early detection of psychosis prior to first episode. Following the German psychopathologic tradition, basic symptoms, especially cognitive-perceptive disturbances, are employed as a means to this aim in addition to the internationally established "ultra-high risk" (UHR) criteria.*	**Human research**	
1992	Early Psychosis Prevention and Intervention Centre (EPPIC) program for early intervention in the first episode stage established (Australia)<br>*NB – a number of subsequent RCTs came out of this centre (PACE-Australia, Berger-Australia; Edwards-Australia; Jackson-Australia, LifeSPAN-Australia; Killackey-Australia – ie 6/18 trails identified in the Cochrane review*)	**Human research**	
1992-	Linszen – Amsterdam<br><br>*Recruited participants aged from15 to 26 who were experiencing their first episode of schizophrenia and living in close contact with parents or relatives. All participants were recruited from an adolescent clinic and had to agree to an initial three months' inpatient programme before randomisation. Subsequent treatment took place on an outpatient basis.*	**Efficacy (Phase III)**	**Publications:** Linszen et al 2001; Linszen et al 1996; Linszen et al 1998; Linszen et al 1998; Linsezen et al 1998; Lenior et al 2003; Lenior et al 2005; Lenior et al 2005; Linszen et al 2004; Linsezen eta l 2004; Linszen eta l 2003<br><br>**<u>Cited</u> on Systematic Review v2 a& v3 (2011) [Cochrane Collaboration, Marshall**

			**and Rathbone]** as Linszen – Amsterdam:<br>V1. Linszen et al 2001; Linszen et al 1996; Linszen et al 1998; Linszen et al 1998<br>V2: Linszen et al 1998<br>V3: Lenior et al 2003; Lenior et al 2005; Lenior et al 2005; Linszen et al 2004; Linsezen eta l 2004; Linszen eta l 2003<br><br>**<u>Cited</u> on Clinical Guideline (2010) [NICE full guideline published by BPS and RCP]**: Study ID is LINSZEN1996. Paper cited is Linszen et al 1996
1992-1999	Sør-Trøndelag County, Norway<br><br>*One centre in the International Optimal Treatment multi-site project that aims to evaluate the effects of continuous implementation of evidence-based integrated biomedical and psychosocial interventions in routine services for patients with recent-onset and chronic schizophrenia*	**Human research**	**Publication**: Grawe et al 2006; Morken et al 2007<br><br>**<u>Cited</u> on Clinical Guideline (2010) [NICE full guideline published by BPS and RCP]**: Study ID is GRAWE2006-OTP. Papers cited are Grawe et al 2006; Morken et al 2007.
1994	Personal Assessment and Crisis Evaluation Service (PACE-Australia) for early intervention in the prodromal stage established.<br><br>*Recruited participants referred to the Personal Assessment and Crisis Evaluation clinic, which is part of the EPPIC programme. Participants were from 14 to 30 years of age, and met Yung criteria for an 'ultra high risk' mental state. The intervention involved prescription of low dose risperidone (1-2 mg/day) combined with modified CBT, which aimed to enhance understanding and control of symptoms. Both the intervention and control groups also received case management from a PACE therapist.*	**Human Research**	**Publications**: McGorry et al 2002; Phillips et al 2002; Phillips et al 2007<br><br>**<u>Cited</u> on Systematic Review v1, v2 & v3 (2011) [Cochrane Collaboration, Marshall and Rathbone]** as PACE-Australia:<br>V1. : McGorry et al 2002<br>V2: McGorry et al 2002; Phillips et al 2002a; Phillips et al 2002b<br>V3: : McGorry et al 2002; Phillips et al 2002a; Phillips et al 2002b; Phillips et al 2007<br>*[NB not cited on NICE guidelines]*
1995-	Birmingham Early Intervention Service for young people in the first episode stage established (1995) leading to REDIRECT study from 2004 onwards<br><br>*The Redirect trial was conducted in three Primary Care Trusts (PCTs) within Birmingham in the UK. Eighty-nine general practices were eligible for inclusion to the trial The Redirect study team designed an evidence based "complex" educational intervention that addressed the knowledge, skills, and attitudes of GPs about First Episode Psychosis (FEP,, given that most GPs see only one or two new people with FEP each year. The intervention was tested in a stratified cluster randomised controlled trial to evaluate the effect of the educational intervention on GP referral rates of young people with FEP to EIS.*	**Human research**	**Publications**: Tait et al 2005, Lester et al 2006<br>**<u>Cited</u> on Systematic Review v2 a& v3 (2011) [Cochrane Collaboration, Marshall and Rathbone]**.<br>V1: No citation<br>V2: Tait et al 2005 but excluded ("no useable data")<br>V3: Lester et al 2006, excluding as on-going study ("References to studies awaiting assessment")

			[NB not cited on full NICE guidelines]
1996	First international early intervention conference held in Melbourne (Australia)		[n/a]
1997-1999<br><br>*[NB this is the duration that the LifeSPAN program operated]*	LifeSPAN-Australia.<br><br>*Recruited participants from the Western region of Melbourne, Australia and is part of the EPPIC programme, which includes an early detection and crisis assessment team. Participants were aged 15 to 29 years, and were acutely suicidal. The intervention group received standard clinical care plus LifeSPAN therapy which draws on the experience at EPPIC with Cognitive Orientated Therapy for Early Psychosis (COPE) and suicide manuals such as Choosing to Live and Cognitive Therapy of Suicide Behaviour. Four phases are used for the intervention: (a) initial engagement, (b) suicide risk assessment/ formulation, (c) cognitive modules and (d) final closure/handover*.	**Efficacy (Phase III)**	**Publication:** Powers et al 2004<br><br>**<u>Cited</u> on Systematic Review v2 a& v3 (2011) [Cochrane Collaboration, Marshall and Rathbone]** as LifeSPAN-Australia:<br><br>V1. No citation or reference<br><br>V2: Powers et al 2004<br><br>V3: Powers et al 2004<br><br>*[NB not cited on full NICE guidelines]*
1997-2003	PRIME early intervention study during the prodromal stage (PRIME-USA)<br><br>*Participants were aged from 12 to 36 years with a diagnosis of being at risk of developing psychosis. Participants were recruited at four sites (three in the USA and one in Canada). The trial randomised participants to olanzapine 5-15mg/day (mean 8mg/day) or placebo for one year, and then followed up for a further year without medication. Individual and family psychosocial interventions with supportive and psychoeducational components were available to all patients during the first year.*	**Efficacy (Phase III)**	**Publications**: McGlashan et l 2006; Hawkins et al 2008; Hoffman et al 2007; McGlashan et al 2003<br><br>**<u>Cited</u> on Systematic Review v2 a& v3 (2011) [Cochrane Collaboration, Marshall and Rathbone]** as PRIME-USA:<br><br>V1. Conference paper (not listed above) cited as on-going study<br><br>V2: McGlashan et al 2006; McGlashan et al 2003a;<br><br>V3: McGlashan et l 2006; Hawkins et al 2008; Hoffman et al 2007; McGlashan et al 2003<br><br>*[NB not cited on full NICE guidelines]*
1997-2000	Treatment and Intervention in Psychosis Study (TIPS) (Norway)	**Efficacy (Phase III)**	**Publications**: Melle et al 2005; Melle et al 2006<br><br>**<u>Cited</u> on Systematic Review v1, v2 & v3 (2011) [Cochrane Collaboration, Marshall and Rathbone]**:<br><br>V1: Series of conference papers cited as on-going study<br><br>V2: No citations<br><br>V3 Melle et al 2005; Melle et al 2006 but excluded from study as not randomised.

			[NB not cited on full NICE guidelines]
1997	Follow-up early intervention conference held in Stratford-upon-Avon (England)		[n/a]
1997	International Early Psychosis Association (IEPA) founded (Australia)		[n/a]
1998-<br><br>[Recruitment to the study was between March 1998 and December 1999]	RCT of cannabis focused intervention for young people with first episode psychosis. (Edwards-Australia)<br><br>*Included participants with first-episode psychosis diagnosed as having a psychotic disorder using DSM-IV criteria. The study was undertaken at the EPPIC centre in Australia. The intervention group received a behavioural modification intervention, Cannabis and Psychosis Therapy (CAP). This consisted of weekly sessions of CBT provided by trained clinicians over three months*.	**Efficacy (Phase III)**	**Publication:** Edwards et al 2006<br><br>**Cited on Systematic Review v1, v2 & v3 (2011) [Cochrane Collaboration, Marshall and Rathbone]** as Edwards-Australia.<br>V1: No mention<br>V2: No mention<br>V3: Edwards et al 2006<br><br>*[NB not cited on full NICE guidelines]*
1998-<br><br>[Recruitment to the study was between 1998 and 2000]	A randomized controlled trial of a brief intervention for families of patients with a first episode of psychosis (Leavey-UK)<br><br>*Included first-episode psychosis patients who had been diagnosed within the last six months and were recruited from psychiatric services in North London. The intervention group received a brief intervention and treatment as usual. The brief intervention was provided over seven sessions, lasting about one hour, and included: information gathering from the relative, plus sessions on: psychotic illness, symptoms and early warning signs, treatment, help seeking; coping strategies, problem solving and communication with the patient. The control group were given treatment as usual.*	**Efficacy (Phase III)**	**Publication:** Leavey et al 2004<br><br>**Cited on Systematic Review v1, v2 & v3 (2011) [Cochrane Collaboration, Marshall and Rathbone]** as Leavey-UK.<br>V1: No mention<br>V2: Leavey et al 2004 cited as study awaiting assessment<br>V3: **:** Leavey et al 2004<br><br>**Cited on Clinical Guideline (2010) [NICE full guideline published by BPS and RCP]**: Study ID is LEAVEY2004. Paper cited is Leavey et al 2004
1999	Early intervention referenced in **National Service Framework (NSF) for Mental Health (England)**	**National Policy**	**Clinical Guideline Published**
1999-2002<br><br>[NB this was the duration of the study]	Early Detection and Intervention Evaluation (EDIE– UK)<br><br>*Recruited participants from primary care teams, student counselling services, accident and emergency departments, specialist services (community drug and alcohol teams, child and adolescent psychiatry and adult psychiatry*	**Efficacy (Phase III)**	**Publications:** Morrison et al 2002; Morrison et al 2004; French et al 2007; Morrison et al 2007.<br><br>**Cited on Systematic Review v1, v2 & v3 (2011) [Cochrane Collaboration,**

	services) and voluntary sector agencies. Participants had a mean age of 21 years and were judged to have an 'ultra high risk' of developing a first episode of psychosis (Yung's Criteria).		**Marshall and Rathbone]** as EDIE-UK.<br>V1: Cited as ongoing study<br>V2: Morrison et al 2002; Morrison et al 2004<br>V3: French et al 2007; Morrison et al 2007<br><br>**Cited on Clinical Guideline (2010) [NICE full guideline published by BPS and RCP]**: Morrison et al 2004 cited but not in scope ("Early intervention is primarily concerned with identification and initial treatment of people with psychotic illnesses, such as schizophrenia. Identification may be directed either at people in the prodromal phase of the illness ('earlier early intervention') or at those who have already developed psychosis ('early intervention'). Providing treatment for people in a possible prodromal phase of schizophrenia is an interesting but potentially controversial area, which at present is outside the scope of this guideline").
2000	Second international early intervention conference held in New York		[n/a]
2000	COAST – Croydon Outreach and Assertive Support Team (KUIPERS2004-COAST).<br><br>*COAST targeted people in the first 5 years since initial episode of psychosis and offered a range of medical, psychological and vocational interventions, according to need (as opposed to protocol)*	**Efficacy (Phase III)**	**Publication**: Kuipers et al 2004<br><br>**Cited on Systematic Review v1, v2 & v3 (2011) [Cochrane Collaboration, Marshall and Rathbone]** as Kuipers 2004.<br>V1: Not cited<br>V2: Kuipers 2004 cited as a references to a study still awaiting assessment<br>V3: Kuipers 2004 but excluded from study as not first episode<br><br>**Cited on Clinical Guideline (2010) [NICE full guideline published by BPS and RCP]**: Study ID is KUIPERS2004-COAST. Paper cited is Kuipers et al 2004.
2001	OPUS study reported first data (Denmark)<br><br>*Recruited first-episode psychosis (ICD 10) patients from inpatient and outpatient departments in Denmark; participants were aged 18 to 45. Participants received integrated treatment or standard care. Integrated treatment consisted of high fidelity assertive community treatment supplemented by behavioural family therapy and social skills training. Standard care consisted of care at a community mental health centre.*	**Efficacy (Phase III)**	**Publications:** Petersen et al 2005; Bertelsen et al 2008; Jeppesen et al 2005; Jorgensen et al 2000; Nordentoft et al 2002; Nordentoft et al 2006a; Nordentoft et al 2006b; Petersen et al 2007; Petersen et al 2005; Thorup et al 2005; Thorup et al 2006.<br><br>**Cited on Systematic Review v1, v2 & v3 (2011) [Cochrane Collaboration, Marshall and Rathbone]** as OPUS-Scandinavia.<br>V1: Jorgensen et al 2000 cited as an ongoing study<br>V2: Petersen et al 2005; Jeppesen et al 2005; Jorgensen et al 2000; Nordentoft

			et al 2002; Nordentoft et al 2006a; Nordentoft et al 2006b; Thorup et al 2005<br><br>V3: Petersen et al 2005; Bertelsen et al 2008; Jeppesen et al 2005; Jorgensen et al 2000; Nordentoft et al 2002; Nordentoft et al 2006a; Nordentoft et al 2006b; Petersen et al 2007; Petersen et al 2005; Thorup et al 2005; Thorup et al 2006<br><br>**Cited on Clinical Guideline (2010) [NICE full guideline published by BPS and RCP]**: Study ID is PETERSEN2005-OPUS. Papers cited are: Petersen et al 2005; Jeppesen et al 2005; Jorgensen et al 2000; Nordentoft et al 2002; Petersen et al 2007; Petersen et al 2005; Thorup et al 2005; Thorup et al 2006 [Appendix 15: p17/18].
2000-2004<br>*(Study opened in January 2000 with the key paper reporting in 2004)*.	Lambeth Early Onset (LEO-CAT-UK) study of early psychosis services began (England)<br><br>*Included participants with first-episode psychosis (Yung's criteria) with a mean age of 23 years. The study was undertaken in community settings within the borough of Lambeth, London, UK. The design was a cluster-randomised trial in which primary care (GP) practices were randomly allocated to receive training in early detection of psychosis and direct access to LEO-CAT (a specialised treatment team for first-episode psychosis).The control group of General Practice clinics did not receive training in early detection and continued to refer new cases of psychosis to local mental health services who could then refer on to the LEO-CAT programme*	**Efficacy (Phase III)**	**Publications:** Power et al 2004a and b;Craig et al 2004 a and b; Power et al 2007<br><br>**Cited on Systematic Review (2011) [Cochrane Collaboration, Marshall and Rathbone]** as LEO-CAT-UK:<br><br>V1. Cited as on-going study (p26)<br><br>V2: Power et al 2004 a and b, Craig et al 2004a and b cited but excluded as focused on participants with 1st and 2nd episode psychosis<br><br>V3: Power et al 2004 a and b and Power et al 2007 cited and included (not in v2 it was excluded!); Craig et al 2004a and b cited but excluded as focused on participants with 1st and 2nd episode psychosis.<br><br>**Cited on Clinical Guideline (2010) [NICE full guideline published by BPS and RCP]**: Study ID is CRAIG2004. Papers cited are: Craig et al 2004b (as main study) and Garety et al 2006, Garety 2006 and Power et al 2007.
2001-<br><br>[Recruitment took place between January 2001 and January 2004]	EIPS-Germany. Multicentre trial of CBT in the Early Initial Prodromal State (EIPS) at the Early Detection and Intervention Centres located at the Universities of Cologne, Bonn, Dusseldorf and Munich.<br><br>*Note centres were modelled off the Cologne Early Recognition and Intervention Centre (FETZ) noted above and is related to the LIPS-Germany study below (the two studies come from the same centre, were run in parallel but assessment different treatments at different stages]*	**Efficacy (Phase III)**	**Publications**: Bechdolf et al 2004; Bechdolf et al 2007<br><br>**Cited on Systematic Review (2011) [Cochrane Collaboration, Marshall and Rathbone]** as EIPS-Germany:<br><br>V1. No mention<br><br>V2: Bechdolf et al 2004 cited under ongoing study<br><br>V3: Bechdolf et al 2004; Bechdolf et al 2007.<br><br>**Cited on Clinical Guideline (2010) [NICE full guideline published by BPS and**

			**RCP]**: Study ID is BECHDOLF2004. Paper cited is Bechdolf et al 2004
2001-<br><br>[Recruitment began in January 2001 and ended in March 2003, and January 2004 for the two different papers]	LIPS-Germany. Multicentre trial of atypical neuroleptic amisulpride in the Late Initial Prodromal State (LIPS) at the Early Detection and Intervention Centres located at the Universities of Cologne, Bonn, Dusseldorf and Munich<br><br>*Note centres were modelled off the Cologne Early Recognition and Intervention Centre (FETZ) noted above and is related to the EIPS-Germany study below (the two studies come from the same centre, were run in parallel but assessment different treatments at different stages)*	**Efficacy (Phase III)**	**Publications:** Bechdolf et al 2005; Ruhrmann et al 2007<br><br>**<u>Cited</u> on Systematic Review (2011) [Cochrane Collaboration, Marshall and Rathbone]** as LIPS-Germany:<br>V1. No mention<br>V2: Cited as ongoing study (Bechdolf-FETZ)<br>V3: Bechdolf et al 2005; Ruhrmann et al 2007<br><br>*[NB not cited on full NICE guidelines]*
2001-<br><br>*[Recruitment took place between August 2001 and September 2003]*	Active Cognitive Therapy for Early Psychosis (ACE). (Jackson-Australia)<br><br>*Included people with a mean onset of psychosis at 22 years; the settings used were at the participant's home, a neutral location or the EPPIC. The intervention group received CBT with 20 sessions provided for 45minutes, plus antipsychotics. The control group were given a befriending service in addition to antipsychotics.*	**Efficacy (Phase III)**	Publications: Jackson et al 2008<br><br>**<u>Cited</u> on Systematic Review (2011) [Cochrane Collaboration, Marshall and Rathbone]** as Jackson-Australia:<br>V1. No mention<br>V2: No mention<br>V3: Jackson et al 2008<br><br>*[NB not cited on full NICE guidelines]*
2002	Third early intervention international conference held in Copenhagen, Denmark		[n/a]
2002	NICE Clinical guideline: Schizophrenia: core interventions in the treatment and management of schizophrenia in primary and secondary care	**National Policy**	**Clinical Guideline Published:** NICE (CG1 updated in 2010 to CG82)<br><br>*"To date no formal high quality evaluations of the impact of EIS on the initial treatment of psychosis are available" (p199) and goes on to recommend: "It is recommended that early intervention services are evaluated using adequately powered randomised controlled trials reporting all relevant clinical, social, occupational and economic outcomes, including quality of life and longer term outcomes" (p201)*
2005-<br><br>*[Recruitment took place between October 2005 and April 2006]*	Vocational intervention in first-episode psychosis: individual placement and support v. treatment as usual. (Killackey-Australia)<br><br>*Enrolled participants from the EPPIC programme with first-episode psychosis; participants had a mean age of 21 years. The intervention group received individual placement and support, which is an intervention designed to help*	**Efficacy (Phase III)**	Publications: Killackey et al 2008<br><br>**<u>Cited</u> on Systematic Review (2011) [Cochrane Collaboration, Marshall and Rathbone]** as Killackey-Australia:

	people with mental illness to find and keep competitive employment. The support provided in the programme continued after employment was obtained, and was adapted to the needs of the individual. The control group received treatment as usual.		V1. No mention<br>V2: No mention<br>V3: Killackey et al 2008<br><br>*[NB not cited on full NICE guidelines]*
20006	Cochrane Collaboration review – Early Intervention for psychosis published v1<br><br>.	**Research review**	**Systematic review published:** Cochrane Collaboration v1<br><br>Three studies included involving 218 participants: PACE-Melbourne; Linszen-Amsterdam; Zhang-China
2007	Early Intervention in Psychiatry journal launched by IEPA		[n/a]
2010	Cochrane Collaboration review – Early Intervention for psychosis published v2	**Research review**	**Systematic review published:** Cochrane Collaboration v2<br><br>Seven studies included involving 941 participants: PACE-Melbourne; Linszen-Amsterdam; Zhang-China; EDIE-UK; LifeSPAN-Australia; PRIME-USA; OPUS-Scandinavia; PACE-Australia.
2010	NICE Clinical guideline: Schizophrenia: core interventions in the treatment and management of schizophrenia in primary and secondary care<br><br>[NB section on early intervention p74]	**National Policy**	**Clinical Guideline Published:** NICE (CG82)<br><br>Identified four RCTs on early intervention:<br><br>CRAIG2004-LEO; GRAWE2006-OTP; KUIPERS2004-COAST; PETERSEN2005-OPUS
2011	Cochrane Collaboration review – Early Intervention for psychosis published v3<br><br>*Review had two objectives:*<br>*To determine the effects of early detection and treatment of people with 'prodromal' symptoms*<br><br>*To determine the effects of early detection and treatment of people in their first episode of psychosis*	**Research Review**	**Systematic review published:** Cochrane Collaboration v3<br><br>18 studies included involving 1808 participants. Six trials (Amminger-Austria, EIPS-Germany, EDIE-UK, LIPS-Germany, PACE-Australia, PRIME-USA) were concerned with preventing the onset of psychosis. Twelve trials were concerned with improving outcome in first episode psychosis (Alvarez-Spain; Berger-Australia; Edwards-Australia; Jackson-Australia; Killackey-Australia; Leavey- UK; LEO-CAT-UK; LifeSPAN-Australia; Linszen-Amsterdam; OPUS-Scandinavia; Uzenoff-USA; Zhang-China).

EARLY INTERVENTION: (approximately) 30 years from the beginning of research to the NICE recommendation

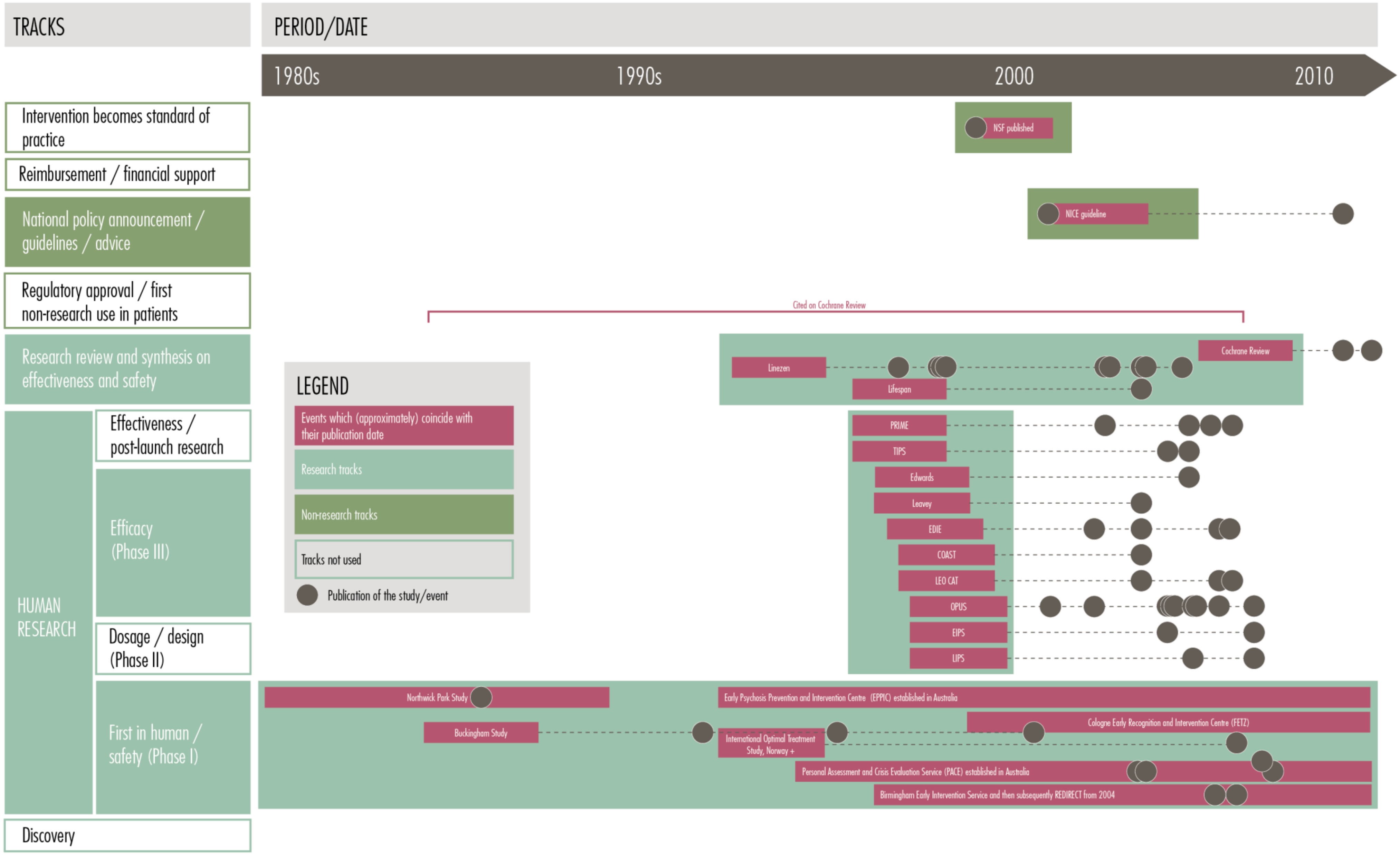

METHODOLOGICAL REFLECTIONS

The approach the case study author took for this case study relied on tracing bibliographic references (citations) manually, but systematically, through the literature using the "Research review" calibration point as the key source for both forward and backward tracing. The key source for this was three versions of the Cochrane Collaboration review. This approach has advantages and disadvantages, especially when compared with other approaches different authors utilised in developing the other six case studies. Perhaps most importantly this approach has the potential to be automated through advanced bibliometric data mining techniques and potentially topic modelling (i.e. the examination of a corpus of literature over time using statistical methods). If such approaches could be developed then, crucially, the approach could be scaled at relatively modest costs. The disadvantage of this approach, however, is it relies exclusively on published (ie dated) publications that are in the public domain and accessible via web searching. One of the observations we made during the project was that the date of a publication may be misleading; it can occur a number of years – sometimes decades – after the research was completed or it can occur very closely to that research. Differential practice in the data of publication makes it difficult to interpret the data. That said publication and the publication date is an indicator of when the research is 'visible' and in that sense it may be an appropriate proxy for measuring elapsed time.

Another observation the case study author made in compiling this case study was the apparent 'connectedness' of the publishing authors – largely around Pat McGorry and colleagues. A hypothesis that could be explored through social network analysis is that the elapsed time between various stages was sufficiently speeded up due to the collaborative network that emanated from Australia.

REFERENCES

Northwick Park Study

Johnstone EC, Crow TJ, Johnson AL ,MacMillan J,. The Northwick Park study of first episodes of schizophrenia. I. Presentation of the illness and problems relating to admission. *British Journal of Psychiatry* 1986;**148**:115–120.

Crow TJ, MacMillan J, Johnson AL, Johnstone EC. The Northwick Park study of first episodes of schizophrenia: ii. A randomised controlled trial of prophylactic neuroleptic treatment. *British Journal of Psychiatry* 1986;**148**:120–7.

Buckingham study

Falloon, Ian R. Early intervention for first episodes of schizophrenia: A preliminary exploration. Psychiatry: Interpersonal and Biological Processes, Vol 55(1), Feb 1992, 4-15.

Falloon I. General practice recruitment for people at risk of schizophrenia: the Buckingham experience. *Australian & New Zealand Journal of Psychiatry (suppl)* 2000;**34 Suppl**:S131-6; discussion S140-4.

Falloon et al 1996. Early detection and intervention for initial episodes of schizophrenia. Schizophrenia Bulletin, 22:2. 271-282.

PACE-Australia

McGorry PD, Yung AR, Phillips LJ, Yuen HP, Francey S, Cosgrave EM, Germano D, Bravin J, McDonald T, Blair A, Adlard S, Jackson H. Randomized controlled trial of interventions designed to reduce the risk of progression to first-episode psychosis in a clinical sample with subthreshold symptoms. *Archives of General Psychiatry* 2002;**59**(10): 921–8.

Phillips LJ, Leicester SB, O'Dwyer LE, Francey SM, Koutsogiannis J, Abdel-Baki A, Kelly D, Jones S, Vay C, Yung AR, McGorry PD. The PACE Clinic: identification and management of young people at 'ultra' high risk of psychosis. *Journal PsychiatrIc Practice* 2002;**8**(5):255–69

Phillips LJ, McGorry PD, Yuen HP, Ward J, Donovan K Kelly D, Francey SM, Yung AR. Medium term follow-up of a randomized controlled trial of interventions for young people at ultra high risk of psychosis. *Schizophrenia Research* 2007;**96**(1-3):25–33

Phillips LJ, Yung AR, Yuen HP, Pantelis C, McGorry PD. Prediction and prevention of transition to psychosis in young people at incipient risk for schizophrenia. *American Journal of Medical Genetics* 2002;**114**(8):929–37

REDIRECT study

Lester HE, Birchwood M, Tait L, Freemantle N. Redirect: evaluating the effectiveness of an educational intervention about first episode psychosis in primary care. *Schizophrenia Research* 2006;**86**(Suppl 1):S39–40.

Tait L, Lester H, Birchwood M, Freemantle N, Wilson S. Design of the Birmingham early detection in untreated psychosis trial (REDIRECT): cluster randomised controlled trial of general practitioner education in detection of first episode psychosis [ISRCTN87898421]. *BMC Health Services Research* 2005;**5**(1):19.

PRIME-USA

McGlashan TH, Zipursky RB, Perkins D, Addington J, Miller T, Woods SW, Hawkins KA, Hoffman RE, Preda A, Epstein I, Addington D, Lindborg S, Trzaskoma Q, Tohen M, Breier A. Randomized, double-blind trial of olanzapine versus placebo in patients prodromally symptomatic for psychosis. *American Journal of Psychiatry* 2006;**163**(5): 790–9.

Hawkins KA, Keefe RSE, Christensen BK, Addington J, Woods SW, Callahan J, Zipursky RB, Perkins DO, Tohen M, Breier A, McGlashan TH. Neuropsychological course in the prodrome and first episode of psychosis: findings from the prime North America double blind treatment study. *Schizophrenia Research* 2008;**105**(1-3):1–9.

Hoffman RE, Woods SW, Hawkins KA, Pittman B, Tohen M, Preda A, Breier A, Glist J, Addington J, Perkins DO, McGlashan TH. Extracting spurious messages from noise and risk of schizophrenia-spectrum disorders in prodromal population. *British Journal of Psychiatry* 2007;**191**(2): 355–6.

McGlashan TH, Zipursky RB, Perkins D, Addington J, Miller TJ, Woods SW, Hawkins KA, Hoffman R, Lindborg S, Tohen M, Breier A. The prime North America randomized double-blind clinical trial of olanzapine versus placebo in patients at risk of being prodromally symptomatic for psychosis. I. Study rationale and design. *Schizophrenia Research* 2003;**61**(1):7–18.

TIPS-Norway

Melle I, Johannesen JO, Friis S, Haahr U, Joa I, Larsen TK, Opjordsmoen S, Rund BR, Simonsen E, Vaglum P, McGlashan T. Early detection of the first episode of schizophrenia and suicidal behavior. *American Journal of Psychiatry* 2006;**163**(5):800–4.

Melle I, Larsen TK, Haahr U, Friis S, Johannessen JO, Opjordsmoen S, Simonsen E, Rund BR, Vaglum P, McGlashan T. Reducing the duration of untreated first episode psychosis: effects on clinical presentation. *Archives of General Psychiatry* 2004;**61**(2):143–50.

OPUS-Scandanavia

Petersen L, Jeppesen P, Thorup A, Abel MB, Ohlenschlaeger J, Christensen TO, Krarup G, Jorgensen P, Nordentoft M. A randomised multicentre trial of integrated versus standard treatment for patients with a first episode of psychotic illness. *BMJ* 2005;**331**(7517):602.

Bertelsen M, Jeppesen P, Petersen L, Thorup A, Ohlenschlaeger J, le Quach P, Christensen TO, Krarup G, Jorgensen P, Nordentoft M. Five-year follow-up of a randomized multicenter trial of intensive early intervention vs standard treatment for patients with a first episode of psychotic illness: the OPUS trial. *Archives of General Psychiatry* 2008;**65**(7):762–71.

Jeppesen P, Petersen L, Thorup A, AbelMB, Oehlenschlaeger J, Christensen TO, Krarup G, Hemmingsen R, Jorgensen P, Nordentoft M. Integrated treatment of first-episode psychosis: effect of treatment on family burden: OPUS trial. *British Journal of Psychiatry* 2005;**48**:s85–90.

Jeppesen P, Petersen L, Thorup A, AbelMB, Ohlenschlaeger J, Christensen TO, Krarup G, Jorgensen P, Nordentoft M. The association between pre-morbid adjustment, duration of untreated psychosis and outcome in first-episode psychosis. *Psychological Medicine* 2008;**8**:1157–66.

Jorgensen P, Nordentoft M, Abel M, Gouliaev G, Jeppesen P, Kassow P. Early detection and assertive community treatment of young psychotics: the opus study rationale and design of the trial. *Social Psychiatry & Psychiatric Epidemiology* 2000;**35**:283–7.

Nordentoft M, Jeppesen P, Abel M, Kassow P, Petersen L, Thorup A, Krarup G, Hemmingsen R, Jorgensen P. OPUS study: suicidal behaviour, suicidal ideation and hopelessnessamong patients with first-episode psychosis. One-year follow-up of a randomised controlled trial. *British Journal of Psychiatry* 2002;**43**:s98–106.

Nordentoft M, Petersen L, Jeppesen P, Thorup AA, Abel MB, Ohlenschlaeger J, Christensen TO, Krarup G, Jorgensen P. OPUS: a randomised multicenter trial of integrated versus standard treatment for patients with a firstepisode psychosis--secondary publication. *Ugeskr Laeger* 2006;**168**(4):381–4.

Nordentoft M, Thorup A, Petersen L, Ohlenschlaeger J, Melau M, Christensen TO, Krarup G, Jorgensen P, Jeppesen P. Transition rates from schizotypal disorder to psychotic disorder for first-contact patients included in the opus trial. A randomized clinical trial of integrated treatment and standard treatment. *Schizophrenia Research* 2006;**83**(1): 29–40.

Petersen L, Jeppesen P, Thorup A, Ohlenschlaeger J, Krarup G, Ostergard T, Jorgensen P, Nordentoft M. Substance abuse and first-episode schizophrenia-spectrum disorders The Danish opus trial. *Early Intervention in Psychiatry* 2007; **1**(1):88–96.

Petersen L, Nordentoft M, Jeppesen P, Ohlenschaeger J, Thorup A, Christensen TO, Krarup G, Dahlstrom J, Haastrup B, Jorgensen P. Improving 1-year outcome in firstepisode psychosis: Opus trial. *British Journal of Psychiatry* 2005;**48**(Suppl):s98–103.

Thorup A, Petersen L, Jeppesen P, Ohlenschlaeger J, Christensen T, Krarup G, Jorgensen P, Nordentoft M. Integrated treatment ameliorates negative symptoms in first episode psychosis - results from the Danish opus trial. *Schizophrenia Research* 2005;**79**(1):95–105.

Thorup A, Petersen L, Jeppesen P, Ohlenschlaeger J, Christensen T, Krarup G, Jorgensen P, Nordentoft M. Social network among young adults with first-episode schizophrenia spectrum disorders: results from the Danish opus trial. *Social Psychiatry and Psychiatric Epidemiology* 2006;**41**(10):761–70.

LEO-UK
Craig T, Garety P, Power P, Rahaman N, Colbert S, Fornells- Ambrojo M. Lambeth early onset service: a randomisedcontrolled trial. *Schizophrenia Research* 2004;**70**(1):145–6.

Craig TKJ, Garety P, Power P, Rahaman N, Colbert S, Fornells-Ambrojo M, Dunn G. The Lambeth early onset (LEO) Team: randomised controlled trial of the effectiveness of specialised care for early psychosis. *BMJ* 2004;**329**(7474):1067–70.

Power P, Craig T, Mcguire P, Iacoponi E, Garety P, Russell M. A randomised controlled trial of an early detection team in first episode psychosis: the leo cat trial. *Schizophrenia Research* 2004;**67**(1):36.

Power P, Iacoponi E, Reynolds N, Fisher H, Russell M, Garety P, McGuire PK, Craig T. The Lambeth early onset crisis assessment team crisis assessment study: general practitioner education and access to an early detection team in first-episode psychosis. *British Journal of Psychiatry* 2007; **Supp 51**:s133–9.

Power P, Iacoponi E, Russell M, Fisher H,Mcguire P, Garety P, Valmaggio L, Craig T. A randomised controlled trial of an early detection team in first- episode psychosis: provisional findings of the leo cat study. *Schizophrenia Research* 2004; **70**(1):131.

Garety,P.A. Erratum: "Specialised care for early psychosis: Symptoms, social functioning and patient satisfaction: Randomised controlled trial". *British Journal of Psychiatry* 2006;188(**3**): Mar06.

Garety,P.A.; Craig,T.K.; Dunn,G.; Fornells-Ambrojo,M.; Colbert,S.; Rahaman,N.; Read,J.; Power,P. Specialised care for early psychosis: symptoms, social functioning and patient satisfaction: randomised controlled trial. *British Journal of Psychiatry* 2006;188: 37 - 45.

Power,P.; McGuire,P.; Iacoponi,E.; Garety,P.; Morris,E.; Valmaggia,L.; Grafton,D.; Craig,T. Lambeth early onset (LEO) and outreach & support in south London (OASIS) service. *Early Intervention in Psychiatry* 2007;1(**1**): 103.

COAST
Kuipers E.; Holloway F.; Rabe-Hesketh S.; Tennakoon L. An RCT of early intervention in psychosis: Croydon Outreach and Assertive Support Team (COAST). *Social Psychiatry and Psychiatric Epidemiology* 2004;39/5(**358-363**)

Norway-PTP
Grawe,R.W.; Falloon,I.R.; Widen,J.H.; Skogvoll,E. Two years of continued early treatment for recent-onset schizophrenia: a randomised controlled study. *Acta Psychiatrica Scandinavica* 2006;114(**5**): 328 - 336.

Morken,G.; Grawe,R.W.; Widen,J.H. Effects of integrated treatment on antipsychotic medication adherence in a randomized trial in recent-onset schizophrenia. *Journal of Clinical Psychiatry* 2007; 68(**4**): 566 - 571.

Linszen – Amsterdam [SR = http://www.sciencedirect.com/science/journal/09209964]
Linszen D, Dingemans P, Lenior ME. Early intervention and a five year follow up in young adults with a short duration of untreated psychosis:ethical implications. *Schizophrenia Research* 2001;**51**:55– 61.

Linszen D, Dingemans P, Van Der Does JW, Nugter A, Scholte P, Lenior R, Goldstein MJ. Treatment, expressed emotion and relapse in recent onset schizophrenic disorders. *Psychological Medicine* 1996; **26**(2):333–42.

Linszen D, Dingemans PM, Lenior ME, Scholte WF, Goldstein M. Early family and individual interventions and relapse in recent-onset schizophrenia and related disorders. *Italian Journal of Psychiatry & Behavioural Sciences* 1998:77–84.

Linszen D, Dingemans PM, Scholte WF, Lenior ME, Goldstein M. Early recognition, intensive intervention and other protective and risk factors for psychotic relapse in patients with first psychotic episodes in schizophrenia. *International Clinical Psychopharmacology* 1998;**13**(Suppl):S7–S12.

Linszen D, Lenior M, De Haan L, Dingemans P, Gersons, B. Early intervention, untreated psychosis and the course of early schizophrenia. *British Journal of Psychiatry - Supplement* 1998;**172** (Suppl):84–9.

Lenior ME, Dingemans PM, Schene AH, Linszen DH. Predictors and five-year outcome in early-onset schizophrenia. A path analysis. *Schizophrenia Research* 2003;**60**:292.

Lenior ME, Dingemans PMAJ, Schene AH, Linszen DH. Predictors of the early 5-year course of schizophrenia: a path analysis. *Schizophrenia Bulletin* 2005;**31**(3):781–91.

Linszen D. Early and critical period intervention in first episode schizophrenia: relapse, chronicity, early stabilisation, predictors over 4 years and new research. *Schizophrenia Research* 2004;**70**(1):66.

Linszen D, Wouters L, Dingemans P, De Haan L, Nieman D. Early and 3-year sustained intervention in first episode schizophrenia: relapse, stabilization and its predictors. *Schizophrenia Research* 2004;**67**(1):18.

Linszen DH, De Haan L, Dingemans P, Van Bruggen M, Hofstra N, Van Engelsdorp H, Smitten M. Treatment reluctance in first episode schizophrenia: Lack of insight, non-compliance and cannabis abuse predict bad outcome after eighteen months intervention. *Schizophrenia Research* 2003;**60**:325.

Zhang-China

Zhang M, Wang M, Li J, Phillps MR. Randomised-control trial of family intervention for 78 first-episode male schizophrenic patients: an 18-month study in suzhou, jiangsu. *British Journal of Psychiatry* 1994;**165**(Suppl 24):96–102

EDIE-UK

Morrison AP, French P,Walford L, Lewis SW, Kilcommons A, Green J, Parker S, Bentall RP. Cognitive therapy for the prevention of psychosis in people at ultra-high risk: randomised controlled trial. *British Journal of Psychiatry* 2004;**185**:291–7.

Morrison AP, Bentall RP, French P, Walford L, Kilcommons A, Knight A, Kreutz M, Lewis SW. Randomised controlled trial of early detection and cognitive therapy for preventing transition to psychosis in high-risk individuals. Study design and interim analysis of transition rate and psychological risk factors. *British Journal of Psychiatry* 2002;**43**:s78–84.

French P, Shryane N, Bentall RP, Lewis SW, Morrison AP. Effects of cognitive therapy on the longitudinal development of psychotic experiences in people at high risk of developing psychosis. *British Journal of Psychiatry – Supplementum* 2007;**51**:s82–7.

Morrison AP, French P, Parker S, Roberts M, Stevens H, Bentall RP, Lewis SW. Three-year follow-up of a randomized controlled trial of cognitive therapy for the prevention of psychosis in people at ultrahigh risk. *Schizophrenia Bulletin* 2007;**33**(3):682–7.

LifeSPAN-Australia

Power PJ, Bell RJ, Mills R, Herrman-Doig T, Davern M, Henry L, Yuen HP, Khademy-Deljo A, McGorry PD. Suicide prevention in first episode psychosis: the development of a randomised controlled trial of cognitive therapy for acutely suicidal patients with early psychosis. *Australian and New Zealand Journal of Psychiatry* 2003;**37**(4):414–20.

EIPS-Germany

Bechdolf A, Ruhrmann S, Janssen B, Bottlender R, Wagner M, Maurer K, Hafner H, Maier W, Klosterkotter J. Early recognition and intervention for people at risk of schizophrenia [FRUHERKENNUNG UND – INTERVENTION BEI PERSONEN MIT ERHOHTEM PSYCHOSERISIKO]. *Psychoneuro* 2004;**30**(11):606–14.

Bechdolf A, Wagner M, Veith V, Ruhrmann S, Pukrop R, Brockhaus-Dumke A, Berning J, Stamm E, Janssen B, Decker P, Bottlender R, Moller H-J, Gaebel W, Maier W, Klosterkotter J. Randomized controlled multicentre trial of cognitive behaviour therapy in the early initial prodromal state: effects on social adjustment post treatment. *Early Intervention in Psychiatry* 2007;**1**(1):71–8.

LIPS-Germany

Bechdolf A, Ruhrmann S, Wagner M, Kuhn KU, Janssen B, Bottlender R, Wieneke A, Schulze-Lutter F, Maier W, Klosterkotter J. Interventions in the initial prodromal states of psychosis in Germany: concept and recruitment. *British Journal of Psychiatry Supplementum* 2005;**187**(Suppl 48): s45–8.

Ruhrmann S, Bechdolf A, Kuhn KU, Wagner M, Schultze-Lutter F Janssen B, Maurer K, Hafner H, Gaebel W, Moller HJ, Maier W, Klosterkotter J, LIPS study group. Acute effects of treatment for prodromal symptoms for people putatively in a late initial prodromal state of psychosis. *British Journal of Psychiatry Supplementum* 2007;**51**:s88–95.

Edwards-Australia
Edwards J, Elkins K, Hinton M, Harrigan SM, Donovan K, Athanasopoulos O, McGorry PD. Randomized controlled trial of a cannabis-focused intervention for young people with first-episode psychosis. *Acta Psychiatrica Scandinavica* 2006;**114**:109–17

Jackson-Australia
Jackson HJ, McGorry PD, Killackey E, Bendall S, Allott K, Dudgeon P, Gleeson J, Johnson T, Harrigan S. Acute-phase and 1-year follow-up results of a randomized controlled trial of CBT versus befriending for first-episode psychosis: the ACE project. Psychological Medicine 2008; 38(5):725–35.

Killackey-Australia
Killackey E, Jackson HJ, McGorry PD. Vocational intervention in first-episode psychosis: individual placement and support versus treatment as usual. *British Journal of Psychiatry* 2008;**193**(2):114–20.

Leavey-UK
Leavey G, Gulamhussein S, Papadopoulos C, Johnson-Sabine E, Blizard BKM. A randomized controlled trial of a brief intervention for families of patients with a first episode of psychosis. *Psychological Medicine* 2004;**34**(3):423–31.
